# A Thermodynamic Atlas of Proteomes Reveals Energetic Innovation across the Tree of Life

**DOI:** 10.1093/molbev/msac010

**Published:** 2022-01-17

**Authors:** Alexander F Chin, James O Wrabl, Vincent J Hilser

**Affiliations:** 1 Department of Biology, Johns Hopkins University, Baltimore, MD, USA; 2 T.C. Jenkins Department of Biophysics, Johns Hopkins University, Baltimore, MD, USA

**Keywords:** evolution, thermodynamics, proteome, protein stability, bioinformatics

## Abstract

Protein stability is a fundamental molecular property enabling organisms to adapt to their biological niches. How this is facilitated and whether there are kingdom specific or more general universal strategies are unknown. A principal obstacle to addressing this issue is that the vast majority of proteins lack annotation, specifically thermodynamic annotation, beyond the amino acid and chromosome information derived from genome sequencing. To address this gap and facilitate future investigation into large-scale patterns of protein stability and dynamics within and between organisms, we applied a unique ensemble-based thermodynamic characterization of protein folds to a substantial portion of extant sequenced genomes. Using this approach, we compiled a database resource focused on the position-specific variation in protein stability. Interrogation of the database reveals: 1) domains of life exhibit distinguishing thermodynamic features, with eukaryotes particularly different from both archaea and bacteria; 2) the optimal growth temperature of an organism is proportional to the average apolar enthalpy of its proteome; 3) intrinsic disorder content is also proportional to the apolar enthalpy (but unexpectedly not the predicted stability at 25 °C); and 4) secondary structure and global stability information of individual proteins is extractable. We hypothesize that wider access to residue-specific thermodynamic information of proteomes will result in deeper understanding of mechanisms driving functional adaptation and protein evolution. Our database is free for download at https://afc-science.github.io/thermo-env-atlas/ (last accessed January 18, 2022).

## Introduction

Although protein sequence and secondary structure have been analyzed extensively in the study of protein evolution, neither primary sequence nor secondary structure information report on the underlying energetics that ultimately shape macromolecular or organismal evolution. Rather, a combination of steric considerations, van der Waals interactions, hydrogen bonding, and hydrophobic effects, among others, are contextualized by the physical constraints of a cell, a tissue, and the surroundings of an organism. These complex phenomena result in a balance of finely tuned thermodynamic stabilities that may or may not allow formation of secondary structure elements (Srinivasan and Rose 1999). Therefore, although folded proteins may be constructed out of conserved domains or motifs, in the absence of high-throughput effective atomic force fields, the provisional understanding of how physical constraints have driven protein and proteome evolution will require a thermodynamic description that is independent of secondary structural classification (Alva et al. 2015).

**Fig. 1.**
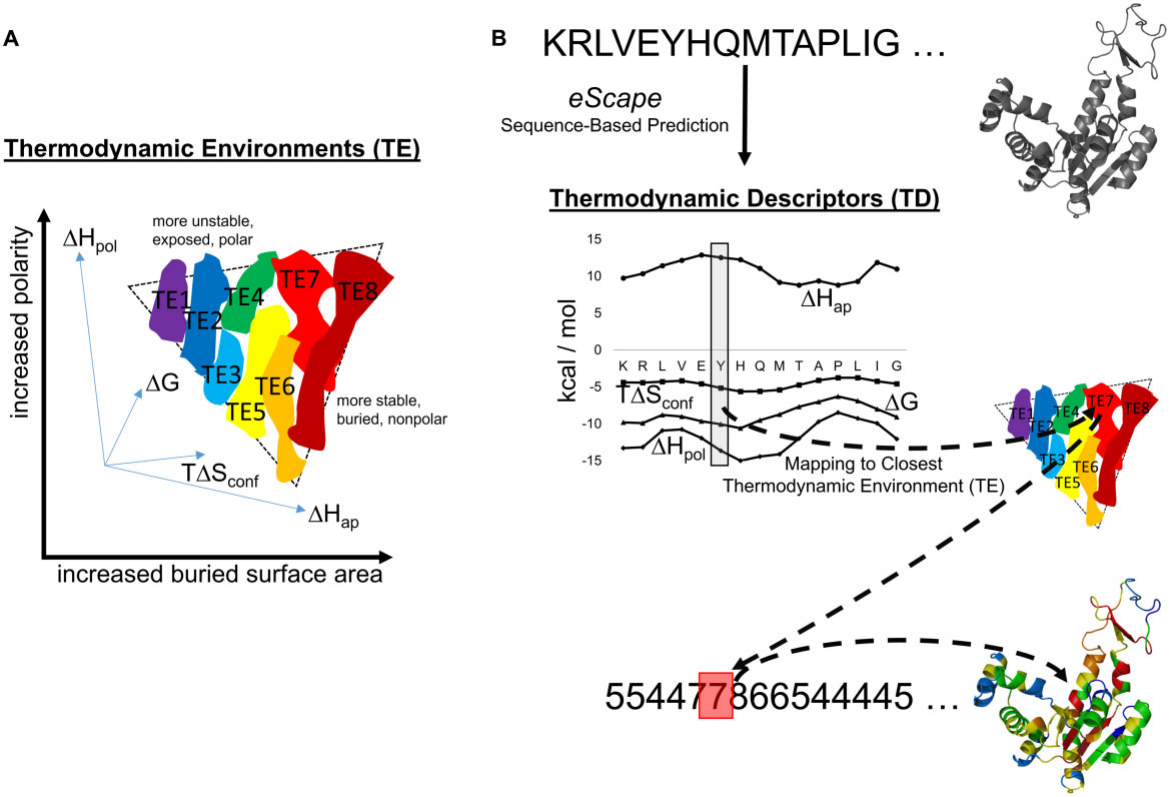
Estimating TEs in proteins from *eScape* sequence-based TDs. (*A*) A summary of what is known about TEs in the native state of proteins. Light blue axes represent a high-dimensional thermodynamic space (4D) that can be decomposed into a physically interpretable low-dimensional space (2D) represented by thick black axes. Every residue of any protein structure can be plotted within this space using ensemble-based modeling. When this is done for a large database of proteins, all residues can be clustered into eight significant regions (colored irregular shapes). These regions exhibit specific combinations of enthalpy and entropy, are termed “thermodynamic environments” (TE), and can be roughly organized by relative stability. Rainbow colors and numbers depict relative stability, ranging from lowest stability TE1 (purple) to highest stability TE8 (dark red). Dashed triangle depicts the approximate shape of 2D space with respect to physical properties (Vertrees et al. 2009). (*B*) *eScape* is a sequence-based predictor of stability, enthalpy, and entropy of proteins (Gu and Hilser 2008). For every residue in any protein sequence, the output of *eScape* (gray box) can be mapped to a TE (dashed lines). Thus, a complex description of protein thermodynamics can be simplified to a 1D string equal to the number of residues in the protein. The example protein molecular cartoon shown is *Escherichia coli* AK (*apo*). Note that this workflow does not depend on the existence of an experimental protein structure.

To that end, we computed a database of position-specific thermodynamic information for each residue of each protein in a library of organisms across the tree of life. We assign a Gibbs free energy (Δ*G*), apolar enthalpy (Δ*H*_apolar_), polar enthalpy (Δ*H*_polar_), and conformational entropy (*T*Δ*S*_conf_) to each residue position, which can be thought of summarizing the contributions of van der Waals interactions, hydrogen bonding, charge–charge interactions, hydrophobic effects, conformational flexibility, and other effects, to the local stability across a protein chain (fig. 1). Importantly, as opposed to providing the energetic contribution of each residue to the stability of the protein, this local thermodynamic description reports on the stability at each position much in the same way as the Protein Data Bank (Berman et al. 2000) reports on the secondary and tertiary structure of each position (Wrabl et al. 2001, 2002; Larson and Hilser 2004; Gu and Hilser 2008). Furthermore, because this energetic representation is orthogonal to structural characterizations (Vertrees et al. 2009), it provides a vehicle for exploring evolutionary relationships between sequences and folds that transcend sequence and structural similarity.

Leveraging the thermodynamic information in the database revealed several noteworthy observations. First, proteomes across the three domains of life assumed a broad monomodal distribution of site-specific thermodynamics, such that organism-specific enrichment roughly discriminated between the domains. Second, this taxonomic trichotomy was partially accounted for by organismal growth temperature and intrinsic disorder content, both of which could be predicted by a principal component decomposition of the site-specific thermodynamics. Third, properties of individual proteins, such as secondary structure content and global stability, could be estimated solely from site-specific thermodynamics. We anticipate that additional insights can be drawn from this unique database resource.

## Results

### Thermodynamic Information Is Fundamentally Different than Sequence Information

**Fig. 2.**
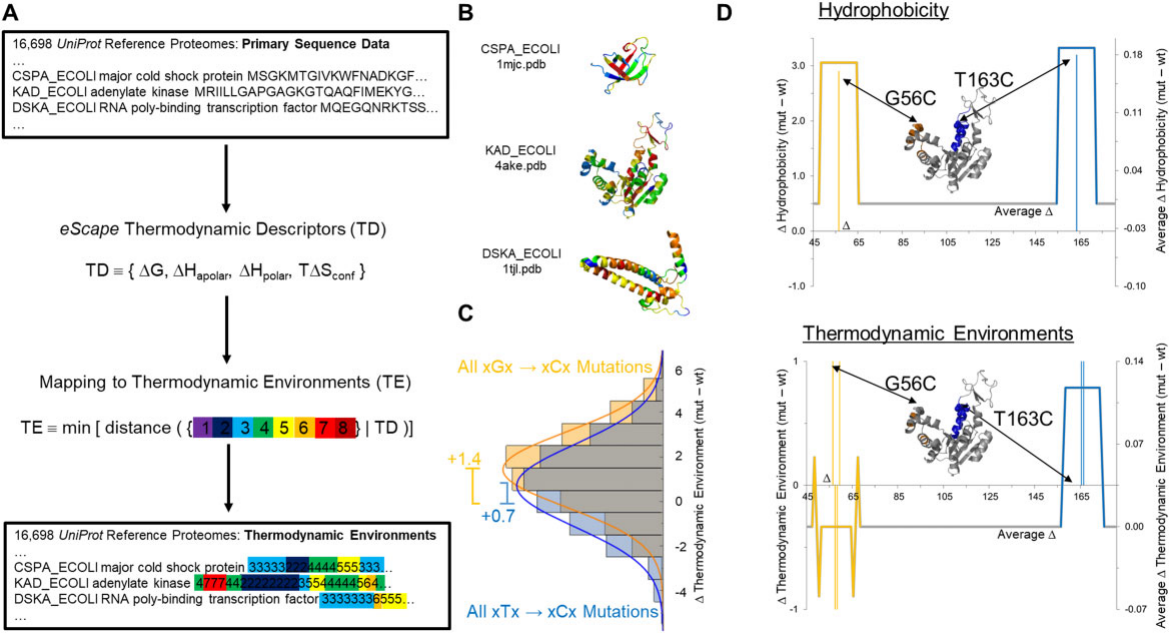
Construction of residue-specific thermodynamic database of proteomes. (*A*) Workflow input and output are shown in boxes. A proteome’s primary sequence data is input into the *eScape* algorithm, and TDs are computed as an intermediate step (first arrow). A second step (second arrow) coarse-grains the TD values into an “eight-letter alphabet” (colors) of TEs, described in figure 1*B*. These TE values are output into the database, associated with the original proteome annotation. (*B*) Three *Escherichia coli* proteins are shown as examples. Coloring is defined in figures 1 and 2*A*, and clearly shows that neither secondary structure elements nor loops are expected to be uniform in stability. Note that this workflow does not depend on the existence of an experimental protein structure. For simplicity, the panel depicts native state data only, even though denatured state data are also included in the database. (*C*) Populations of the thermodynamic consequences of all G → C (orange) and all T → C (blue) substitutions greatly depend on sequence context. The expected value of any G → C or T → C mutation is stabilizing (+0.7 and +1.4 environment, respectively), but the large variances indicate that many substitutions could actually be destabilizing. Such consequences are illustrated on a real-life example in (*D*). (*D*) Sequence changes may have different hydrophobic and thermodynamic consequences. *Escherichia coli* AK changes in hydrophobicity (top) and native state TE (bottom) are shown as the result of two mutations G56C (orange) and T163C (blue) (Kovermann et al. 2017). Both mutations increase the hydrophobicity (Kyte and Doolittle 1982) at the site of the mutation (thin vertical lines, top) over a typical moving average window (17 residues, thick continuous line). In contrast, only the mutation T163C has similar thermodynamic consequences by uniformly increasing the local stability (blue thin vertical line, bottom), although this increase does not occur at position 163 but rather at two positions C-terminal. As described in the Main Text, the G56C mutation directly affects residues 57, 58, 59 as well as residue 56 (orange thin vertical lines, bottom). Moreover, window averaging unexpectedly obscures any changes at this mutation site by pushing the compensating effects to the window edges (orange thick continuous line, bottom).

We used the full set of *Uniprot* Reference Proteomes to construct a comprehensive, nonredundant, sequence-based energetic profile of each protein within each proteome, regardless of the existence of tertiary structure (fig. 2*A* and *B*). The profiling procedure, developed previously in our research group and named *eScape* (i.e. energetic landscape), computes position-specific thermodynamic descriptors (TDs) Δ*G*, Δ*H*_apolar_, Δ*H*_polar_, *T*Δ*S*_conf_ for each residue in a protein sequence (Gu and Hilser 2008) (fig. 1*B*). The delta in these descriptors refers not to the difference between fully folded and fully unfolded states but rather to the difference between subensembles in which the residue is folded or unfolded without regard to the rest of the protein (Hilser and Freire 1996). Perhaps uniquely among bioinformatics tools, *eScape* computes these TDs for both the native state and a specific, locally unfolded denatured state of the protein simultaneously. The vector of TDs is then objectively assigned to a coarse-grained bin, or cluster, termed a “thermodynamic environment” (TE) such that each residue position is mapped to one of eight unique TEs (Hoffmann et al. 2016) (fig. 1*A*). Importantly, the TDs have been experimentally benchmarked (Hilser and Freire 1996; Whitten et al. 2005; Liu et al. 2012) and the TEs have been previously shown to be useful in fold recognition (Wrabl et al. 2002; Wang et al. 2008; Hoffmann et al. 2016).

We emphasize that although the TE positional mapping resulting from this procedure is isomorphic to the amino acid sequence, its semantic mapping is not. In other words, the TE sequence is an orthogonal and distinct descriptor from the amino acid sequence and cannot be considered equivalent, or converted, by a simple substitution (Larson and Hilser 2004; Vertrees et al. 2009). Two reasons for this are that 1) the *eScape* algorithm considers sequence context (i.e. using triplets, instead of single amino acids), as the input for its predictions and 2) *eScape* was trained on structure-based ensemble data, which indirectly incorporates nonlocal contributions to protein stability. Thus, when compared with primary sequence, TEs represent a novel annotation that could potentially provide different, yet complementary, information to existing databases.

As an example to illustrate this point, we consider the essential *Escherichiacoli* protein adenylate kinase (AK) (Muller et al. 1996; Couñago et al. 2008), which has been engineered to contain the double mutation G56C/T163C (Kovermann et al. 2017). Typical databases that compute the sequence-based hydrophobicity profiles (Kyte and Doolittle 1982) of the wild-type and engineered proteins, would conclude that the hydrophobicity at both positions had increased by the same average amount (fig. 2*D*, top). In contrast, the TEs for these two proteins show different and distinct stability effects that are not remedied by averaging (fig. 2*D*, bottom).

The origin of this difference is due to the nature of the information harnessed by *eScape*. The sequence-based thermodynamics computed by *eScape* were derived from statistical analysis of every possible tripeptide to be in each TE, as sampled from a large nonredundant protein structure database. At this tripeptide level, it is important to note that long-range electrostatics are not explicitly captured. However, charge interactions are taken into account in an average, statistical way in the *eScape* parameterization, to the extent that specific pairwise interactions (e.g. salt bridges) repeatedly and nonrandomly occur in globular proteins. It is often the case that changing one amino acid in the tripeptide significantly changes the observed distribution of that tripeptide across the different TEs in the protein database. In such cases *eScape* would predict a large change. In essence, and importantly, *eScape* projects the impact of a particular type of mutation, averaged over the entire database, onto a single sequence being investigated.

For the AK double cysteine example, distributions of the predicted thermodynamic consequences of all possible G → C and T → C substitutions observed in the PDB demonstrate a range of effects (fig. 2*C*). Although the expected effect for both point mutants is to increase the local stability at the site by approximately 1 kcal/mol, as seen for T163C, the exact effects depend on the neighboring amino acids and could in many cases be destabilizing, as seen for G56C (fig. 2*C* and *D*).

Thus, the thermodynamic predictions could contain useful information not captured by traditional sequence analysis, granting an unprecedented ability to incorporate protein energetics into phylogenetic analysis. Because these TEs reflect equilibrium fluctuations in local stability that are important for function (Whitten et al. 2005; Gu and Hilser 2009), they represent a key determinant of molecular evolution (Saavedra et al. 2018), a determinant that has been largely, albeit inadvertently, excluded from existing phylogenetics. Moreover, it is easy to imagine that large numbers of primary sequence changes, such as between homologous proteins of remotely related organisms, might amplify such cryptic thermodynamic effects.

### TEs Content of Proteomes from All Kingdoms of Life

**Fig. 3.**
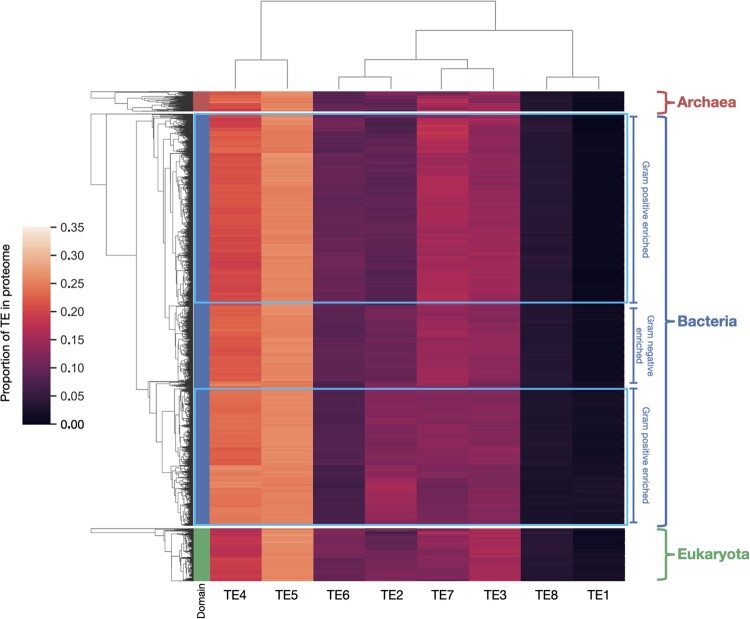
Hierarchical clustering of native TE occurrence frequency, by proteome. Each row corresponds to one of 10,520 proteomes analyzed. Coloring in the far left column corresponds to the domain of the organism (blue = bacteria, green = eukaryote, red = archaea). Regardless of the domain of life, TEs naturally vary in their usage frequency from organism to organism. TE4 and TE5, the median two TEs by stability, occur approximately two to three times more frequently than any other given TE. The extremes of TE stability, TE1 and TE8, occur least frequently. Clustering on the left hand side was done independently for each kingdom. There is a modest co-occurrence between visual clustering and preponderance of Gram-staining in bacteria (labels).

We set out to investigate the large-scale usage of native TEs across a wide sampling of the kingdoms of life. To this end, we used hierarchical clustering to examine whether various organisms used greater or fewer of certain TEs in their proteomic composition (fig. 3). We found that, as a general rule, no organism or group of organisms were equipped with a proteome sampled from a flat, equiprobable TE distribution. Instead, distribution statistics for individual TEs were markedly peaked. The most frequently used native state TEs were those of median stability, TE4 and TE5, each typically accounting for >20% of a proteome. The least frequently used TEs were those of the most extreme stabilities, TE1 and TE8, corresponding to the least and the most stable, respectively. Median stability TEs were observed at least about twice as often as any other given TE, an observation supported by the branching cluster tree distinguishing their usage (fig. 3, top).

**Fig. 4.**
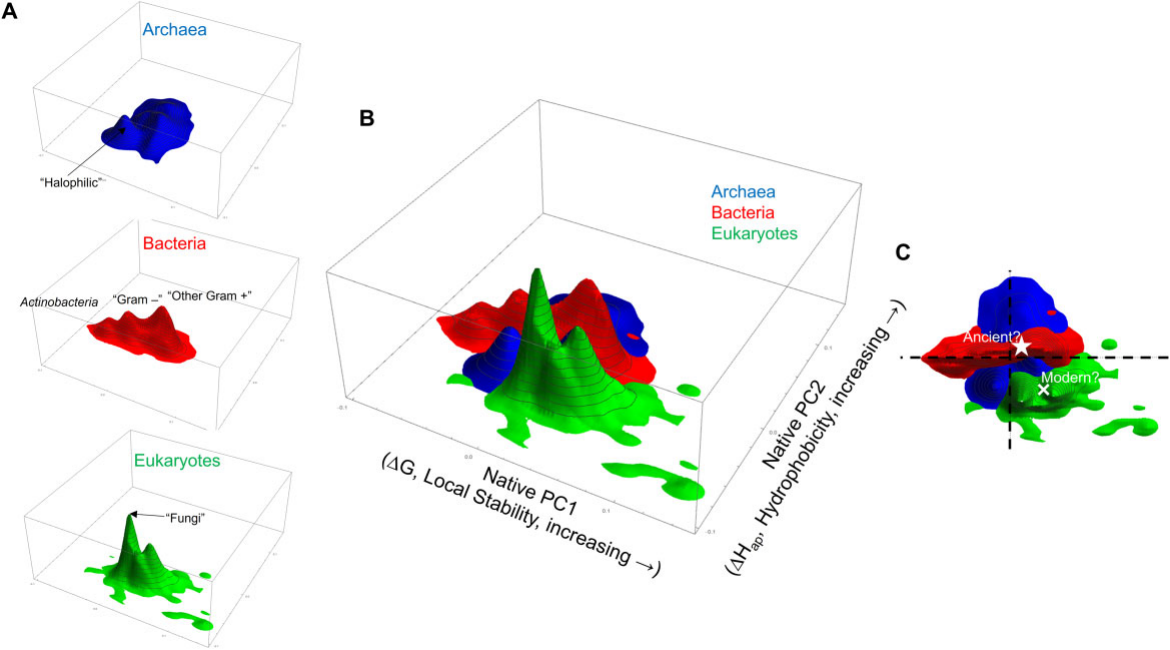
PCA of TE occurrence frequency, by proteome. PCA reveals distinct regions that distinguish between each domain of life; 72.6% of variance is explained by principal component 1 (PC1), 22.0% by PC2, and 2.4% by PC3 (not shown). As discussed in the main text, PC1 can be interpreted as a local stability and PC2 can be interpreted as a hydrophobicity (labeled axes in [*B*]). Data in all panels reflect smoothed kernel scaled densities to reduce visual artifacts caused by unequal proteome density among kingdoms: bacteria are colored red, archaea are blue, and eukaryotes green. (*A*) Each kingdom is highlighted separately for clarity. Major characteristics of organisms comprising the prominent peaks are labeled. (*B*) Merge of the separate panels in (*A*), demonstrating that the bulk of archaea density lies in between bacteria and eukaryotes. (*C*) Overhead view of (*B*), demonstrating that the bulk of eukaryote density is separated from both bacteria and archaea. Dashed cross-hairs represent the origin of the thermodynamic coordinate system, and the approximate positions of *Cyanobacteria* and *Thermotoga*, some of the most ancient organisms known, are near this origin, as indicated by a white star. In contrast, the approximate position of a more modern, biomass-abundant organism, trees, is indicated by a white cross.

In contrast to the pronounced differences revealed by TE frequency usage clustering, the clustering with respect to species (fig. 3, left side) yielded a complex tree topology that, at first glance, failed to group according to broad taxonomic distinctions, or any other obvious organizing principle. Although eukaryotes appeared to exhibit a slight enrichment in the moderately low stability TEs 3 and 4, inspection of the high-level branch points did not highlight major TE usage paradigms departing from the previously described pattern. Despite this, we noted a rich variety in the fine structure of TE usage that transcended species and domain boundaries, and a rough pattern of Gram-staining with cluster position was observed in bacteria (fig. 3, labels). We reasoned that this fine structure contained alternate information about TE usage not apparent through simple Euclidian distance metrics, and that performing a principal components analysis (PCA) on the complete TE usage matrix could reveal additional patterns coupled to this fine structure. As expected, the orthogonal basis eigenvectors of the PCA did not mirror the overall patterns of TE enrichment as observed above, instead describing a cryptic mixture of informative TE use. Eigenvalues indicated that the first two eigenvectors contained more than 90% of the information from the eight native state TEs (supplementary table S7, Supplementary Material online), thus permitting visualization of essentially all of the thermodynamic information in two dimensions (fig. 4).

### PCA Reveals Thermodynamic “Niches” of Kingdoms and Organisms

Surprisingly, PCA revealed a clear discrimination in TE usage patterns between bacterial, archaeal, and eukaryotic domains of life. Each domain was found to occupy contrasting sectors of divergent shapes, sizes, and scaled densities (fig. 4*A*). The predominantly unicellular bacteria and archaeal clades occupied a partially overlapping area, bacteria flanked by archaea in the PC2 dimension, whereas the more multicellular eukaryotes separated into a distinct space of their own (fig. 4*B*). Bacteria and eukaryotes are further identifiable by their oblate areas, inhabiting ellipsoid boundaries stretching lengthwise along the PC1 axis. However, although the bacterial density along the PC1 axis was fairly uniform, the eukaryotic density was largely focused in a limited area, with a greater spread of outliers defining the reaches of the ellipsoid boundary (fig. 4*B*). In contrast, archaea instead were symmetrically distributed, and notably positioned in partial intersection with both the bacterial and eukaryotic densities (fig. 4*B*).

**Fig. 5.**
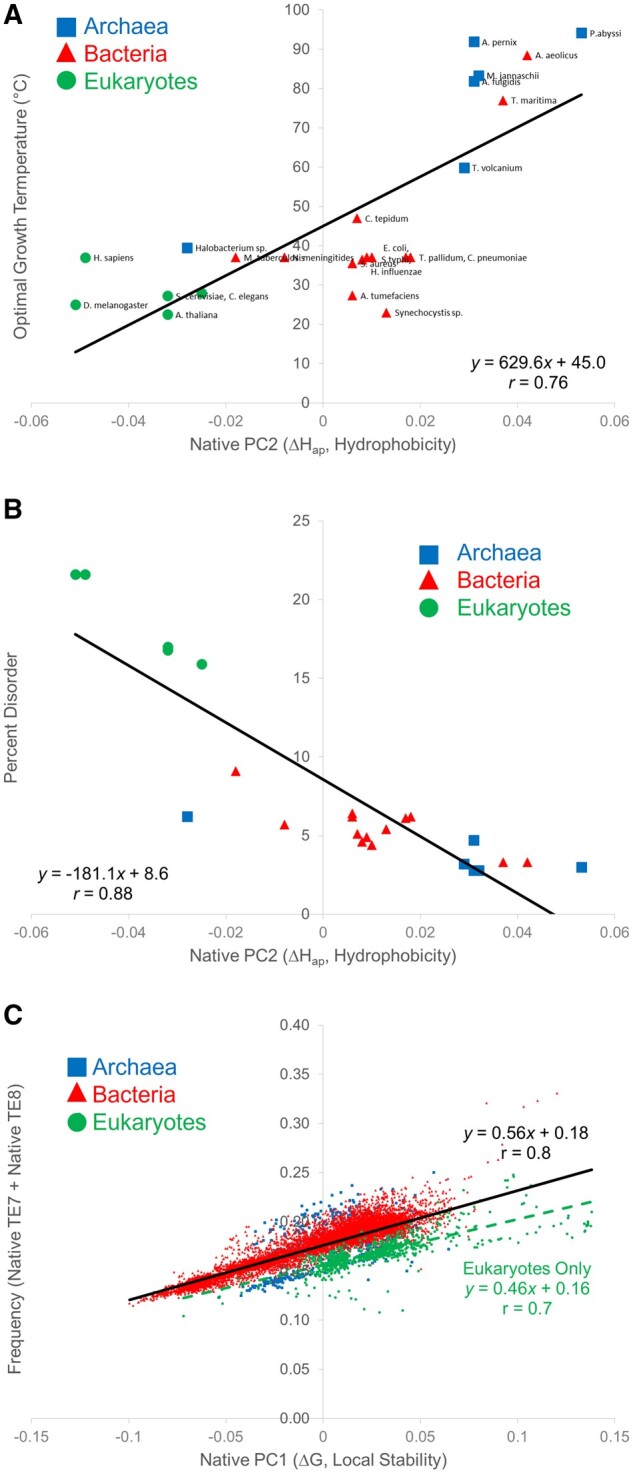
TEs predict organism characteristics. (*A*) Principal component 2 (PC2) predicts organism growth temperature. Optimal organism growth temperature and PC2 share a modest linear correlation (*P *<* *0.05). (*B*) PC2 predicts intrinsic disorder content of the proteome (*P *<* *0.01). (*C*) PC1 reflects the amount of the most stable residues of a proteome (*P *<* *10^−6^). Note that this quantity is distinct from the average stability of a proteome. Dashed green line indicates a significantly different slope when only eukaryotes are considered, suggesting that eukaryotes are a thermodynamically distinct kingdom in terms of proteome energetics.

We asked whether the distinctive domain geometries observed in the PCA analysis could be explained by known physical parameters. To this end, we explored whether the PC transformed data could be predicted by a library of growth temperatures for a variety of organisms (Materials and Methods). We found that the position of organisms along the PC2 axis correlated with both optimal growth temperature (fig. 5*A*) and intrinsic disorder content (fig. 5*B*) for a set of well-studied model organisms, and PC2 could be physically explained by native state apolar enthalpy (supplementary fig. S2*B*, Supplementary Material online), related to hydrophobicity (supplementary fig. S3, Supplementary Material online). PC1, which accounted for the largest information content (72%) of the PCA, and was clearly related to the amount of stable TEs in the proteome (fig. 5*C*;supplementary fig. S2*A*, Supplementary Material online). Notably, of the three kingdoms only eukaryotes exhibited a significantly different, weaker, slope of the trend in figure 5*C* (green dashed line).

Inspection of organisms contained under density peaks revealed interesting patterns, suggestive of “thermodynamic niches.” Gram-positive and negative bacteria clearly clustered under distinct peaks (fig. 4*A*), with *Actinobacteria* almost exclusively populating the peak with smallest values of PC1. Although fungi and halophilic archaea comprised the largest peaks of their respective kingdoms (fig. 4*A*), in general the height and location of density peaks appeared unrelated to estimates of organism abundance or biomass. For example, although trees plausibly account for the majority of biomass on Planet Earth (Bar-On et al. 2018), trees did not dominate any of the three eukaryotic density peaks (fig. 4*C*, white cross). Instead, uneven organism sampling within the proteome reference set probably obfuscated any relationship between thermodynamic niche and organism abundance. Obligate endosymbionts with reductive genomes, such as *Rickettsiales*, *Nasuia*, and *Phytoplasma*, populated the sparse bacterial points with largest values of PC1 and PC2 (fig. 4*B*, upper right). Parasites, such as trypanosomes and *Plasmodium*, populated the sparse eukaryotic points with large values of PC1 and small values of PC2 (fig. 4*B*, lower right), suggesting that other outlier points may harbor medically or evolutionarily interesting model organisms. On the other hand, cyanobacteria and *Thermotogales*, belonging to some of the earliest organismal lineages known on the basis of the fossil record (Berman-Frank et al. 2003; Di Giulio 2003; Dodd et al. 2017), were located near the origin of thermodynamic space (fig. 4*C*, white star). Although we do not propose a phylogenetic tree here, this last observation would be consistent with thermodynamic evolution of higher organisms radiating outward from the figure 4 origin rather than unidirectional thermodynamic evolution along a PC1/PC2 axis.

### Thermodynamic Properties of Individual Proteins: Secondary Structure and Global Stability

**Fig. 6.**
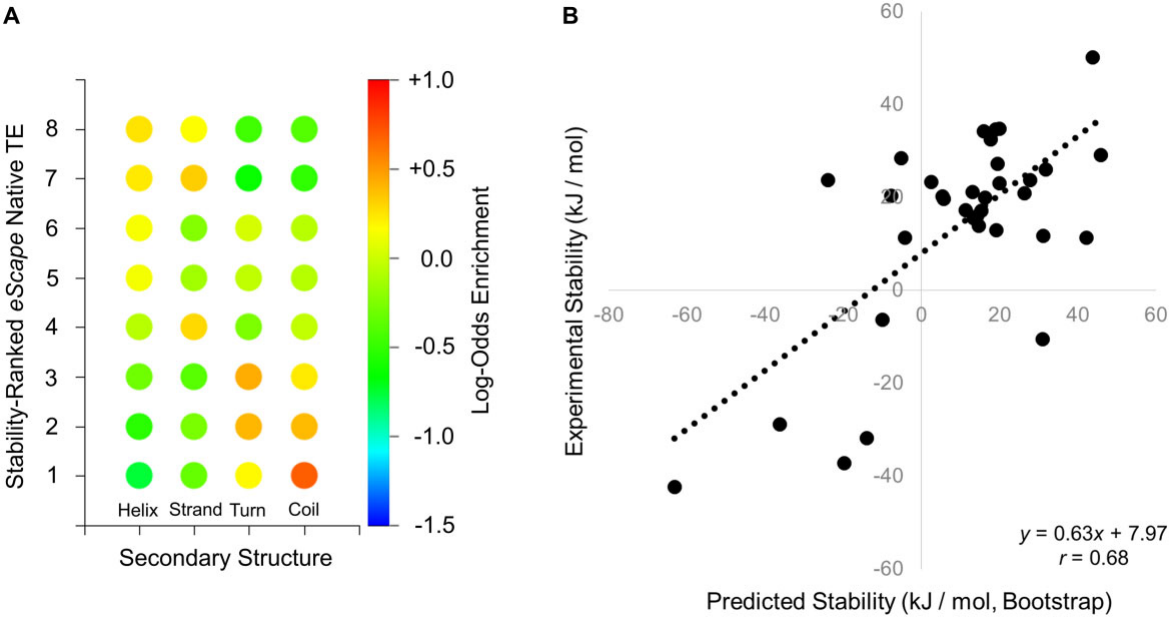
eScape TEs capture secondary structure (order/disorder) and stability information about individual proteins. (*A*) Native State TEs reflect the presence or absence of secondary structure in the primary sequence of 572,263 structured proteins. Red color indicates population enrichment and blue color indicates depletion relative to background, as described in Materials and Methods. Helix and strand are enriched in the most stable environments, whereas turn and coil are enriched in the least stable environments. (*B*) TEs approximate experimental two-state stability for a set of structured and intrinsically disordered proteins (Materials and Methods; equation [4]). Predictions were made using the average set of bootstrapped parameters (supplementary table S5, Supplementary Material online).

Of course, the proteome characteristics observed at the organism level were built from properties of individual proteins. Turning now to focus on these properties emphasizes the difference between TEs and traditional amino acid sequence analysis. First, secondary structure elements such as alpha helices or beta sheets cannot be used to predict TEs or their position-specific boundaries. Types of secondary structure found in folded proteins are only weakly correlated with specific TEs (fig. 6*A*), with helices and strands in particular both preferentially found in stable native state regions. Conversely, although structured regions might be distinguishable from coil and turn regions, TEs cannot be derived from secondary structure content alone. In contrast to the long-standing practical discovery that secondary structure propensities can be usefully predicted from amino acid sequence, TE sequence does not appear to be able to predict secondary structure.

What then are TEs able to predict? Previous work has established that TEs contain information on the conformational specificity of sequence for structure (Lattman and Rose 1993; Hoffmann et al. 2016). Extending this observation, we find here that TEs, although local reporters of the thermodynamic ensemble, can be also interpreted as weighted additive contributions to the experimental global stability of each protein. This interpretation leverages theoretical work from other laboratories (Ghosh and Dill 2009), treating the free energy as a sum of individual residue stabilizing enthalpic contributions, offset by a destabilizing conformational entropy term (equation [4]). The validity of this simple treatment is supported by a significantly effective ability to predict the measured stability of a globular protein solely from its TEs (fig. 6*B*). Additionally, equation (4) shows some ability to separate structured proteins from intrinsically disordered ones (supplementary fig. S1*C*, Supplementary Material online), as expected given that the types of environment correlate with presence or absence of structure, as already seen (fig. 6*A*). Correlations are anecdotally observed between the predicted stability and experimental melting temperatures of mesophilic and thermophilic variants within the same protein family, such as AK, cold-shock protein, and dihydrofolate reductase. All of the above suggest that TE statistics will be useful at the single protein level as well as at the whole-proteome level.

## Discussion

In this study, we explored the biological implications of a broad survey of position-specific TEs in proteins, which were derived from over 10,000 distinct proteomes, representing all three domains of life. This analysis is analogous to a structural characterization in that it reports on the TE at each position in a protein, as opposed to the energetic contribution of an amino acid. Importantly, these calculations do not represent a trivial, scaled-up sequence analysis. Rather, this work asks whether there are basic principles of physical organization in evolutionary biology, with the thermodynamic properties of proteins as the focus. Although the position-specific stabilities of proteins are subject to selective and neutral evolutionary forces over time, it is neither expected nor known whether living systems, as a whole, differentially exploit TEs as a mechanism for adaptation. Are there TE signatures that unify clades, despite the foundational physical realities of proteomic thermodynamics transcending phylogenetics? We consequently explored the overall statistics of TE occurrence to probe for biologically distinctive patterns.

The over-riding, if unconventional, pattern seen in the thermodynamic data is that bacteria and archaea, in general, cluster more closely together than do eukaryotes and archaea (fig. 4*B*).

State-of-the-art phylogenetic trees, built from primary sequence relationships, consistently reveal that eukaryotes are more closely related to archaea rather than bacteria, probably through transitional Asgard archaea (Eme et al. 2018; Doolittle 2020; Liu et al. 2021). Although some archaea occupy a thermodynamic border between bacteria and eukaryotes (fig. 4*B*), unexpectedly these organisms are not Asgard (supplementary fig. S4, Supplementary Material online), but turn out to largely be halophilic archaea (fig. 4*A*).

In fact, the thermodynamic data point to an evolutionary scenario whereby eukaryotes are energetically distinct from the other domains of life, perhaps due to their increased content of intrinsic disorder (fig. 5*B*, upper left) (Schlessinger et al. 2011). The thermodynamic separation of eukaryotes from the other domains is even more pronounced when the specific, locally unfolded denatured state is included in the analysis (supplementary fig. S4, lower right, Supplementary Material online). Although the concept of the tree-of-life is currently undergoing revision (Blais and Archibald 2021) due to, for example, horizontal gene transfer (Soucy et al. 2015; Doolittle and Brunet 2016) and an increased appreciation for network relationships among organisms (Puigbò et al. 2010), this eukaryotic separation from bacteria and archaea has also been noted in a tree constructed from the feature information of entire proteomes (Choi and Kim 2020) as well as in a tree constructed from protein fold co-occurrence (Kurland and Harish 2015). Toward reconciliation of these conflicting evolutionary scenarios, we and others posit that thermodynamic aspects of protein evolution are an important mechanism of organism adaptation (Ghosh et al. 2016; Trudeau et al. 2016; Saavedra et al. 2018), which to date have not commonly been represented in phylogenetic relationships. However, the results presented here suggest that this type of information could be a valuable addition to tree-building efforts.

Closer inspection of the thermodynamic data reveals an intriguing eukaryotic innovation: Why do eukaryotes exhibit higher intrinsic disorder content (fig. 5*B*) despite more abundant higher stability environments (fig. 5*C*)? Possibilities for this observation include; 1) the multidomain structure of many eukaryotic proteins, where locally stable domains are interspersed with disordered stretches, such that the average location in PC space reflects both properties; or 2) increased eukaryotic use of mechanisms to stabilize protein structure that do not rely on hydrophobicity, such as hydrogen bonds (Myers and Pace 1996; Pace et al. 2014), salt bridges (Bosshard et al. 2004), conformational entropy (Matthews et al. 1987; Pace et al. 1988; Nagibina et al. 2019), or covalent linkages (Fass 2012; Wensien et al. 2021). Related to the second point, the longer average lengths of eukaryotic proteins (Brocchieri and Karlin 2005) increase the temperature dependence of stability for globular proteins with a hydrophobic core, due to the curvature of the free energy of stability as a function of temperature that results from the larger heat capacity change ΔCp of unfolding larger proteins (Alexander et al. 1992; Robertson and Murphy 1997). Because the function of a globular protein depends on its folded population, which in turn depends on its stability, the longer lengths of folded eukaryotic proteins might have a functional limitation in being especially sensitive to temperature unfolding. Thus, eukaryotes may have circumvented this limitation by evolving protein-based regulatory mechanisms less dependent on stability at a fixed temperature, namely allosteric multidomain intrinsically disordered proteins (Hilser and Thompson 2007; Schlessinger et al. 2011). In other words, intrinsic disorder-mediated allostery, specifically featured in eukaryotic organisms, could permit better temperature adaptation to specific environmental niches by minimizing the temperature “denaturation catastrophe” (Ghosh et al. 2016) of key regulatory proteins. Examples of such disorder-mediated regulatory proteins have already been reported for the essential homeostatic enzyme AK (Saavedra et al. 2018) and the transcription factor glucocorticoid receptor (Li et al. 2012).

We note in contrast, that a large proportion of bacterial proteomes occupy PCA space with the lowest stability TEs (fig. 4*B*, left side). These organisms are almost exclusively Actinobacteria, such as *Arthrobacter*, *Cornyebacteria*, *Mycobacteria*, and *Streptomyces*. Bacteria, as a kingdom, occupy the widest range of PC1 while simultaneously occupying a rather narrow range of PC2. Because bacteria exclusively exhibit a weak positive slope of PC2 relative to PC1 (fig. 4*C*), one thermodynamic interpretation is that increased protein stability in this kingdom is gained by increasing the average hydrophobicity of the proteome. However, the resulting expectation that decreased PC1 (i.e. decreased protein stability) correlates with intrinsic disorder content is not supported by our analysis (fig. 5*B*). This apparent paradox is resolved with the testable hypothesis that eukaryotes have evolved a different type of disorder from bacteria that is not consistent with two-state unfolding of a globular protein. In other words, the bacterial proteome is more likely to contain disordered proteins that are merely destabilized versions of structured proteins, whereas eukaryotes are more likely to contain disordered nonfolding proteins, such as phase separating proteins, which are found throughout the cell but are predominant in the nucleus.

Our analysis also showed that TEs occurrence frequencies are nonuniform across proteomes in general, with median stability TEs preferred in proteomic composition about twice as often as low or high stability TEs. This observation in itself establishes a baseline expectation for the distribution of TEs that could be used to inform functional protein design. The monomodal distributional shape is somewhat surprising considering that, for example, proteomic amino acid frequencies tend to be more homogeneously distributed, and are differentially enriched in linkers versus domains (Brune et al. 2018). Though we emphasize again that TEs are semantically orthogonal to primary amino acid sequence, one might hypothesize that physicochemically related selective pressures could mold the TE frequency distribution into a shape similar to the flatter amino acid distribution. However, we observe the contrary. This not only reinforces the semantic independence of TEs as sequential TDs, but also emphasizes the opportunity to develop and use orthogonal TE organizing principles to drive effective protein design solutions.

One open possibility could be to design proteins using non-natural TE frequency distributions. Considering that natural global protein stability is often marginally stable, natural TE distributions may be constrained by epistatic evolutionary limitations but in actuality only represent a subset of the physically valid space (Taverna and Goldstein 2002). Devising functional sequences in the naturally unoccupied regions of TE distribution space could subsequently imbue proteins with unusual character. For example, it is known that multiple divergent protein structures can be validly mapped to a single shared TE sequence (Wrabl and Hilser 2010; Wrabl et al. 2019). Engineering dynamic interconversion between multiple highly diverse structures may be possible through use of non-natural TE frequency distributions.

Although the overall TE frequency distribution appeared to be shared universally across the tree of life, our analysis also revealed that subtle variation in TE usage patterns contained sufficient information to discriminate between bacteria, archaea, and eukaryotes. This phylogenetic separation reinforces the argument that the relative balance between position-specific protein energetics is itself a substrate for adaptive evolution. As a result, differing taxa appear to have co-opted distinct thermodynamic vocabularies or dialects, by analogy to natural language varieties which share features, but are distinguished by peculiarities that may not necessarily be functionally interchangeable (Searls 2013).

What physical forces or practical adaptations can account for trends in TE statistics? Although the suite of possible driving factors is vast, to some degree we expect that fundamental physical factors such as organismal growth temperatures will track with TE trends. We observe this is the case, corroborating a pattern previously appreciated in only a limited number of prokaryotic organisms (Gu and Hilser 2009). However, the majority of the variation remains ripe for quantitative exploration. Patterns in protein length that tend toward longer, multidomain eukaryotic proteins may also bias demands on site-specific thermodynamic character (Brocchieri and Karlin 2005). There is evidence linking protein stability to evolvability (Bloom et al. 2006; Tokuriki and Tawfik 2009). Could distributional breadth in proteomic TE compositions poise populations for adaptation to ecological niches? Can TE signatures be used to predict molecular evolutionary rates? We hope that the TE database presented here will serve as a foundational resource to aid insight into these and other significant questions.

## Conclusions

A unique database of residue-specific TEs information has been compiled for a large number of proteomes from the three kingdoms of life, enabled by a fast sequence-based predictor of protein energetics, *eScape*. Certain useful characteristics of individual proteins, such as secondary structure content and tertiary stability, are predictable from the TE information. Analysis of these data at the species level reveals that optimal growth temperature and intrinsic disorder content of individual organisms are strongly related to other energetic properties of the proteome, specifically the apolar enthalpy. Most intriguing is the observation that the thermodynamic properties of eukaryotic proteomes are quite different from those of archaea and bacteria, possibly calling into question the evolutionary relationships between the three kingdoms.

## Materials and Methods

A database of 10,520 *Uniprot* Reference Proteomes, which have been “selected among all proteomes to provide broad coverage of the tree of life” (uniprot.org/proteomes) were downloaded as source material for further analysis (1,184 eukaryotes, 440 archaea, 8,896 bacteria). The *eScape* software package (Gu and Hilser 2008) was deployed on this source material to analyze all protein primary sequences and return each sequence, relabeled as a series of eight native state (i.e. folded) and eight denatured state TEs (Larson and Hilser 2004; Wang et al. 2008). The nomenclature convention of the native and denatured TEs followed (Hoffmann et al. 2016), in which TE1 corresponded to the lowest mean stability (least negative Δ*G*) and TE8 corresponded to the highest mean stability (most negative Δ*G*) within each state. Raw proteome sequence data and TEs data are freely available at https://afc-science.github.io/thermo-env-atlas/ (last accessed January 18, 2022). It is important to note that the *eScape* denatured environments do not refer to the completely unfolded state of the protein (i.e. a conformation devoid of all structure). Rather, the denatured environments refer to a specific denatured state of the protein where the conformational entropy contribution is heavily weighted, so as to bias the ensemble toward states where only short regions of local structure (e.g. a few turns of helix) are populated (Wang et al. 2008).

TEs are defined as specific combinations of average stability, enthalpy (divided into apolar and polar contributions), and conformational entropy, observed for each residue of a protein. Although these quantities are accessible either experimentally, for example, from NMR hydrogen-exchange experiments, or computationally, for example, from all-atom molecular dynamics simulation, in the two decades elapsed since the initial report (Wrabl et al. 2001) most of what has been learned about TE’s has come from high-throughput ensemble-based modeling of proteins (Larson and Hilser 2004; Wang et al. 2008). One of the first insights gained from cluster analysis was that all globular proteins are composed of a surprisingly small number of distinct TEs (i.e. eight) (fig. 1*A*, colored regions). Moreover, the original high-dimensional thermodynamic space (fig. 1*A*, thin blue axes) could be decomposed into only two principal components (fig. 1*A*, dark axes), corresponding to solvent-exposed surface area exposure and atomic polarity, providing a physical interpretation of how the statistical–mechanical thermodynamic properties of the protein ensemble are reflected by the reported energetics at a single residue position (Vertrees et al. 2009). One consequence of this low-dimensional organization is that the TEs can be roughly ranked according to the average local stability—TE1 is least stable and TE8 is most stable (fig. 1; supplementary table S1, Supplementary Material online).

Perhaps unexpectedly, many of the properties of a globular protein’s ensemble are in fact determined locally, permitting development of an effective sequence-based predictor of protein energetics named *eScape* (“energetic landscape”) (fig. 1*B*, top). As previously detailed (Gu and Hilser 2008), *eScape* is parameterized from the experimentally verified ensemble-based protein modeling algorithm developed in this laboratory (Hilser and Freire 1996) to understand hydrogen exchange (Liu et al. 2012), protein allostery and functional adaptation (Pan et al. 2000; Schrank et al. 2009; Saavedra et al. 2018), cold-denaturation of proteins (Babu et al. 2004), protein design (Wrabl et al. 2019), and thermodynamic fold recognition (Wrabl et al. 2002). The *eScape* algorithm is publicly available both as a web-service for individual proteins (http://best.bio.jhu.edu/eScape, last accessed January 18, 2022) and as a batch package from the authors upon request. Because sequence-based prediction of protein energetics is extremely fast (<1 s per amino acid sequence), *eScape* is the enabling technology permitting multiproteomic analysis. Although it is expected that, as a verified representation of the energetics of the protein ensemble, *eScape* high-stability regions would correspond with experimental regions of hydrogen exchange protection, this has not been formally checked to date, although *eScape* has been shown to agree with the structure-based calculation embodied in the COREX algorithm (Gu and Hilser 2008), and COREX has been shown to correlate with experimental protection factors (Liu et al. 2012).

In detail, the relabeling of each amino acid sequence in terms of TEs was accomplished as follows (fig. 1*B*). The *eScape* output for every amino acid *j* in the sequence was treated as two 4D vectors, one each for the native (N) and locally denatured (D) states, that is, {Δ*G_j_*^N^, Δ*H*_apolar,__*j*_^N^, Δ*H*_polar,_*_j_*^N^, *T*Δ*S*_conf,_*_j_*^N^} and {Δ*G_j_*^D^, Δ*H*_apolar,_*_j_*^D^, Δ*H*_polar,_*_j_*^D^, *T*Δ*S*_conf,__*j*_^D^}. The native and denatured TEs corresponding to such vectors were defined as the TEs whose cluster centers in high-dimensional space, over a large database of proteins, were closest in Manhattan distance (fig. 1*B*, dashed lines), according to equations (1) and (2).

(1) $${\mathrm{TE}}_{j}^{N}\equiv \;\underset{k}{\min}\left[\mathrm{abs}\left(\bar{{\Delta G}_{k}^{N}}-{\Delta G}_{j}^{N}\right)+\mathrm{abs}\left(\bar{{\Delta H}_{\mathrm{apolar},k}^{N}}-{\Delta H}_{\mathrm{apolar},j}^{N}\right)+\mathrm{abs}\left(\bar{{\Delta H}_{\mathrm{polar},k}^{N}}-{\Delta H}_{\mathrm{polar},j}^{N}\right)+3a\mathrm{bs}\left(\bar{{T\Delta S}_{\mathrm{conf},k}^{N}}-{T\Delta S}_{\mathrm{conf},j}^{N}\right)\right].$$

(2) $${\mathrm{TE}}_{j}^{D}\equiv \;\underset{k}{\min}\left[\mathrm{abs}\left(\bar{{\Delta G}_{k}^{D}}-{\Delta G}_{j}^{D}\right)+\mathrm{abs}\left(\bar{{\Delta H}_{\mathrm{apolar},k}^{D}}-{\Delta H}_{\mathrm{apolar},j}^{D}\right)+\mathrm{abs}\left(\bar{{\Delta H}_{\mathrm{polar},k}^{D}}-{\Delta H}_{\mathrm{polar},j}^{D}\right)+\mathrm{abs}\left(\bar{{T\Delta S}_{\mathrm{conf},k}^{D}}-{T\Delta S}_{\mathrm{conf},j}^{D}\right)\right].$$

The index *k* runs over the eight native state TEs for equation (1), and the index *k* runs over the eight denatured state environments in equation (2). Average values $\left\{\bar{{\Delta G}_{k}^{N}},\;\bar{{\Delta H}_{\mathrm{apolar},k}^{N}},\bar{{\Delta H}_{\mathrm{polar},k}^{N}},\bar{{T\Delta S}_{\mathrm{conf},k}^{N}}\;\right\}$ and $\left\{\bar{{\Delta G}_{k}^{D}},\;\bar{{\Delta H}_{\mathrm{apolar},k}^{D}},\bar{{\Delta H}_{\mathrm{polar},k}^{D}},\bar{{T\Delta S}_{\mathrm{conf},k}^{D}}\;\right\}$ for each environment have been published (Wang et al. 2008; Hoffmann et al. 2016) and are given in supplementary tables S1 and S2, Supplementary Material online.

A total of 10,520 vectors of eight dimensions, whose entries are the proportion of native TEs, were calculated on a per-proteome basis as $T_{n}/(\sum_{n=1}^{8}T_{n})$, where *T* is the count of TEs and *n* is the environment number. UPGMA agglomerative hierarchical clustering with Euclidian distance was then used to examine these vectors. PCA was performed using the *sklearn* decomposition package on a matrix composed of the same vectors (Pedregosa et al. 2011).

Intrinsic disorder content was retrieved for 24 model organisms (Ward, McGuffin, et al. 2004; Ward, Sodhi, et al. 2004). Optimal growth temperatures for these same model organisms were collated from three large studies (Miralles 2010; Sauer et al. 2015; Engqvist 2018) plus Wikipedia (wikipedia.org), and the averages were used for analysis; these data are given in supplementary table S3, Supplementary Material online.

Primary sequence and experimental secondary structure data for 572,263 proteins, 96,019,709 residues, were retrieved from the Protein Data Bank (Berman et al. 2000) (rcsb.org) and the *develop275* release of the *ECOD* database (Cheng et al. 2014) (prodata.swmed.edu/ecod). These data were collated with *eScape* TE data for the same proteins computed as described above, such that every residue of every protein was assigned both a secondary structure type (helix, strand, turn, coil) and a native and denatured state TE. This set was necessarily a substantial subset of the entire database, as it was restricted to those *ECOD* domains containing no breaks in primary sequence. Log-odds scores reflecting enrichment or depletion of thermodynamics, given a secondary structure type, were computed according to equation (3) and the results displayed in figure 6*A*.

(3) $$\mathrm{Log}-\mathrm{Odds}\;\mathrm{Score}=\ln\frac{P_{j|k}}{P_{k}}=\ln\frac{N_{j|k}/N_{j}}{N_{k}/N}.$$

In equation (3), *N* is the total number of residue positions analyzed (i.e. 96,019,709 residues), *N_k_* is the number of residue positions with TE type *k*, *N_j_* is the number of residue positions with secondary structure type *j*, *N_j|k_* is the conditional number of residue positions of secondary structure type *j* given TE type *k*, *P_j|k_* is thus the conditional probability of finding secondary structure type *j* given TE type *k*, and *P_k_* is the probability of finding TE type *k* in the database. Index *j* runs 1 through 4 *DSSP* (Kabsch and Sander 1983) defined secondary structure types of helix (H, G, I), strand (E, B), turn (T, S), and coil (anything else) as reported by the Protein Data Bank. Index *k* runs 1 through 8 native state TE. Thus, for the native state data in figure 6*A*, equation (3) was evaluated separately for 4 × 8 = 32 categories of secondary structure and TE.

For the results in figure 6*B*, experimental data for 27 globular proteins was taken from Maxwell et al. (2005), where three proteins without structures given in table 2 of that work were omitted from analysis (i.e. their experimental amino acid sequences could not be inferred). This set was augmented with the following globular and intrinsically disordered protein experimental data: wild-type staphylococcal nuclease (Shortle and Meeker 1986), EXG:CBM (Hojgaard et al. 2016), human glucocorticoid receptor NTD isoforms A, C2, C3 (Li et al. 2012), P-protein (Chang and Oas 2010), alpha-synuclein (Moosa et al. 2015), and RCAM-T1 (Pace et al. 1988). The complete set used is given in supplementary table S4, Supplementary Material online. Leave-one-out bootstrapping was performed on the *eScape* native and denatured TEs of these 35 proteins in order to determine weights for a stability prediction expression as below:

(4) $$\Delta G=\sum_{i=1}^{8}w_{i}{\mathrm{NTE}}_{i}+\sum_{j=1}^{8}w_{j}{\mathrm{DTE}}_{j}-\mathrm{LRT}\ln Z.$$

In equation (4), Δ*G* is the experimental stability in kJ/mol under standard conditions of 100 mM salt, pH 7, 25 °C, *w_i_* and *w_j_* are optimized weights for NTE_*i*_ and DTE_*j*_, the number of native and denatured state environments, respectively, in the protein of type *i* or type *j*, where indices *i* and *j* run from 1 through 8 as described. *L* is the chain length of the protein in residues, *R* is the gas constant, *T* is the temperature (fixed at 25 °C), and *Z* is an adjustable parameter. The first two terms of equation (4) could be thought of as a solvation free energy and the last term could be thought of as a conformational entropy term applied uniformly to every residue, where *Z* is an estimate of the number of unfolded state conformations available to the backbone and side chain (Ghosh and Dill 2009). The *NMinimize* function of *Mathematica12* (Wolfram) was used in the bootstrapping to estimate optimal parameters for *w_i_*, *w_j_*, and *Z*, given in supplementary figure S1*C* and table S5, Supplementary Material online. In particular, the optimized value of *Z* turned out to be a reasonable value of approximately 20 unfolded state conformations per residue, depending on if the average values for the 35 left-out proteins, or the single value optimized over the full set, was used (supplementary table S5, Supplementary Material online).

To test the validity of equation (4), a set of 262 intrinsically disordered proteins and a length-matched set of 262 globular proteins of known structure were used. The intrinsically disordered proteins were taken from the *DisProt* database (Hatos et al. 2020) and restricted to lengths 50–400 (the approximate lengths used in the parameterization of equation [4]). The structured proteins were randomly chosen from the *ECOD* database mentioned above, such that each *DisProt* protein was matched with a structured protein of identical length and that no protein in the training set was used in this testing. These 524 proteins are given in supplementary table S6, Supplementary Material online. Stabilities of these proteins were predicted with equation (4) and the results shown in supplementary figure S1*C*, Supplementary Material online.

## Supplementary Material

Supplementary data are available at *Molecular Biology and Evolution* online.

## Supplementary Material

msac010_Supplementary_Data
