# Supplementary material for: A Thermodynamic Atlas of Proteomes Reveals Energetic Innovation across the Tree of Life
Source: Mol Biol Evol. 2022 Jan 17;39(3):msac010. doi: 10.1093/molbev/msac010 (PMC8896757; doi:10.1093/molbev/msac010)
Supplement: msac010_Supplementary_Data [file msac010_supplementary_data.pdf]

Supplementary Material: A thermodynamic atlas of proteomes reveals energetic innovation across the tree of life

Alexander F. Chin[1, †], James O. Wrabl[1], Vincent J. Hilser[1,2 *]

[1] Department of Biology, Johns Hopkins University, 3400 North Charles Street, Baltimore, MD 21218. USA.

[†] Translational Tissue Engineering Center, Department of Biomedical Engineering and Wilmer Eye Institute, Johns Hopkins School of Medicine, Baltimore, MD, 21231. USA.

[2] T.C. Jenkins Department of Biophysics, Johns Hopkins University, 3400 North Charles Street, Baltimore, MD 21218. USA.

* Correspondence: Vincent J. Hilser, hilser@jhu.edu, tel. 410-516-6072

Background and Rationale of *eScape* and Thermodynamic Environments

Although for many years it has been dogmatic that the folded structure of a protein determines its function, a growing body of evidence demonstrates that the flexibility and local unfolding of structure also has important functional consequences (Berlow, et al. 2018). Leveraging an experimentally verified, all-atom structural-thermodynamic model of the statistical-mechanical protein ensemble *COREX*, (Hilser and Freire 1996; Liu, et al. 2012), *eScape* was developed to provide a fast, sequence-based estimate of the degree of local unfolding of different regions of a protein.

In detail, *eScape* was parameterized as follows (Gu and Hilser 2008). *COREX* ensembles for a database of more than one hundred diverse globular proteins were parsed into overlapping tri-peptides and classified by the central residue of each tri-peptide into twenty distinct sets. Each set for each amino acid thus consisted of observed statistics from natural proteins of the 400 possible triplets. The minimum and maximum thermodynamic values of stability, enthalpy, and entropy (*i.e.* ΔG, ΔH_{apolar}, ΔH_{polar}, and $T\Delta S_{conformational}$) were tabulated for each type of triplet. From these minimum and maximum values, a bootstrapped four-dimensional linear prediction equation was developed using machine learning, such that the expected thermodynamics for any amino acid sequence could be interpolated from the prediction equation.

In separate but related work, it was discovered that the *COREX* ensembles contained information about the most likely amino acid to occur at that position in the folded structure (Wrabl, et al. 2001, 2002). Thus this parallel development also linked amino acid sequence to thermodynamics, but in a different way than in the *eScape* parameterization. A coarse-grained binning was developed to quantify the likelihood of each of the twenty amino acids to be in stable or unstable regions of structure, and this scheme of binning was named "thermodynamic environments". Simple dynamic programming algorithms demonstrated that the log-odds statistics of thermodynamic environments alone were sufficient to match amino acid sequence to a thermodynamic representation of structure, remarkably performing fold recognition without structural knowledge of the fold (Larson and Hilser 2004; Wang, et al. 2008).

This current manuscript couples the *eScape* predictions to the thermodynamic environments, potentially linking protein stability at the level of individual proteins with organismal evolution at the level of entire proteomes, with the only input being amino acid sequence information.

Table S1. Average Native State Thermodynamic Environments (Cluster Centers) (Hoffmann, et al. 2016).

Native	1	2	3	4	5	6	7	8
N ΔG	-3.5 *	-4.4	-6.4	-7.5	-8.5	-9.8	-10.5	-12.4
N ΔH_{apolar}	4.9	6.3	6.5	10.9	8.8	9.1	14.0	14.2
N ΔH_{polar}	-6.1	-8.6	-11.5	-9.4	-12.4	-15.1	-12.5	-16.3
N $T\Delta S_{conf}$	-3.1	-4.1	-4.9	-4.1	-4.4	-5.2	-4.6	-5.6

* Values are in kcal/mol at 25.0 C.

Table S2. Average Denatured State Thermodynamic Environments (Cluster Centers) (Wang, et al. 2008).

Denatured	1	2	3	4	5	6	7	8
D ΔG	10.7 *	10.2	9.8	9.1	9.1	8.4	7.6	7.5
D ΔH_{apolar}	-1.5	-1.5	-1.0	0.7	-1.0	-1.1	-0.3	-1.1
D ΔH_{polar}	1.1	2.4	-1.1	-0.4	0.0	1.1	-2.2	-0.9
D $T\Delta S_{conf}$	-9.9	-8.4	-10.9	-10.3	-9.3	-7.7	-9.8	-8.3

* Values are in kcal/mol at 25.0 C.

Table S3. Optimal Growth Temperature (°C) and Intrinsic Disorder Data for Model Organisms

Kingdom	Organism	ID % (Ward, et al. 2004)	OGT (Engqvist 2018)	OGT (Miralles 2010)	OGT (Sauer, et al. 2015)	OGT Wikipedia	Mean OGT	Stdev OGT	Native PC1	Native PC2
Archaea	Aeropyrum Pernix	4.7	90.0	95.0	90.8		91.9	2.7	-0.034	0.031
Archaea	Archaeoglobus fulgidis	2.8	81.0	83.0	81.5		81.8	1.0	0.010	0.031
Archaea	Halobacterium sp.	6.2	37.0	42.0			39.5	3.5	-0.021	-0.028
Archaea	Methanococcus jannaschi	2.8	80.0	85.0	85.0		83.3	2.9	0.021	0.032
Archaea	Pyrococcus Abyssi	3.0	90.0	96.0	96.5		94.2	3.6	-0.001	0.053
Archaea	Thermoplasma volcanium	3.2	60.0	60.0	59.6		59.9	0.2	0.022	0.029
Bacteria	Agrobacterium tumefaciens C58	6.4	28.0	28.0	26.0		27.3	1.2	-0.022	0.006
Bacteria	Aquifex aeolicus VF5	3.3	85.0	85.0	95.5		88.5	6.1	0.018	0.042
Bacteria	Chlamydophila pneumoniae AR39	6.2	37.0		37.0		37.0	0.0	0.020	0.018
Bacteria	Chlorobium tepidum TLS	5.1	45.0	48.0	48.0		47.0	1.7	0.000	0.007
Bacteria	Escherichia coli K12	4.6	36.0	37.0	36.4		36.5	0.5	0.003	0.008
Bacteria	Haemophilus influenzae Rd	4.4	37.0	37.0	37.0		37.0	0.0	0.020	0.010
Bacteria	Mycobacterium tuberculosis H37Rv	9.1	37.0		37.0		37.0	0.0	-0.070	-0.018
Bacteria	Neisseria meningitides MC58	5.7	37.0		37.0		37.0	0.0	0.003	-0.008
Bacteria	Salmonella Typhi	4.9	37.0	37.0	37.0		37.0	0.0	0.002	0.009
Bacteria	Staphylococcus aureus	6.2	37.0	33.5	36.0		35.5	1.8	0.034	0.006
Bacteria	Synechocystis species PCC 6803	5.4	23.0				23.0	0.0	-0.003	0.013
Bacteria	Thermotoga maritime	3.3	75.0	80.0	76.0		77.0	2.6	0.018	0.037
Bacteria	Treponema pallidum	6.1	37.0	37.0	37.0		37.0	0.0	0.014	0.017
Eukaryota	Arabidopsis Thaliana	16.8				22.5	22.5	0.0	0.036	-0.032
Eukaryota	Caenorhabditis elegans	15.9				28.0	28.0	0.0	0.046	-0.025
Eukaryota	Drosophila melanogaster	21.6				25.0	25.0	0.0	0.035	-0.051
Eukaryota	Homo Sapiens	21.6				37.0	37.0	0.0	0.024	-0.049
Eukaryota	Saccharomyces Cerevisiae	17.0	28.0		25.7	28.0	27.2	1.3	0.050	-0.032

Table S4. Experimental Protein Stability Data. The first 28 proteins correspond to Protein Data Bank entries, as described in Methods, Main Text. Reference data for experimental stabilities (ΔG_{exp}) are cited in Methods, Main Text; the units are kJ/mol. NTE denotes the number of *eScape* native state thermodynamic environments 1-8 in each protein, as defined by Equation (1), Main Text. DTE denotes the number of *eScape* denatured state thermodynamic environments 1-8 in each protein, as defined by Equation (2), Main Text. (The sum of these NTE or DTE counts equals the length of the protein *L* in residues.)

Protein	*L*	ΔG_{exp}	Error	NTE1	NTE2	NTE3	NTE4	NTE5	NTE6	NTE7	NTE8	DTE1	DTE2	DTE3	DTE4	DTE5	DTE6	DTE7	DTE8
1aps	98	20.4	0.2	1	6	26	12	37	10	6	0	20	2	11	4	27	14	10	10
1avz	57	20.3	1.4	2	6	9	10	14	8	7	1	6	3	14	8	15	1	8	2
1ayi	86	11.7	0.6	0	15	4	21	22	7	13	4	12	8	16	6	35	4	4	1
1divC	92	27.2	0.3	1	7	13	22	39	5	5	0	13	11	14	0	34	7	13	0
1divN	56	17.3	0.2	0	11	18	5	13	6	3	0	7	0	14	1	32	0	1	1
1e65	128	29.2	1.5	1	12	27	16	47	13	9	3	26	11	20	8	54	3	1	5
1fkf	107	23.4	0.9	0	20	19	26	16	9	17	0	16	25	15	5	32	4	6	4
1imq	86	20.9	0.6	0	6	23	15	23	4	12	3	11	11	15	9	29	2	7	2
1jo8	58	13.0	0.8	0	1	14	10	19	6	8	0	11	0	21	9	17	0	0	0
1k0s	151	50.2	0.6	1	12	8	56	38	4	30	2	28	32	17	4	45	25	0	0
1l8w	296	23.7	0.7	2	41	67	64	94	21	2	5	39	13	67	12	121	22	8	14
1lmb	87	21.2	1.9	1	10	14	14	35	3	6	4	3	1	12	12	36	13	2	8
1m9s	76	15.5	0.3	0	5	18	12	33	3	5	0	20	6	4	0	33	2	4	7
1n88	96	11.3	1.1	0	7	20	30	24	1	14	0	13	10	7	2	44	15	3	2
1nti	86	23.7	0.7	0	2	18	16	22	15	8	5	15	5	18	13	31	0	3	1
1o6x	81	17.1	0.4	0	10	7	19	21	11	12	1	9	4	3	4	35	6	10	10
1rfa	78	26.0	3.0	0	6	7	7	27	16	15	0	3	4	12	6	26	12	7	8
1ris	97	34.7	0.4	0	3	10	16	27	9	25	7	9	9	8	6	22	19	7	17
1rlq	56	15.9	2.5	1	6	15	14	11	3	6	0	1	0	13	10	28	1	0	3
1ryk	69	11.4	1.6	5	6	20	4	16	13	3	2	7	4	27	3	18	1	9	0
1shg	57	13.9	0.3	0	8	13	11	14	3	8	0	7	2	6	4	23	3	10	2
1spr	103	15.9	2.5	1	7	12	21	34	16	7	5	10	11	9	10	29	14	9	11
1ubq	76	34.2	0.4	0	5	20	19	22	1	9	0	8	6	11	1	35	11	4	0
1urn	96	34.8	1.5	3	7	3	18	24	15	21	5	0	2	8	12	37	15	1	21
2ci2	65	32.5	1.4	0	5	9	23	12	4	7	5	3	11	6	1	31	12	1	0
2ptl	78	19.9	0.9	0	5	18	16	12	15	10	2	12	4	18	12	30	0	0	2
3gb1	56	19.7	1.0	0	1	15	8	11	7	10	4	19	0	12	9	16	0	0	0
1ey0	149	23.1	2.0	0	9	48	23	42	9	14	4	19	13	35	16	46	10	2	8
EXG:CBM	110	28.5	1.0	5	22	18	11	45	5	4	0	35	4	12	0	41	4	6	8
hGRA-NTD	420	-42.3	2.1	10	81	83	74	106	28	31	7	51	14	66	25	133	33	40	58
hGRC2-NTD	331	-37.2	1.7	12	56	66	57	91	25	20	4	47	14	56	17	107	23	27	40
hGRC3-NTD	323	-31.8	1.3	10	57	60	56	91	25	20	4	41	13	56	17	107	22	27	40
P-protein	119	-10.5	0.8	2	8	25	11	27	21	22	3	3	3	13	4	47	28	8	13
α-synuclein	140	-28.9	4.2	3	35	34	28	25	8	2	5	48	5	28	12	40	3	3	1
RCAM-T1	104	-6.3	0.4	1	17	25	8	15	34	4	0	20	7	19	14	26	1	11	6

Table S5. Optimal Weights for Equation (4) from the bootstrapping calculation, and for the full set, of Table S4 proteins. Weights Nw1..Nw8 correspond to w_i and weights Dw1..Dw8 correspond to w_j in Main Text Equation (4). R is the gas constant and T is fixed at 25 °C (298K), thus Z corresponds to an average value of 18 +/- 2 denatured state conformations per residue. Values in rows 4 and below denote optimized parameters for the set when the named protein was removed, and the predicted stabilities in Main Text Figure 2b reflect this prediction for each protein using this individual set of leave-it-out bootstrapped parameters. Average and Standard Deviation rows were computed from rows 4 and below.

	Nw1	Nw2	Nw3	Nw4	Nw5	Nw6	Nw7	Nw8	Dw1	Dw2	Dw3	Dw4	Dw5	Dw6	Dw7	Dw8	RTlnZ
FullSet	2.8600E-06	0.0100	0.4269	0.4526	1.1710	0.9919	1.8171	1.6156	0.5686	1.5896	0.8479	1.0910	1.8988	0.8545	0.5865	0.0078	1.8362
Average	0.1230	0.0155	0.3710	0.4679	1.3066	0.9909	1.8857	1.3336	0.4537	1.5384	0.7616	1.0357	1.6344	0.5174	0.4052	0.0064	1.7065
Standard Deviation	0.1745	0.0241	0.3620	0.2835	0.1340	0.2540	0.2850	0.7562	0.3090	0.2603	0.3072	0.3233	0.3326	0.6196	0.5274	0.0214	0.3862
1aps	0.1935	0.0463	0.0513	0.3168	1.3244	0.8771	1.7029	1.3129	0.3328	1.7425	0.9175	1.0740	1.6304	0.1767	0.2706	0.0000	1.5831
1avz	0.1023	0.0097	0.0015	0.0309	1.2525	0.7329	1.1810	2.7295	0.2169	2.0300	0.3574	1.0342	1.1764	0.0548	0.1008	0.0038	1.2071
1ayi	0.8674	0.0013	0.0388	0.1667	1.2010	0.9292	1.6578	1.3554	0.2524	1.8700	0.8473	0.7573	1.6731	0.1771	0.0535	0.0017	1.4889
1divC	0.0886	0.0544	0.3888	0.4876	1.3104	1.0386	1.8366	1.5985	0.2937	1.4129	0.6324	0.7535	1.5296	0.4043	0.0267	0.0004	1.5840
1divN	0.0503	0.0036	1.0609	0.9688	1.3542	1.4093	2.1806	1.9212	1.0144	1.3253	1.0454	1.1267	2.1193	1.7370	1.4293	0.0064	2.4561
1e65	0.0263	0.0002	0.1031	0.0142	1.2177	0.9843	1.9203	2.5101	0.4374	1.8556	0.9857	0.4014	1.7870	0.2670	0.3828	0.0000	1.5979
1fkf	0.1184	0.0004	1.0594	0.9052	1.4802	1.5030	2.4508	1.6799	1.2341	1.0844	1.2949	1.1059	2.1430	2.0715	1.5159	0.0026	2.5972
1imq	0.0450	0.0089	0.0187	0.0177	1.1267	0.5313	1.6923	0.9471	0.3795	1.8752	0.5281	1.6131	1.5887	0.2981	0.3129	0.0041	1.4152
1jo8	0.0542	0.0842	0.2090	0.6288	1.4649	1.0443	1.9051	1.2458	0.3469	1.4690	0.8628	1.0683	1.5127	0.0853	0.2184	0.0026	1.6682
1k0s	0.0172	0.0000	0.3780	0.1988	0.9445	0.8787	1.5198	1.7187	0.1053	1.7516	0.4242	0.8348	1.8218	0.2713	0.0448	0.0011	1.4388
1l8w	0.0394	0.0028	0.0887	0.2632	1.4100	0.4936	2.3517	0.3986	0.6476	0.9849	0.1908	1.7183	1.0168	0.2456	0.1178	0.1244	1.3485
1lmb	0.0150	0.0030	0.3231	0.3697	1.2495	1.1333	1.9838	2.4979	0.4072	1.7368	1.0570	0.2070	1.6216	0.1808	0.5455	0.0000	1.6750
1m9s	0.0087	0.0094	0.9162	0.8226	1.2743	1.3756	2.2524	2.6951	0.9466	1.5048	1.3481	0.6002	2.0326	1.4330	1.5455	0.0052	2.3700
1n88	0.1565	0.0001	1.1351	0.8449	1.2712	1.4395	2.2957	1.6578	1.0998	1.1782	1.0312	1.1118	2.2237	2.0760	1.2870	0.0095	2.4837
1nti	0.0106	0.0006	0.2099	0.7007	1.5385	0.9389	1.7179	0.4621	0.3358	1.3604	0.6767	1.3000	1.3931	0.0347	0.1553	0.0002	1.5799
1o6x	0.5561	0.0900	0.2177	0.5345	1.4594	0.9713	1.7906	1.0975	0.3713	1.6732	0.9048	1.2777	1.6224	0.2454	0.3204	0.0000	1.7347
1rfa	0.0899	0.0103	0.3543	0.1272	1.2287	0.8579	2.3099	0.7191	0.4690	1.4265	0.7615	1.0573	1.5964	0.4828	0.1664	0.0017	1.5937
1ris	0.0019	0.0040	0.1544	0.2996	1.1770	0.7916	1.5301	0.8565	0.3220	1.7499	0.7475	1.1886	1.6672	0.4041	0.2343	0.0001	1.5295
1rlq	0.0039	0.0179	0.2371	0.5177	1.3469	0.9176	1.6552	0.7534	0.3723	1.5048	0.7821	1.2057	1.5084	0.4439	0.2290	0.0025	1.6029
1ryk	0.0001	0.0000	0.1814	0.1667	1.1082	0.7366	1.7604	1.0568	0.2921	1.5747	0.4328	1.1968	1.4501	0.3126	0.0002	0.0000	1.3533
1shg	0.1354	0.0004	1.1855	0.8170	1.3008	1.3309	2.2559	1.9685	1.0445	1.5448	1.1800	1.0514	2.1789	1.8337	1.5422	0.0002	2.5059
1spr	0.1807	0.0159	0.1697	0.4181	1.2552	0.8302	1.6707	0.6617	0.4405	1.6736	0.8777	1.3454	1.7632	0.4461	0.4160	0.0001	1.6821
1ubq	0.0009	0.0000	0.0703	0.5297	1.4721	0.8342	1.6204	0.7041	0.3648	1.5808	0.9138	1.3538	1.4309	0.1782	0.3592	0.0001	1.5890
1urn	0.0765	0.0155	0.9989	0.8938	1.3232	1.2462	2.0566	1.6326	0.9642	1.5581	1.1115	1.2088	2.1157	1.5772	1.5018	0.0056	2.4110
2ci2	0.0949	0.0219	0.2507	0.3891	1.2729	0.8136	1.9463	0.7595	0.2036	1.4602	0.4374	1.1688	1.2446	0.0038	0.0086	0.0033	1.3645
2ptl	0.4080	0.0233	0.7259	0.6595	1.5800	1.2027	2.2287	1.0601	0.4130	1.1414	0.3706	0.4413	1.1058	0.2780	0.0478	0.0002	1.5850
3gb1	0.0390	0.0010	0.1242	0.4819	1.2861	0.9627	1.5231	0.3336	0.1176	1.5970	0.5529	0.9082	1.4735	0.0105	0.0230	0.0040	1.3967
1ey0	0.0586	0.0018	0.0001	0.2805	1.2594	0.6579	1.8351	0.6622	0.3062	1.4265	0.3602	1.5080	1.2509	0.0016	0.0510	0.0001	1.2923
EXG:CBM	0.0792	0.0000	0.4730	0.6010	1.1931	0.9763	1.7014	0.6418	0.2157	1.3583	0.3722	1.1655	1.4803	0.4839	0.0000	0.0000	1.5105
hGRA-NTD	0.2110	0.0076	0.5748	0.5514	1.2672	1.3971	1.9935	2.2964	0.6702	1.6293	0.8540	1.0314	2.1061	0.8882	0.1932	0.0054	2.0535
hGRC2-NTD	0.0464	0.0039	0.1267	0.5840	1.4214	0.9641	1.6621	0.5808	0.2228	1.4486	0.7051	0.9892	1.3450	0.0842	0.0428	0.0007	1.4665
hGRC3-NTD	0.2088	0.0136	0.0013	0.0236	1.1956	0.6860	1.9505	3.0308	0.5624	2.1429	1.3262	0.8518	2.1499	0.3723	0.9393	0.0356	1.8579
P-protein	0.0766	0.0148	0.2656	0.2559	1.2223	1.0352	1.8997	1.8338	0.3024	1.6344	0.6382	0.6570	1.7266	0.1733	0.0364	0.0005	1.5458
α-synuclein	0.0187	0.0038	0.5503	0.8766	1.4494	1.1068	1.9587	0.7031	0.0797	1.1588	0.3832	1.0261	1.2468	0.1225	0.0219	0.0028	1.5375
RCAM-T1	0.2255	0.0717	0.3420	0.6315	1.4904	1.0525	2.0026	0.5915	0.0962	1.3780	0.7442	0.9098	1.4716	0.2544	0.0434	0.0003	1.6216

Table S6. Length-matched sets of structured (Cheng, et al. 2014) and intrinsically disordered (Hatos, et al. 2020) proteins.

<table>
<tr><th>ECOD Identifier</th><th>L</th><th>Primary Sequence</th></tr>
<tr><td>>001110837|e1jmbB1|150.1.1.12|B:1-50</td><td>50</td><td>XDYLRELLKLELQAIKQYREALEYVKLPVLAKILEDEEKHIEWLETILGX</td></tr>
<tr><td>>000088279|e1evr.6|367.1.1.1|H:1-29,G:1-21</td><td>50</td><td>FVNQHLCGSHLVEALYLVCGERGFFYTPKGIVEQCCTSICSLYQLENYCN</td></tr>
<tr><td>>000089980|e3sgbI1|379.1.1.4|I:7-56</td><td>50</td><td>DCSEYPKPACTLEYRPLCGSDNKTYGNKCNFCNAVVESNGTLTLSHFGKC</td></tr>
<tr><td>>001151218|e2zv4P26|3529.1.1.2|P:272-322</td><td>51</td><td>PITTLGPRHYCVILDPMGPDGKNQLGQKRVVKGEKSFFLQPGERLERGIQD</td></tr>
<tr><td>>001434344|e4v60U7|3529.1.1.2|U:272-322</td><td>51</td><td>PITTLGPRHYCVILDPMGPDGKNQLGQKRVVKGEKSFFLQPGERLERGIQD</td></tr>
<tr><td>>001434498|e4v60i7|3529.1.1.2|i:272-322</td><td>51</td><td>PITTLGPRHYCVILDPMGPDGKNQLGQKRVVKGEKSFFLQPGERLERGIQD</td></tr>
<tr><td>>001349273|e4w9fI1|4215.1.1.3|I:104-154</td><td>51</td><td>VYTLKERCLQVVRSLVKPENYRRLDIVRSLYEDLEDHPNVQKDLERLTQER</td></tr>
<tr><td>>000008457|e2p3uA1|389.1.1.9|A:1-51</td><td>51</td><td>KLCSLDNGDCDQFCHEEQNSVVCSCARGYTLADNGKACIPTGPYPCGKQTL</td></tr>
<tr><td>>001158389|e4bkfA8|389.1.1.16|A:288-338</td><td>51</td><td>CKIGYYKALSTDATCAKCPPHSYSVWEGATSCTCDRGFFRADNDAASMPCT</td></tr>
<tr><td>>001317872|e4tpeU1|3666.1.1.1|U:1-51</td><td>51</td><td>IKVRENEPFDVALRRFKRSCEKAGVLAEVRRREFYEKPTTERKRAKASAVK</td></tr>
<tr><td>>001431190|e4v57AU1|3666.1.1.1|AU:3-53</td><td>51</td><td>IKVRENEPFDVALRRFKRSCEKAGVLAEVRRREFYEKPTTERKRAKASAVK</td></tr>
<tr><td>>000026637|e1knaA1|4.8.1.3|A:16-67</td><td>52</td><td>EYAVEKIIDRRVRKGMVEYYLKWKGYPETENTWEPENNLDCQDLIQQYEASR</td></tr>
<tr><td>>000988111|e2lvpC1|103.1.1.2|C:1-52</td><td>52</td><td>SNSQLNAMAHQIQEMFPQVPYHLVLQDLQLTRSVEITTDNILEGRIQVPFPT</td></tr>
<tr><td>>000057660|e2vwkA2|4970.1.1.16|A:447-500</td><td>54</td><td>GFIPSLLGDLLEERQKVKKKMKATIDPIEKKLLDYRQRAIKILANSFYGYYGYA</td></tr>
<tr><td>>000068950|e1knfA1|211.1.1.23|A:1-54</td><td>54</td><td>SIRSLGYMGFAVSDVAAWRSFLTQKLGLMEAGTTDNGDLFRIDSRAWRIAVQQG</td></tr>
<tr><td>>001137331|e2a69D3|325.1.7.19|D:395-448</td><td>54</td><td>VVKARVYPFEDDVEVSTGDRVAPGDVLADGGKVKSDVYGRVEVDLVRNVVRVVE</td></tr>
<tr><td>>001514730|e5aka11|375.1.1.21|1:1-54</td><td>54</td><td>AKGIREKIKLVSSAGTGHFYTTTKNKRTKPEKLELKKFDPVVRQHVIYKEAKIK</td></tr>
<tr><td>>000333333|e2pvxD1|375.1.1.31|D:1-54</td><td>54</td><td>MKKWVCTVCGYIYDEDAGDPDNGISPGTKFEELPDDWVCPLCGVGKDQFEKLED</td></tr>
<tr><td>>001150928|e2zuoM22|3529.1.1.1|M:1-55</td><td>55</td><td>MATEEAIIRIPPYHYIHVLDQNSNVSRVEVGPKTYIRQDNERVLFAPVRMVTVPP</td></tr>
<tr><td>>001429211|e4v4hAR1|101.1.1.53|AR:20-74</td><td>55</td><td>EIDYKDIATLKNYITESGKIVPSRITGTRAKYQRQLARAIKRARYLSLLPYTDRH</td></tr>
<tr><td>>001507256|e3j7rR2|637.1.1.2|R:2-56</td><td>55</td><td>SMLRLQKRLASSVLRCGKKKVWLDPNETNEIANANSRQQIRKLIKDGLIIRKPVT</td></tr>
<tr><td>>000379473|e3kit51|375.1.1.1|5:2-56</td><td>55</td><td>AKHPVPKKKTSKARRDARRSHHALTPPTLVPCPECKAMKPPHTVCPECGYYAGRK</td></tr>
<tr><td>>000392481|e3mmyH1|3136.1.1.1|H:2-56</td><td>55</td><td>GTTIKFNPPTGTDTMVKAGVSTNISTKHQCITAMKEYESKSLEELRLEDYQANRK</td></tr>
<tr><td>>001439608|e4v7uD01|375.1.1.1|D0:1-56</td><td>56</td><td>AVQQNKPTRSKRGMRRSHDALTAVTSLSVDKTSGEKHLRHHITADGYYRGRKVIAK</td></tr>
<tr><td>>000060918|e1oczW1|5006.1.1.1|W:1-56</td><td>56</td><td>FENRVAEKQKLFQEDNGLPVHLKGGATDNILYRVTMTLCLGGTLYSLYCLGWASFP</td></tr>
<tr><td>>000025745|e1semB1|4.1.1.116|B:2-58</td><td>57</td><td>TKFVQALFDFNPQESGELAFKRGDVITLINKDDPNWWEGQLNNRRGIFPSNYVCPYN</td></tr>
<tr><td>>001290709|e4jz3A1|4.1.1.116|A:4-60</td><td>57</td><td>MTFVALYDYESRTETDLSFKKGERLQIVNNTEGDWWLAHSLTTGQTGYIPSNYVAPS</td></tr>
<tr><td>>000025732|e1semA1|4.1.1.116|A:1-58</td><td>58</td><td>ETKFVQALFDFNPQESGELAFKRGDVITLINKDDPNWWEGQLNNRRGIFPSNYVCPYN</td></tr>
<tr><td>>001444253|e4v96Bg2|79.1.1.19|Bg:2-61</td><td>60</td><td>ASIKKVYRGMKNGAETINDDLEAINSELTSGGNVVHKTGDETIAGKKTFTGNVEVNGSLT</td></tr>
<tr><td>>000235995|e3zn931|887.1.1.1|3:1-60</td><td>60</td><td>MPRLKVKLVKSPIGYPKDQKAALKALGLRRLQQERVLEDTPAIRGNVEKVAHLVRVEVVE</td></tr>
<tr><td>>001442104|e4v8jAN1|377.1.1.8|AN:2-61</td><td>60</td><td>ARKALIEKAKRTPKFKVRAYTRCVRCGRARSVYRFFGLCRICLRELAHKGQLPGVRKASW</td></tr>
<tr><td>>000362278|e2wg6C1|2.1.1.70|C:35-95</td><td>61</td><td>SAPLLVGVVSDILEDGRVVVKSSTGPKFVVNTSQYINEEELKPGARVALNQQTLAIVNVLP</td></tr>
<tr><td>>001263525|e3qsuB2|4.1.1.40|B:6-66</td><td>61</td><td>NIQDKALENFKANQTEVTVFFLNGFQMKGVIEEYDKYVVSLNSQGKQHLIYKHAISTYTVE</td></tr>
<tr><td>>001397004|e2zvfC2|137.1.1.6|C:4-64</td><td>61</td><td>AAIEAVEEMERLLREASSILRVEPAKLPKTVERFFEEWKDQRKEIERLKSVIADLWADILM</td></tr>
</table>

<table>
<tr><td>>000971461|e4f88B1|3376.1.1.1|B:1-61</td><td>61</td><td>MSKINVNVENVSGVQGFLFHTDGKESYGYRAFINGVEIGIKDIETVQGFQQIIPSINISKS</td></tr>
<tr><td>>001196975|e4mcuD2|3009.1.1.8|D:68-129</td><td>62</td><td>PLGKDLTQAWAVAIALGVEDKITAPMFEAVQKTQTVQSVADIRKVFVDAGVKGEDYDAAWNS</td></tr>
<tr><td>>001265250|e1t5eM5|5086.1.1.6|M:73-134</td><td>62</td><td>PATYEADYQSAQANLASTQEQAQRYKLLVADQAVSKQQYADANAAYLQSKAAVEQARINLRY</td></tr>
<tr><td>>000072673|e1mhhF1|221.3.1.1|F:1-62</td><td>62</td><td>EVTIKVNLIFADGKIQTAEFKGTFEEATAEAYRYAALLAKVNGEWTADLEDGGNHMNIKFAG</td></tr>
<tr><td>>001504000|e4yfkC6|275.1.1.7|C:227-234,C:289-342</td><td>62</td><td>KVIFEIRDVEYIAGKVVAKDYIDESTGELICAANMELSLDLLAKLSQSGHKRIETLFTNDLD</td></tr>
<tr><td>>000321654|e2e75C3|325.1.7.35|C:170-231</td><td>62</td><td>NVFTASATGTITKIAKEEDEYGNVKYQVSIQTDSGKTVVDTIPAGPELIVSEGQAVKAGEAL</td></tr>
<tr><td>>001144323|e4cg5B1|5027.1.1.2|B:7-68</td><td>62</td><td>FVEPSRQFVKDSIRLVKRCTKPDRKEFQKIAMATAIGFAIMGFIGFFVKLIHIPINNIIVGG</td></tr>
<tr><td>>000150874|e4fbyZ1|5044.1.1.1|Z:1-62</td><td>62</td><td>MTILFQLALAALVILSFVMVIGVPVAYASPQDWDRSKQLIFLGSGLWIALVLVVGVLNFFVV</td></tr>
<tr><td>>001440802|e4v87D41|4987.1.1.2|D4:1-63</td><td>63</td><td>MKEGIHPKLVPARIICGCGNVIETYSTKPEIYVEVCSKCHPFYTGQQRFVDTEGRVERFQRRY</td></tr>
<tr><td>>001348107|e4w23c1|2.1.1.45|c:5-68</td><td>64</td><td>RVQPIKLARVTKVLGRTGSQGQCTQVRVEFMDDTSRSIIRNVKGPVREGDVLTLLESEREARRL</td></tr>
<tr><td>>000047165|e3ccqI1|101.1.16.1|I:67-130</td><td>64</td><td>GVPPTAELIKDEAGFETGSGEPQEDFVADLSVDQVKQIAEQKHPDLLSYDLTNAAKEVVGTCTS</td></tr>
<tr><td>>001440005|e4v7yB81|4281.1.1.1|B8:2-65</td><td>64</td><td>PKMKTHKGAKKRVKITASGKVVAMKTGKRHLNWQKSGKEIRQKGRKFVLAKPEAERIKLLLPYE</td></tr>
<tr><td>>001437095|e4v6yAH1|4988.1.1.2|AH:67-130</td><td>64</td><td>QGKAVVESIQRVSRPGLRIYKRKDELPKVMAGLGIAVVSTSKGVMTDRAARQAGLGGEIICYVA</td></tr>
<tr><td>>001240951|e3b0xA11|102.1.1.14|A:89-153</td><td>65</td><td>PRGVLEVMEVPGVGPKTARLLYEGLGIDSLEKLKAALDRGDLTRLKGFGPKRAERIREGLALAQA</td></tr>
<tr><td>>000369781|e3e1cV1|320.2.1.2|V:2-66</td><td>65</td><td>SMQDPIADMLTRIRNGQAANKAAVTMPSSKLKVAIANVLKEEGFIEDFKVEGDTKPELELTLKYF</td></tr>
<tr><td>>000068131|e2dehA1|872.1.1.1|A:2-67</td><td>66</td><td>GKVYKKVELVGTSEEGLEAAIQAALARARKTLRHLDWFEVKEIRGTIGEAGVKEYQVVLEVGFRLE</td></tr>
<tr><td>>000085476|e2qbiW1|325.1.8.2|W:19-84</td><td>66</td><td>RLGVKRFGGESVLAGSIIVRQRGTKFHAGANVGCGRDHTLFAKADGKVKFEVKGPKNRKFISIEAE</td></tr>
<tr><td>>000072672|e1k52B1|221.3.1.1|B:6-72</td><td>67</td><td>HHAMEEVTIKANLIFANGSTQTAEFKGTFEKATSEAYAYADTLKKDNGEWTVDVADGGYTLNIKFAG</td></tr>
<tr><td>>000008197|e2b8tA1|377.1.1.41|A:150-216</td><td>67</td><td>TAICNECGAEATHSLRKIDGKHADYNDDIVKIGCQEFYSAVCRHHHKVPNRPYLNSNSEEFIKFFKN</td></tr>
<tr><td>>001391875|e4fxgB5|529.1.1.1|B:2-68</td><td>67</td><td>VNFQKAINEKLGQYASPTAKRCCQDGVTRLPMMRSCEQRAARVQQPDCREPFLSCCQFAESLRKKSR</td></tr>
<tr><td>>001446889|e4v9rAR1|101.1.1.53|AR:20-87</td><td>68</td><td>AKVKATLGEFDLRDYRNVEVLKRFLSETGKILPRRRTGLSAKEQRILAKTIKRARILGLLPFTEKLVR</td></tr>
<tr><td>>001407185|e3j8bM2|101.1.2.97|M:268-335</td><td>68</td><td>LLHEQNMAKMRLLTFMGMAVENKEISFDTMQQELQIGADDVEAFVIDAVRTKMVYCKIDQTQRKVVVS</td></tr>
<tr><td>>001404131|e4ul1r2|616.1.1.5|r:82-150</td><td>69</td><td>EIPEDLYYLIKKAVSVRKHLERNRKDKDAKFRLILIESRIHRLARYYRTVAVLPPNWKYESATASALVN</td></tr>
<tr><td>>000045131|e2uuaR1|101.1.1.53|R:19-88</td><td>70</td><td>KAKVKATLGEFDLRDYRNVEVLKRFLSETGKILPRRRTGLSAKEQRILAKTIKRARILGLLPFTEKLVRK</td></tr>
<tr><td>>000163013|e2dx1A2|4.1.1.116|A:56-126</td><td>71</td><td>LAINELISDGSVVCAEALWDHVTMDDQELGFKAGDVIEVMDATNREWWWGRVADGEGWFPASFVRLRVNQD</td></tr>
<tr><td>>001429382|e4v4ji2|4988.1.1.2|i:67-138</td><td>72</td><td>PRRQGPDPRPEQVIHHIRRISKPGRRVYVGVKEIPRVRRGLGIAILSTSKGVLTDREARKLGVGGELICEVW</td></tr>
<tr><td>>000026022|e2pukB1|4.1.1.30|B:1-73</td><td>73</td><td>MNVGDRVRVTSSVVVYHHPEHKKTAFDLQGMEGEVAAVLTEWQGRPISANLPVLVKFEQRFKAHFRPDEVTLI</td></tr>
<tr><td>>000025568|e1lojI1|4.1.1.40|I:10-82</td><td>73</td><td>NVQRPLDALGNSLNSPVIIKLKGDREFRGVLKSFDLHMNLVLNDAEELEDGEVTRRLGTVLIRGDNIVYISRG</td></tr>
<tr><td>>001065906|e4g12B1|101.1.1.62|B:24-96</td><td>73</td><td>SDRRFQLLAAAERLFAERGFLAVRLEDIGAAAGVSGPAIYRHFPNKESLLVELLVGVSARLLAGARDVTTRSA</td></tr>
<tr><td>>000355506|e2jziA1|108.1.1.51|A:1-75</td><td>75</td><td>ADQLTEEQIAEFKEAFSLFDKDGDGTITTKELGTVMRSLGQNPTEAELQDMINEVDADGNGTIDFPEFLTMMARK</td></tr>
<tr><td>>001177955|e1bc3A3|176.1.1.1|A:245-319</td><td>75</td><td>IPAYLAETLYYAMKGAGTDDHTLIRVIVSRSEIDLFNIRKEFRKNFATSLYSMIKGDTSGDYKKALLLLCGGEDD</td></tr>
<tr><td>>001226360|e4faoI2|382.1.1.1|I:11-85</td><td>75</td><td>PLVTCTCESPHCKGPTCRGAWCTVVLVREEGRHPQEHRGCGNLHRELCRGRPTEFVNHYCCDSHLCNHNVSLVLE</td></tr>
<tr><td>>001145842|e2ve7A2|3297.1.1.1|A:134-208</td><td>75</td><td>GEETEDGIMHNKLFLDYTIKCYESFMSGADSFDEMNAELQSKLKDLFNVDAFKLESLEAKNRALNEQIARLEQER</td></tr>
<tr><td>>000058437|e1g3wA3|183.1.1.1|A:65-140</td><td>76</td><td>TPTGRTLATAVMRKHRLAERLLTDIIGLDINKVHDEASRWEHVMSDEVERRLVKVLKDVSRSPFGNPIPGLDELGV</td></tr>
<tr><td>>001120704|e4jprA1|3782.1.1.2|A:20-95</td><td>76</td><td>YFQGTANLTTSLLGDLLDDVTSIRHAVLQNRAAIDFLLLAHGHGCEDVAGMCSFNLSDQSESIQKKFQLMKEHVNK</td></tr>
<tr><td>>000116355|e4pgtA2|2485.1.1.23|A:2-77</td><td>76</td><td>PPYTVVYFPVRGRCAALRMLLADQGQSWKEEVVTVETWQEGSLKASCLYGQLPKFQDGDLTLYQSNTILRHLGRTL</td></tr>
<tr><td>>001553062|e4v5eCQ1|2.1.1.44|CQ:2-78</td><td>77</td><td>PKKVLTGVVVSDKMQKTVTVLVERQFPHPLYGKVIKRSKKYLAHDPEEKYKLGDVVEIIESRPISKRKRFRVLRLVE</td></tr>
</table>

>001167704|e3j16B5|205.1.1.7|B:1-77	77	MSDKNSRIAIVSADKCKPKKCRQECKRSCPVVKTGKLCIEVTPTSKIAFISEILCIGCGICVKKCPFDAIQIINLPT
>001446354|e4v9oCX1|4232.1.1.1|CX:2-78	77	SRVCQVTGKRPVTGNNRSHALNATKRRFLPNLHSHRFWVESEKRFVTLRVSAKGMRVIDKKGIDTVLAELRARGEKY
>001409738|e1d0xA3|4.1.1.2|A:2-79	78	NPIHDRTSDYHKYLKVKQGDSDLFKLTVSDKRYIWYNPDPKERDSYECGEIVSETSDSFTFKTVDGQDRQVKKDDANQ
>000373262|e3hkrB2|2485.1.1.23|B:1-78	78	PPYTVVYFPVRGRCAALRMLLADQGQSWKEEVVTVETWQEGSLKASCLYGQLPKFQDGDLTLYQSNTILRHLGRTLGL
>001385093|e1yx2B3|1.1.8.2|B:286-365	80	RKLVGLEMIEKGIPRHGYEVFQNGKSVGKVTTGTQSPTLGKNVGLALIDSETSEIGTVVDVEIRKKLVKAKVVKTPFYKR
>000077053|e1wukL3|244.4.1.4|L:1-40,L:493-533	81	SSYSGPIVVDPVTRIEGHLRIEVEVENGKVKNAYSSSTLFDKNKLSPVEASLIGTPVADAKRPVEILRTVHSFDPCIACGV
>000426160|e3q6cN1|2.24.1.1|N:1-82	82	LLQKRVIVSNKREKVINDRRSRQQTVTPAGSEMRYEASFRPENGGLEVVFRLDAPQYHALSVGDRGMLSYKGTAFVAFTPDP
>001394306|e2o26X1|11.1.1.242|X:12-93	82	PPSIHPAQSELIVEAGDTLSLTCIDPDFVRWTFKTYFNEMVENKKNEWIQEKAEATRTGTYTCSNSNGLTSSIYVFVRDPAK
>001431890|e4v5eAE2|212.1.1.16|AE:74-155	82	GTIPHEIEVEFGASKIVLKPAAPGTGVIAGAVPRAILELAGVTDILTKELGSRNPINIAYATMEALRQLRTKADVERLRKGE
>001426996|e4u55S72|327.18.1.6|S7:105-186	82	SRQVQKRPRSRTLTAVHDKILEDLVFPTEIVGKRVRYLVGGNKIQKVLLDSKDVQQIDYKLESFQAVYNKLTGKQIVFEIPS
>001307595|e3cupB1|11.1.1.26|B:132-214	83	TEALNHHNTLVCSVTDFYPAKIKVRWFRNGQEETVGVSSTQLIRNGDWTFQVLVMLEMTPHQGEVYTCHVEHPSLKSPITVEW
>000049437|e1wlzD1|108.1.1.55|D:5-87	83	ATADRDILARLHKAVTSHYHAITQEFENFDTMKTNTISREEFRAICNRRVQILTDEQFDRLWNEMPVNAKGRLKYPDFLSRFS
>001008167|e1vukQ1|174.1.1.4|Q:149-231	83	SILDIRQGPKEPFRDYVDRFYKTLRAEQASQEVKNWMTETLLVQNANPDCKTILKALGPAATLEEMMTACQGVGGPGHKARVL
>001012179|e1vuuI1|174.1.1.4|I:149-231	83	SILDIRQGPKEPFRDYVDRFYKTLRAEQASQEVKNWMTETLLVQNANPDCKTILKALGPAATLEEMMTACQGVGGPGHKARVL
>001298404|e4m72B1|101.1.2.202|B:5-88	84	VSEAQARRAVADIFNSTLASSAIGAAWELGALDELRENGKLDVSDFAVRHDLHEPAVVGMFTALASVGIVRREGATVVVGPYFD
>001236320|e1vu8t4|174.1.1.4|t:148-231	84	TSILDIRQGPKEPFRDYVDRFYKTLRAEQASQEVKNWMTETLLVQNANPDCKTILKALGPAATLEEMMTACQGVGGPGHKARVL
>000039154|e1t83C2|11.1.1.310|C:87-171	85	HIGWLLLQAPRWVFKEEDPIHLRCHSWKNTALHKVTYLQNGKDRKYFHHNSDFHIPKATLKDSGSYFCRGLVGSKNVSSETVNIT
>001416997|e3j3q7M2|174.1.1.4|7M:147-231	85	PTSILDIRQGPKEPFRDYVDRFYKTLRAEQASQEVKNWMTETLLVQNANPDCKTILKALGPAATLEEMMTACQGVGGPGHKARVL
>001418919|e3j3y4j2|174.1.1.4|4j:147-231	85	PTSILDIRQGPKEPFRDYVDRFYKTLRAEQASQEVKNWMTETLLVQNANPDCKTILKALGPAATLEEMMTACQGVGGPGHKARVL
>001290631|e4bizC1|605.1.1.3|C:58-142	85	EFLAAGASFNQMVTALERMMTSQQRLLSDISEELRTPLTRLQLGTALLRRRSGESKELERIETEAQRLDSMINDLLVMSRNQQKN
>000259660|e3j39H1|318.1.1.1|H:1-85	85	MRTINSNQCVKIPKDIKASVKARVVTITGTRGTLKRSFKHLALDMYMPDKRTLKVEKWFGTKKELAAVRTVCSHIENMIKGVTFG
>000115831|e1yfhA2|2484.2.1.2|A:6-91	86	EMKRTTLDSPLGKLELSGCEQGLHEIKLLGKGTSAADAVEVPAPAAVLGGPEPLMQCTAWLNAYFHQPEAIEEFPVPALHHPVFQQ
>001125342|e4mnhB2|11.1.1.64|B:124-210	87	IQNPDPAVYQLRDSKSSDKSVCLFTDFDSQTNVSQSKDSDVYITDKCVLDMRSMDFKSNSAVAWSNKSDFACANAFNNSIIPEDTFF
>000039199|e3d87D3|11.1.1.310|D:1-87	87	IWELKKDVYVVELDWYPDAPGEMVVLTCDTPEEDGITWTLDQSSEVLGSGKTLTIQVKEFGDAGQYTCHKGGEVLSHSLLLLHKKED
>000414469|e2y0yO1|616.1.1.5|O:2-89	88	PITKEEKQKVIQEFARFPGDTGSTEVQVALLTLRINRLSEHLKVHKKDHHSHRGLLMMVGQRRRLLRYLQREDPERYRALIEKLGIRG
>000157280|e1yqwA1|4024.1.1.1|A:177-264	88	LFYGELVHDNCPRLPHFEASEFAPSFDSEEAKKGFCLYELGCKGPVTYNNCPKVLFNQVNWPVQAGHPCLGCSEPDFWDTMTPFYEQG
>000224559|e4j3oC1|11.1.1.140|C:117-205	89	PAKLALPPDQAAEKLRFRRSANSLTLINPTPYYLTVTELNAGTRVLENALVPPMGESTVKLPSDAGSNITYRTINDYGALTPKMTGVME
>000070496|e2vhnG1|318.1.1.1|G:82-170	89	FTKKLQLVGVGYRAAVKGNVINLSLGFSHPVDHQLPAGITAECPTQTEIVLKGADKQVIGQVAADLRAYRRPEPYKGKGVRYADEVVRT
>001331839|e2nvzB7|4961.1.1.3|B:528-616	89	PEGQACGLVKNLSLMSCISVGTDPMPIITFLSEWGMEPLEDYVPHQSPDATRVFVNGVWHGVHRNPARLMETLRTLRRKGDINPEVSMI
>001546687|e4wodA2|199.1.1.1|A:363-452	90	VMPVEPLTYLAKLLEGASIEKCYTRKYLTPEIIKKYDGKRTTHGATLAHMIRNGAYNNRSICPRTGEAECYSTFIDYLDPLICDYHGVKD
>000066556|e3bpdE1|304.59.1.1|E:3-92	90	LKGLRRLVLDVLKPHEPKTIVFALKLSELENVDGVNIHLSEIDQATENIKITILGNNLDYEQIKGVIEDMGGVIHSVDEVVAGKIIVESV
>001112099|e2m3tA1|72.1.1.1|A:1-91	91	GSKTGTKITFYEDKNFQGRRYDCDCDCADFHTYLSRCNSIKVEGGTWAVYERPNFAGYMYILPQGEYPEYQRWMGLNDRLSSCRAVHLPSG
>001432832|e4v5mBC2|2111.85.1.6|BC:69-160	92	LGKQVRVLAIAKGEKIKEAEEAGADYVGGEEIIQKILDGWMDFDAVVATPDVMGAVGSKLGRILGPRGLLPNPKAGTVGFNIGEIIREIKAG
>000090310|e2gooF1|382.1.1.1|F:8-99	92	TQECLFFNANWERDRTNQTGVEPCYGDKDKRRHCFATWKNISGSIEIVKQGCWLDDINCYDRTDCIEKKDSPEVYFCCCEGNMCNEKFSYFP
>000090573|e1pdgB1|385.1.1.8|B:9-100	92	EPAMIAECKTRTEVFEISRRLIDRTNANFLVWPPCVEVQRCSGCCNNRNVQCRPTQVQLRPVQVRKIEIVRKKPIFKKATVTLEDHLACKCE
>000369131|e3gsqA1|11.1.1.26|A:182-274	93	TDAPKTHMTHHAVSDHEATLRCWALSFYPAEITLTWQRDGEDQTQDTELVETRPAGDGTFQKWVAVVVPSGQEQRYTCHVQHEGLPKPLTLRW
>000401488|e3gslB1|7.1.1.2|B:4-97	94	MEYEEITLERGNSGLGFSIAGGTDNPHIGDDPSIFITKIIPGGAAAQDGRLRVNDSILFVNEVDVREVTHSAAVEALKEAGSIVRLYVMRRKPP

>001067141|e4kj1G1|318.1.1.1|G:83-177	95	FTKKLQLVGVGYRAAVKGNVINLSLGFSHPVDHQLPAGITAECPTQTEIVLKGADKQVIGQVAADLRAYRRPEPYKGKGVRYADEVVRTKEAKKK
>000025339|e1aonQ1|236.1.2.1|Q:1-96	96	MNIRPLHDRVIVKRKEVETKSAGGIVLTGSAAAKSTRGEVLAVGNGRILENGEVKPLDVKVGDIVIFNDGYGVKSEKIDNEEVLIMSESDILAIVE
>000086626|e2cjrC1|808.1.1.2|C:33-128	96	NVTQAFGRRGPEQTQGNFGDQDLIRQGTDYKHWPQIAQFAPSASAFFGMSRIGMEVTPSGTWLTYHGAIKLDDKDPQFKDNVILLNKHIDAYKTFP
>001262370|e2r1zB5|2111.68.1.6|B:1-96	96	PLVLIGSGLSSEQQKMLSELAVILKAKKYTEFDSTVTHVVVPGDAVQSTLKCMLGILNGCWILKFEWVKACLRRKVCEQEEKYEIPEGPRRSRLNR
>001401416|e4u95B6|304.28.1.12|B:660-709,B:798-844	97	DAMVFAFNLPAIVELGTATGFDFELIDQAGLGHEKLTQARNQLLAEAAKHMVPFSAFSSSRWEYGSPRLERYNGLPSMEILGQAAPGKSTGEAMELM
>001446235|e4v9nBZ1|240.1.1.1|BZ:91-187	97	DEPVEMYVPLRFVGTPAGVRAGGVLQEIHRDILVKVSPRNIPEFIEVDVSGLEIGDSLHASDLKLPPGVELAVSPEETIAAVVPPEDVEKLAEEAAA
>000152653|e4gs3A1|2.1.1.54|A:10-107	98	ENNTVTLVGKVFTPLEFSHELYGEKFFNFILEVPRLSETKDYLPITISNRLFEGMNLEVGTRVKIEGQLRSYNRKSPEEGKNKLILTVFARDISVVPE
>000965598|e4fp5E1|2.2.1.3|E:1-98	98	GASQFFKDNCNRTTASLVEGVELTKYISDINNNTDGMYVVSSTGGVWRISRAKDYPDNVMTAEMRKIAMAAVLAGMRVNMCASPASSPNVIWAIELEA
>001312499|e4a9gI2|7.1.1.2|I:330-427	98	TLDTSTSSSASAEMITPALEGATLSDGQLKDGGKGIKIDEVVKGSPAAQAGLQKDDVIIGVNRDRVNSIAEMRKVLAAKPAIIALQIVRGNESIYLLM
>000158905|e1x66A1|102.1.1.29|A:1-98	98	GSSGSSGPPNMTTNERRVIVPADPTLWTQEHVRQWLEWAIKEYSLMEIDTSFFQNMDGKELCKMNKEDFLRATTLYNTEVLLSHLSYLRESSSGPSSG
>000307141|e2hs1A1|1.1.1.4|A:1-99	99	PQITLWKRPLVTIKIGGQLKEALLDTGADDTIIEEMSLPGRWKPKMIGGIGGFIKVRQYDQIIIEIAGHKAIGTVLVGPTPVNIIGRNLLTQIGATLNF
>001498314|e4q1wA1|1.1.1.4|A:1-99	99	PQITLWKRPLVTIRIGGQLKEALLDTGADDTVLEEMNLPGKWKPKMIGGIGGFIKVRQYDQIPIEICGHKAIGTVLVGPTPVNIIGRNLMTQIGCTLNF
>001210450|e3ad8A7|1.1.8.2|A:865-963	99	REDRKHLVSVLPVDSSLRLAEGAALVAADAVASEGVTPMEGWVTHAYNSPALGRTFGLALIKNGRNRIGEVLKTPVDGQLVDVQVSDLVLFDPEGSRRD
>000038033|e2h26B1|11.1.1.26|B:1-99	99	IQRTPKIQVYSRHPAENGKSNFLNCYVSGFHPSDIEVDLLKNGERIEKVEHSDLSFSKDWSFYLLYYTEFTPTEKDEYACRVNHVTLSQPKIVKWDRDM
>000361310|e2wdnS1|2484.1.1.47|S:11-109	99	KFRVRNRIKRTGRLRLSVFRSLKHIYAQIIDDEKGVTLVSASSLALKLKGNKTEVARQVGRALAEKALALGIKQVAFDRGPYKYHGRVKALAEGAREGG
>001283446|e4orgA1|11.1.1.26|A:144-244	101	SASTKGPSVFPLAPSSKSTSGGTAALGCLVKDYFPEPVTVSWNSGALTSGVHTFPAVLQSSGLYSLSSVVTVPSSSLGTQTYICNVNHKPSNTKVDKRVEP
>001110102|e3j57F1|304.12.1.1|F:1-101	101	MRHYEIVFMVHPDQSEQVPGMIERYTAAITGAEGKIHRLEDWGRRQLAYPINKLHKAHYVLMNVEAPQEVIDELETTFRFNDAVIRSMVMRTKHAVTEASX
>001297558|e4qhmE2|11.1.1.26|E:122-223	102	ASTKGPSVFPLAPSSKSTSGGTAALGCLVKDYFPEPVTVSWNSGALTSGVHTFPAVLQSSGLYSLSSVVTVPSSSLGTQTYICNVNHKPSNTKVDKRVEPKS
>001205801|e2ys6A9|325.1.1.3|A:338-439	102	LELEWTDEAVLGVVLAAKGYPGAYERGAEIRGLDRISPDALLFHAGTKREGGAWYTNGGRVLLLAAKGETLAKAKEKAYEQLAAIDCDGLFYRRDIGRRAIE
>000990067|e3t5vD1|101.1.2.187|D:197-299	103	FFLQMHLTDIRFYALRALSHTLNKKHKPIPFIYLENMLLFNNRQEIIEFCNYYSIEIINGDAADLKTLQHYSHKLSETQPLKKTYLTCLERRLQKTTYKGLIN
>001523247|e2y7rB1|2111.17.1.8|B:80-182	103	HRYVCMFRKDHPSAKSPMSLKQFSELEHVGVVALNTGHGEVDGLLERAGIKRRMRLVVPHFIAIGPILHSTDLIATVPQRFAVRCEVPFGLTTSPHPAKLPDI
>001423668|e4tn8A1|2485.1.1.40|A:4-106	103	PIEVTDQNFDETLGQHPLVLVDFWAEWCAPCRMIAPILEEIAKEYEGKLLVAKLDVDENPKTAMRYRVMSIPTVILFKDGQPVEVLVGAQPKRNYQAKIEKHL
>001431610|e4v5bBC2|327.11.1.18|BC:1-104	104	GQKVHPNGIRLGIVKPWNSTWFANTKEFADNLDSDFKVRQYLTKELAKASVSRIVIERPAKSIRVTIHTARPGIVIGKKGEDVEKLRKVVADIAGVPAQINIAE
>000033466|e1jynB3|11.1.1.197|B:626-730	105	FFQFRLSGQTIEVTSEYLFRHSDNELLHWMVALDGKPLASGEVPLDVAPQGKQLIELPELPQPESAGQLWLTVRVVQPNATAWSEAGHISAWQQWRLAENLSVTL
>000408998|e3l7pD1|304.5.1.10|D:1-105	105	GSMKKIEAIIRSDKLEDLKAALVQSGFIKGMTISQVLGFGNQRGYTEYVRGQKITPTLLAKVKVEIVAHDAAVEEMITTISQAVKTGEVGDGKIFVSPVDEIVRI
>000142300|e3qijA2|197.1.1.2|A:99-204	106	PDPAQLTEDITRYYLCLQLRQDIVAGRLPCSFATLALLGSYTIQSELGDYDPELHGVDYVSDFKLAPNQTKELEEKVMELHKSYRSMTPAQADLEFLENAKKLSMY
>001521287|e4kd5D2|2111.17.1.25|D:87-192	106	NDLVLISSADSSVSGMKDLTTDKVKKIAVGEAESVPAGKYADEVLTNLNLKDKLKDKLVFAKDVKEVLAWVQSGNADVGFVYFSDTVNNDKIKVVEKTDEKTHSPI
>001313864|e4cxdo1|375.1.1.25|o:1-106	106	MVNVPKTRRTFCKKCGKHQPHKVTQYKKGKDSLYAQGKRRYDRKQSGYGGQTKPIFRKKAKTTKKIVLRLECVEPNCRSKRMLAIKRCKHFELGGDKKRKGQVIQF
>000397974|e3ncjL1|11.1.1.179|L:1-107	107	DIQMTQSPSSLSASVGDRVTITCRASQSIGLYLAWYQQKPGKAPKLLIYAASSLQSGVPSRFSGSGSGTDFTLTISSLQPEDFATYYCQQGNTLPYTFGQGTKVEIK
>001519697|e4bdoD2|2111.17.1.38|D:109-215	107	LGISILYRKGTPIDSADDLAKQTKIEYGAVEDGATMTFFKKSKISTYDKMWAFMSSRRQSVLVKSNEEGIQRVLTSDYAFLMESTTIEFVTQRNCNLTQIGGLIDSK
>000025987|e1oz3B1|4.1.1.42|B:10-117	108	WSWESYLEEQKAITAPVSLFQDSQAVTHNKNGFKLGMKLEGIDPQHPSMYFILTVAEVCGYRLRLHFDGYSECHDFWVNANSPDIHPAGWFEKTGHKLQPPKGYKEEE
>001515845|e4xwoV1|11.1.1.179|V:3-110	108	IQMTQSPSSLSASVGDRVTITCRASQSVSSAVAWYQQKPGKAPKLLIYSASSLYSGVPSRFSGSRSGTDFTLTISSLQPEDFATYYCQQYPYYSSLITFGQGTKVEIK
>000034748|e1nj9A1|11.1.1.179|A:1-110	110	QAVVTQESALTTSPGETVTLTCRSSTGTITSDNYANWVQEKPDHLFSGLIGVNNARPPGVPARFSGSLTGDKAVLTITGAQTEDEAIYFCALWYSNHWVFGGGTKLTVLG
>000392021|e3mgvD3|186.1.1.4|D:20-129	110	SDEVRKNLMDMFRDRQAFSEHTWKMLLSVCRSWAAWCKLNNRKWFPAEPEDVRDYLLYLQARGLAVKTIQQHLGQLNMLHRRSGLPRPSDSNAVSLVMRRIRKENVDAGE
>000232380|e4b1zM1|3192.1.1.1|M:6-115	110	CRKDSLAIKLSNRPSKRELEEKNILPRQTDEERLELRQQIGTKLTRRLSQRPTAEELEQRNILKPRNEQEEQEEKREIKRRLTRKLSQRPTVEELRERKILIRFSDYVEV
>001481553|e4whtV1|11.1.1.179|V:3-113	111	DIVLTQTTPTLSATIGQSVSISCRSSQSLLESDGNTYLNWLLQRPGQSPQLLIYSVSNLESGVPNRFSGSGSETDFTLKISGVEAEDLGVYYCMQTTHAPTFGAGTKLELK

<table>
<tr><td>>001187611|e3tveQ2|2484.1.1.47|Q:1-111</td><td>111</td><td>ARLTAYERRKFRVRNRIKRTGRLRLSVFRSLKHIYAQIIDDEKGVTLVSASSLALKLKGNKTEVARQVGRALAEKALALGIKQVAFDRGPYKYHGRVKALAEGAREGGLEF</td></tr>
<tr><td>>000339810|e3bkmL1|11.1.1.179|L:34-145</td><td>112</td><td>DVLMTQTPLSLPVNLGEQASISCRSSQSIVHSNGHTYLEWYLQRPGQSPKLLIYQVSTRFSGVPDRFSGSGSGTDFTLRISRVEAEDLGVYYCFQASLVPLTFGAGTKLELK</td></tr>
<tr><td>>000427290|e3t65A1|11.1.1.179|A:1-113</td><td>113</td><td>DIVMSQSPSSLAVSAGEKVTMSCKSSQSLLNSRTRKNYLAWYQQKPGQSPKLLIYWASTRESGVPDRFTGSGSGTDFTLTITSVQAEDLAVYYCKQSYNLRTFGGGTKLEIKR</td></tr>
<tr><td>>001519157|e4r3yJ1|325.1.4.1|J:1-113</td><td>113</td><td>MDAEGLALLLPPVTLAALVDSWLREDCPGLNYAALVSGAGPSQAALWAKSPGVLAGQPFFDAIFTQLNCQVSWFLPEGSKLVPVARVAEVRGPAHCLLLGERVALNTLARCSG</td></tr>
<tr><td>>001420864|e3j77S43|221.1.2.3|S4:2-116</td><td>115</td><td>ARGPKKHLKRLAAPHHWLLDKLSGCYAPRPSAGPHKLRESLPLIVFLRNRLKYALNGREVKAILMQRHVKVDGKVRTDTTYPAGFMDVITLDATNENFRLVYDVKGRFAVHRITD</td></tr>
<tr><td>>000973768|e4at6J1|11.1.1.179|J:1-116</td><td>116</td><td>QVQLQQPGPELVTPGASVKMSCTASGYSFSSYNIHWVKQTPGQGLEWIGVIYPGNGDTSYNQKFRDKATLTADKSSSTAYMQLSSLTSEDSAVYHCARGGSGLLAYWGQGTLVTVS</td></tr>
<tr><td>>001285835|e4p59H2|11.1.1.179|H:2-117</td><td>116</td><td>VQLVESGGGLVQPGGSLRLSCAASGFTFSSYAMSWVRQAPGKGLEWVSAINSQGKSTYYADSVKGRFTISRDNSKNTLYLQMNSLRAEDTAVYYCARWGDEGFDIWGQGTLVTVSS</td></tr>
<tr><td>>001296465|e4psnD2|3781.2.1.1|D:1-116</td><td>116</td><td>GAMLPTLRTGLVIAAGYADKVRRVLFAQLRDAIKSGELSNKDVAMAAGNLNRVLFELLVNKLKADKLDVVRIQIDYEVRDSQIQFDFSTLRVELWRRVPEEEIAPIVEDFARAAPR</td></tr>
<tr><td>>000274114|e1vowR1|143.2.1.1|R:1-117</td><td>117</td><td>PRAKTGIVRRRRHKKVLKRAKGFWGSRSKQYRNAFQTLLNAATYEYRDRRNKKRDFRRLWIQRINAGARLHGMNYSTFINGLKRANIDLNRKVLADIAAREPEAFKALVDASRNARQ</td></tr>
<tr><td>>001442149|e4v8jDR1|218.3.1.1|DR:2-118</td><td>117</td><td>RHLKSGRKLNRHSSHRLALYRNQAKSLLTHGRITTTVPKAKELRGFVDHLIHLAKRGDLHARRLVLRDLQDVKLVRKLFDEIAPRYRDRQGGYTRVLKLAERRRGDGAPLALVELVE</td></tr>
<tr><td>>000391368|e3f7vA1|11.1.1.179|A:1-118</td><td>118</td><td>QVQLQQPGAELVKPGASVKLSCKASGYTFTSDWIHWVKQRPGHGLEWIGEIIPSYGRANYNEKIQKKATLTADKSSSTAFMQLSSLTSEDSAVYYCARERGDGYFAVWGAGTTVTVSS</td></tr>
<tr><td>>000968404|e4fqvI1|11.1.1.179|I:1-118</td><td>118</td><td>QVQLVQSGAEVKKPGSSVKVSCKSSGGTSNNYAISWVRQAPGQGLDWMGGISPIFGSTAYAQKFQGRVTISADIFSNTAYMELNSLTSEDTAVYFCARHGNYYYYSGMDVWGQGTTVT</td></tr>
<tr><td>>001311700|e4c5yB2|65.1.1.38|B:3-63,B:382-438</td><td>118</td><td>DEAKVTIIYAGLLIPGDGEPLRNAALVISDKIIAFVGSEADIPKKYLRSTQSTHRVPVLMPQLREGYEADVIALEENPLEDIKVFQEPKAVTHVWKGGKLFKGPGIGPWGEDARNPFL</td></tr>
<tr><td>>000381248|e3kpbC1|282.1.1.1|C:1-119</td><td>119</td><td>TLVKDILSKPPITAHSNISIMEAAKILIKHNINHLPIVDEHGKLVGIITSWDIAKALAQNKKTIEEIMTRNVITAHEDEPVDHVAIKMSKYNISGVPVVDDYRRVVGIVTSEDISRLFG</td></tr>
<tr><td>>000353518|e3c2pB4|4970.1.1.19|B:337-456</td><td>120</td><td>TPEQKAALKAEQATEFYVHTPMVQFYETLGKDRILELMGAGTLNKELLNDNHAKSLEGKNRSVEDSYNQLFSVIEQVRAQSEDISTVPIHYAYNMTRVGRMQMLGKYNPQSAKLVREAIL</td></tr>
<tr><td>>001241888|e3fcpH4|218.1.1.3|H:3-122</td><td>120</td><td>LTATVEQIESWIVDVPTIRPHKLSMTTMGCQSLVIVRLTRSDGICGIGEATTIGGLSYGVESPEAISSAITHYLTPLLKGQPADNLNALTARMNGAIKGNTFAKSAIETALLDAQGKALG</td></tr>
<tr><td>>000089378|e2g9tB1|4136.1.1.1|B:9-128</td><td>120</td><td>ANSTVLSFCAFAVDPAKAYKDYLASGGQPITNCVKMLCTHTGTGQAITVTPEANMDQESFGGASCCLYCRCHIDHPNPKGFCDLKGKYVQIPTTCANDPVGFTLRNTVCTVCGMWKGYGC</td></tr>
<tr><td>>000319373|e2okeA1|70.2.1.2|A:9-131</td><td>123</td><td>PVRFVKETNRAKSPTRQSPGAAGYDLYSAYDYTIPPGERQLIKTDISMSMPKFCYGRIAPRSGLSLKGIDIGGGVIDEDYRGNIGVILINNGKCTFNVNTGDRIAQLIYQRIYYPELEEVQSL</td></tr>
<tr><td>>001309409|e4ngfB1|4.1.1.48|B:138-261</td><td>124</td><td>EKSEARIGIPSTKYTKETPFVFKLEDYQDAVIIPRYRNFDQPHRFYVADVYTDLTPLSKFPSPEYETFAEYYKTKYNLDLTNLNQPLLDVDHTSSRLNLLTPRHLNQKGKALPLSSAEKRKAKW</td></tr>
<tr><td>>001219409|e3of6A3|11.1.1.26|A:121-244</td><td>124</td><td>NVFPPEVAVFEPSEAEISHTQKATLVCLATGFYPDHVELSWWVNGKEVHSGVSTDPQPLKEQPALNDSRYCLSSRLRVSATFWQNPRNHFRCQVQFYGLSENDEWTQDRAKPVTQIVSAEAWGR</td></tr>
<tr><td>>001444345|e4v97BD2|2.1.1.1|BD:2-126</td><td>125</td><td>AVKKFKPYTPSRRFMTVADFSEITKTEPEKSLVKPLKKTGGRNNQGRITVRFRGGGHKRLYRIIDFKRWDKVGIPAKVAAIEYDPNRSARIALLHYVDGEKRYIIAPDGLQVGQQVVAGPDAPIQ</td></tr>
<tr><td>>000135509|e3dojA2|129.1.1.10|A:184-309</td><td>126</td><td>VGNGAKMKLIVNMIMGSMMNAFSEGLVLADKSGLSSDTLLDILDLGAMTNPMFKGKGPSMNKSSYPPAFPLKHQQKDMRLALALGDENAVSMPVAAAANEAFKKARSLGLGDLDFSAVIEAVKFSR</td></tr>
<tr><td>>001546381|e4ymdA1|209.1.1.5|A:29-154</td><td>126</td><td>AGVRETESKIYLLVKEEKRYADAQLSCQGRGGTLSMPKDEAANGLMAAYLAQAGLARVFIGINDLEKEGAFVYSDHSPMRTFNKWRSGEPNNAYDEEDCVEMVASGGWNDVACHTTMYFMCEFDKE</td></tr>
<tr><td>>000051446|e2fwlA1|107.1.1.15|A:5-133</td><td>129</td><td>DGAKIYAQCAGCHQQNGQGIPGAFPPLAGHVAEILAKEGGREYLILVLLYGLQGQIEVKGMKYNGVMSSFAQLKDEEIAAVLNHIATAWGDAKKVKGFKPFTAEEVKKLRAKKLTPQQVLAERKKLGLK</td></tr>
<tr><td>>000083259|e1xekA1|235.1.1.9|A:1-129</td><td>129</td><td>KVFGRCELAAAMKRHGLDNYRGYSLGNWVCAAKFESNFNTQATNRNTDGSTDYGILQINSRWWCNDGRTPGSRNLCNIPCSALLSSDITASVNCAKKIVSDGNGMNAWVAWRNRCKGTDVQAWIRGCRL</td></tr>
<tr><td>>000083137|e2bqdA1|235.1.1.9|A:1-130</td><td>130</td><td>KVFERCELARTLKRLGMDGYRGISLANWMCLAKWESGYNTRATNYNAGDRSTDYGVFQINSRYWCNDGKTPGAVNAAHLSCSALLQDNIADAVAAAKRVVRDPQGIRAWVAWRNRCQNRDVRQYVQGCGV</td></tr>
<tr><td>>001511254|e3uthB1|244.1.1.54|B:294-424</td><td>131</td><td>FYSSTHVIGVGVRGSRPERIGDKCWLYFPEDNCPFYRATIFSNYSPYNQPEASAALPTMQLADGSRPQSTEAKEGPYWSIMLEVSESSMKPVNQETILADCIQGLVNTEMLKPTDEIVSTYHRRFDHGYPT</td></tr>
<tr><td>>000417529|e3o44N3|76.1.1.5|N:460-593</td><td>134</td><td>AQESSPILGYTQGKMNQQRVGQDNRLYVRAGAAIDALGSASDLLVGGNGGSLSSVDLSGVKSITATSGDFQYGGQQLVALTFTYQDGRQQTVGSKAYVTNAHEDRFDLPDAAKITQLKIWADDWLVKGVQFDLN</td></tr>
<tr><td>>000055300|e2rcwA1|609.1.1.1|A:1-135</td><td>135</td><td>KSKLPKPVQDLIKMIFDVESMKKAMVEYEIDLQKMPLGKLSKRQIQAAYSILSEVQQAVSQGSSDSQILDLSNRFYTLIPHDFGMKKPPLLNNADSVQAKVEMLDNLLDIEVAYSLLRGGSDDSSKDPIDVNYEK</td></tr>
<tr><td>>000027799|e1bm5A1|9.1.1.10|A:2-137</td><td>136</td><td>NFSGNWKIIRSENFEELLKVLGVNVMLRKIAVAAASKPAVEIKQEGDTFYIKTSTTVRTTEINFKVGEEFEEQTVDGRPCKSLVKWESENKMVCEQKLLKGEGPKTSWTMELTNDGELILTMTADDVVCTRVYVRE</td></tr>
<tr><td>>000050274|e2dn2A1|106.1.1.7|A:2-141</td><td>140</td><td>LSPADKTNVKAAWGKVGAHAGEYGAEALERMFLSFPTTKTYFPHFDLSHGSAQVKGHGKKVADALTNAVAHVDDMPNALSALSDLHAHKLRVDPVNFKLLSHCLLVTLAAHLPAEFTPAVHASLDKFLASVSTVLTSKYR</td></tr>
<tr><td>>001552827|e4u4nC61|212.1.1.17|C6:2-142</td><td>141</td><td>AVPSVQTFGKKKSATAVAHVKAGKGLIKVNGSPITLVEPEILRFKVYEPLLLVGLDKFSNIDIRVRVTGGGHVSQVYAIRQAIAKGLVAYHQKYVDEQSKNELKKAFTSYDRTLLIADSRRPEPKKFGGKGARSRFQKSYR</td></tr>
<tr><td>>000098054|e1gv1A2|2003.1.1.40|A:1-142</td><td>142</td><td>MKITVIGAGNVGATTAFRIADKKLARELVLLDVVEGIPQGKGLDMYETGPVGLFDTKITGSNDYADTADSDIVIITAGLPRKPGMTREDLLMKNAGIVKEVTDNIMKHSKNPIIIVVSNPLDIMTHVAWVRSGLPKERVIGM</td></tr>
<tr><td>>001424000|e4u20DL1|2490.3.1.1|DL:1-143</td><td>143</td><td>RLNTLSPAEGSKKAGKRLGRGIGSGLGKTGGRGHKGQKSRSGGGVRRGFEGGQMPLYRRLPKFGFTSRKAAITAEIRLSDLAKVEGGVVDLNTLKAANIIGIQIEFAKVILAGEVTTPVTVRGLRVTKGARAAIEAAGGKIEE</td></tr>
<tr><td>>001209349|e4indT9|10.1.2.108|T:6-152</td><td>147</td><td>LGQSFPANAKVKYYYKLSEKQDLDAFVNSIFVGSYKLKQISYLLYGNTKIVSAPVVPLGPNASIIIDDELQEGLYLIRIKVYNTNSFSVTVTPFFNNNNTMTYSIGANSEFEIYDIFTKEQGNIYYIQLPPGLAILEFSLERVFEKG</td></tr>
<tr><td>>001012358|e1vvfQ2|170.1.1.6|Q:1-148</td><td>148</td><td>PIVQNLQGQMVHQAISPRTLNAWVKVVEEKAFSPEVIPMFSALSEGATPQDLNTMLNTVGGHQAAMQMLKETINEEAAEWDRLHPVHAGPIEPGQMREPRGSDIAGTTSTLQEQIGWMTHNPPIPVGEIYKRWIILGLNKIVRMYSPT</td></tr>
<tr><td>>001207793|e4kzyC5|330.1.1.5|C:38-186</td><td>149</td><td>GKAGDKEWLPVTKLGRLVKDMKIKSLEEIYLFSLPIKESEIIDFFLGAALKDKVLKIMPVQKQTQAGQRTRFKAFVAIGDYNGHVGLGMKCSKEVATAIRGAIILAKLSIVPVRRGYWGNKIGKPHTVRCKVTGRCGSVLVRLIPAPRG</td></tr>
</table>

<table>
<tr><td>>000281736|e1ylzA2|223.3.1.9|A:1-39,A:154-263</td><td>149</td><td>QTSAVQQKLAALEKSSGGRLGVALIDTADNTQVLYRGDEDTTTPRAMAQTLRQLTLGHALGETQRAQLVTWLKGNTTGAASIRAGLPTSWTVGDKTGSGDYGTTNDIAVIWPQGRAPLVLVTYFTQPQQNAESRRDVLASAARIIAEGL</td></tr>
<tr><td>>001291444|e4kiuX1|2007.8.1.1|X:1-150</td><td>150</td><td>LYFQSHMSELIVNVINGPNLGRLGRREPAVYGGTTHDELVALIEREAAELGLKAVVRQSDSEAQLLDWIHQAADAAEPVILNAGGLTHTSVALRDACAELSAPLIEVHISNVHAREEFRRHSYLSPIATGVIVGLGIQGYLLALRYLAEH</td></tr>
<tr><td>>000151329|e4dohA1|150.3.1.8|A:1-153</td><td>153</td><td>ALKTLNLGSCVIATNLQEIRNGFSEIRGSVQAKDGNIDIRILRRTESLQDTKPANRCCLLRHLLRLYLDRVFKNYQTPDHYTLRKISSLANSFLTIKKDLRLCHAHMTCHCGEEAMKKYSQILSHFEKLEPQAAVVKALGELDILLQWMEETE</td></tr>
<tr><td>>000000696|e2ahmE1|4315.1.1.1|E:43-197</td><td>155</td><td>LKKSLNVAKSEFDRDAAMQRKLEKMADQAMTQMYKQARSEDKRAKVTSAMQTMLFTMLRKLDNDALNNIINNARDGCVPLNIIPLTTAAKLMVVVPDYGTYKNTCDGNTFTYASALWEIQQVVDADSKIVQLSEINMDNSPNLAWPLIVTALRAN</td></tr>
<tr><td>>001440067|e4v7zAG1|167.1.1.1|AG:2-156</td><td>155</td><td>ARRRRAEVRQLQPDLVYGDVLVTAFINKIMRDGKKNLAARIFYDACKIIQEKTGQEPLKVFKQAVENVKPRMEVRSRRVGGANYQVPMEVSPRRQQSLALRWLVQAANQRPERRAAVRIAHELMDAAEGKGGAVKKKEDVERMAEANRAYAHYRW</td></tr>
<tr><td>>000984295|e4e8iB1|316.1.1.45|B:1-158</td><td>158</td><td>XKNNNVTEKELFYILDLFEHMKVTYWLDGGWGVDVLTGKQQREHRDIDIDFDAQHTQKVIQKLEDIGYKIEVHWMPSRMELKHEEYGYLDIHPINLNDDGSITQANPEGGNYVFQNDWFSETNYKDRKIPCISKEAQLLFHSGYDLTETDHFDIKNLK</td></tr>
<tr><td>>000378954|e3ko3C1|2494.1.1.2|C:1-160</td><td>160</td><td>MRVVIQRVKGAILSVRKENIGENEKELEIISEIKNGLICFLGIHKNDTWEDALYIIRKCLNLRLWNNDNKTWDKNVKDLNYELLIVSQFTLFGNTKKGNKPDFHLAKEPNEALIFYNKIIDEFKKQYNDDKIKIGKFGNYMNIDVTNDGPVTIYIDTHDI</td></tr>
<tr><td>>000430055|e3trhP1|2007.11.1.1|P:6-169</td><td>164</td><td>KIFVAILMGSDSDLSTMETAFTELKSLGIPFEAHILSAHRTPKETVEFVENADNRGCAVFIAAAGLAAHLAGTIAAHTLKPVIGVPMAGGSLGGLDALLSTVQMPGGVPVACTAIGKAGAKNAAILAAQIIALQDKSIAQKLVQQRTAKRETLKKADENLQTQL</td></tr>
<tr><td>>000309681|e2d52A2|2111.79.1.4|A:241-405</td><td>165</td><td>PIIGVEKPMFEIVCTKQTVIPNTEDVIHLHLRETGMMFYLSKGSPMTISNNVEACLIDVFKSVGITPPEDWNSLFWIPHPGGRAILDQVEAKLKLRPEKFRAARTVLWDYGNMVSASVGYILDEMRRKSAAKGLETYGEGLEWGVLLGFGPGITVETILLHSLPL</td></tr>
<tr><td>>000421126|e2yg2A1|9.1.1.1|A:7-172</td><td>166</td><td>CPEHSQLTTLGVDGKEFPEVHLGQWYFIAGAAPTKEELATFDPVDNIVFNMAAGSAPMQLHLRATIRMKDGLCVPRKWIYHLTEGSTDLRTEGRPDMKTELFSSSCPGGIMLNETGQGYQRFLLYNRSPHPPEKCVEEFKSLTSCLDSKAFLLTPRNQEACELSNN</td></tr>
<tr><td>>001180847|e3mmbA10|304.37.1.1|A:2-167</td><td>166</td><td>SETPLLDELEKGPWPSFVKEIKKTAELMEKAAAEGKDVKMPKGARGLLKQLEISYKDKKTHWKHGGIVSVVGYGGGVIGRYSDLGEQIPEVEHFHTMRINQPSGWFYSTKALRGLCDVWEKWGSGLTNFHGSTGDIIFLGTRSEYLQPCFEDLGNLEIPFDIGGSG</td></tr>
<tr><td>>000289873|e1x9eA1|2111.79.1.11|A:1-166</td><td>166</td><td>MTIGIDKISFFVPPYYIDMTALAEARNVDPGKFHIGIGQDQMAVNPISQDIVTFAANAAEAILTKEDKEAIDMVIVGTESSIDESKAAAVVLHRLMGIQPFARSFEIKEACYGATAGLQLAKNHVALHPDKKVLVVAADIAKYGLNSGGEPTQGAGAVAMLVASEP</td></tr>
<tr><td>>000988639|e3pymB2|2003.1.1.36|B:1-148,B:313-331</td><td>167</td><td>MVRVAINGFGRIGRLVMRIALSRPNVEVVALNDPFITNDYAAYMFKYDSTHGRYAGEVSHDDKHIIVDGKKIATYQERDPANLPWGSSNVDIAIDSTGVFKELDTAQKHIDAGAKKVVITAPSSTAPMFVMGVNEEKYTSDLKIVSNADNEYGYSTRVVDLVEHVAK</td></tr>
<tr><td>>000101756|e1guaA1|2004.1.1.144|A:1-167</td><td>167</td><td>MREYKLVVLGSGGVGKSALTVQFVQGIFVDEYDPTIEDSYRKQVEVDCQQCMLEILDTAGTEQFTAMRDLYMKNGQGFALVYSITAQSTFNDLQDLREQILRVKDTEDVPMILVGNKCDLEDERVVGKEQGQNLARQWCNCAFLESSAKSKINVNEIFYDLVRQINR</td></tr>
<tr><td>>000286359|e2a67D1|2111.61.1.1|D:1-167</td><td>167</td><td>AMKNRALLLIDFQKGIESPTQQLYRLPAVLDKVNQRIAVYRQHHAPIIFVQHEETELPFGSDSWQLFEKLDTQPTDFFIRKTHANAFYQTNLNDLLTEQAVQTLEIAGVQTEFCVDTTIRMAHGLGYTCLMTPKTTSTLDNGHLTAAQIIQHHEAIWAGRFLTFLSL</td></tr>
<tr><td>>000023654|e1ph9A1|2.1.1.35|A:1-169</td><td>169</td><td>YEYVELAKASLTSAQPQHFYAVVIDATFPYKTNQERYICSLKIVDPTLYLKQQKGAGDASDYATLVLYAKRFEDLPIIHRAGDIIRVHRATLRLYNGQRQFNANVFYSSSWALFSTDKRSVTQEINNQDAVSDTTPFSFSSKHATIEKNEISILQNLRKWANQYFSSYS</td></tr>
<tr><td>>001502538|e4xy3A1|150.8.1.1|A:117-285</td><td>169</td><td>ELTDTPRVATAGEPNFMDLKEAARKLETGDQGASLAHFADGWNTFNLTLQGDVKRFRGFDNWEGDAATACEASLDQQRQWILHMAKLSAAMAKQAQYVAQLHVWARREHPTYEDIVGLERLYAENPSARDQILPVYAEYQQRSEKVLTEYNNKAALEPVNPPKPPPAIK</td></tr>
<tr><td>>001427252|e4u56m11|882.1.1.1|m1:5-173</td><td>169</td><td>QNPMRDLKIEKLVLNISVGESGDRLTRASKVLEQLSGQTPVQSKARYTVRTFGIRRNEKIAVHVTVRGPKAEEILERGLKVKEYQLRDRNFSATGNFGFGIDEHIDLGIKYDPSIGIFGMDFYVVMNRPGARVTRRKRCKGTVGNSHKTTKEDTVSWFKQKYDADVLDK</td></tr>
<tr><td>>000223426|e4jcpA1|75.1.1.6|A:6-175</td><td>170</td><td>SRPKVFFDITIGGSNAGRIVMELFADIVPKTAENFRCLCTGERGMGRSGKKLHYKGSKFHRVIPNFMLQGGDFTRGNGTGGESIYGEKFPDENFQEKHTGPGVLSMANAGPNTNGSQFFICTAKTEWLDGKHVVFGRVVEGMNVVKAVESKGSQSGRTSADIVISDCGQL</td></tr>
<tr><td>>000142411|e3nb3A1|5084.1.1.5|A:22-192</td><td>171</td><td>APKDNTWYTGAKLGWSQYHDTGFINNNGPTHENKLGAGAFGGYQVNPYVGFEMGYDWLGRMPYKGSVENGAYKAQGVQLTAKLGYPITDDLDIYTRLGGMVWRADTYSNVYGKNHDTGVSPVFAGGVEYAITPEIATRLEYQWTNNIGDAHTIGTRPDNGMLSLGVSYRFG</td></tr>
<tr><td>>001036377|e2yo1A3|208.2.1.2|A:42-158,A:211-268</td><td>175</td><td>KYYHANSTEEDSLAVGTDSLAMGAKTIVNADAGIGIGLNTLVMADAINGIAIGSNARANHANSIAMGNGSQTTRGAQTDYTAYNMDTPQNSVGEFSVGSEDGQRQITNVAAGSADTDYFKTNTDGADANAQGADSVAIGSGSIAAAENSVALGTNSVADEANTVSVGSSTQQRRI</td></tr>
<tr><td>>000138051|e3jvgA2|233.1.1.2|A:5-179</td><td>175</td><td>SHMLKLLHFATFQNSTSVLVGGLGLLGDVKMGSLDSRTGNIRYYRPWLRPSLPKGDWDVIESSIKSYVRDFSRLVQMYTTVPYPFVFQSSIGCELQSNGTIRTFFDIAYEGQNFLRFNLDAGTWDQMQHNQLSAKAEHLMANASTLNEVIQVLLNDTCVDILRLFIQAGKADLER</td></tr>
<tr><td>>000347248|e3c9vC3|2487.1.1.20|C:190-365</td><td>176</td><td>EGMQFDRGYLSPYFINKPETGAVELESPFILLADKKISNIREMLPVLEAVAKAGKPLLIIAEDVEGEALATLVVNTMRGIVKVAAVKAPGFGDRRKAMLQDIATLTGGTVISEEIGMELEKATLEDLGQAKRVVINKDTTTIIDGVGEEAAIQGRVAQIRQQIEEATSDYDREKLQ</td></tr>
<tr><td>>001249254|e2x0bA2|1.1.1.2|A:51-228</td><td>178</td><td>SSVILTNYMDTQYYGEIGIGTPPQTFKVVFDTGSSNVWVPSSKCSRLYTACVYHKLFDASDSSSYKHNGTELTLRYSTGTVSGFLSQDIITVGGITVTQMFGEVTEMPALPFMLAEFDGVVGMGFIEQAIGRVTPIFDNIISQGVLKEDVFSFYYNRDSENSQSLGGQIVLGGSDPQH</td></tr>
<tr><td>>000289247|e1wrvB1|4045.1.1.2|B:125-306</td><td>182</td><td>EWGAYLGEEAVRKGARLITSSWARFPANVMPGKAKVGGNYVNSALAKMEAVAAGADEALLLDEEGYVAEGSGENLFFVRDGVIYALEHSVNLEGITRDSVIRIAKDLGYEVQVVRATRDQLYMADEVFMTGTAAEVTPVSMIDWRPIGKGTAGPVALRLREVYLEAVTGRRPEYEGWLTYVN</td></tr>
<tr><td>>000961996|e3v5eJ1|2486.1.1.4|J:1-184</td><td>184</td><td>MNLIPTVIETTNRGERAYDIYSRLLKDRIIMLGSQIDDNVANSIVSQLLFLQAQDSEKDIYLYINSPGGSVTAGFAIYDTIQHIKPDVQTICIGMAASMGSFLLAAGAKGKRFALPNAEVMIHQPLGGAQGQATEIEIAANHILKTREKLNRILSERTGQSIEKIQKDTDRDNFLTAEEAKEYG</td></tr>
<tr><td>>001390394|e4ariA1|140.1.1.4|A:696-880</td><td>185</td><td>LVYEHTAKGDVAALNVDALTENQKALRRDVHKTIAKVTDDIGRRQTFNTAIAAIMELMNKLAKAPTDGEQDRALMQEALLAVVRMLNPFTPHICFTLWQELKGEGDIDNAPWPVADEKAMVEDSTLVVVQVNGKVRAKITVPVDATEEQVRERAGQEHLVAKYLDGVTVRKVIYVPGKLLNLVVG</td></tr>
<tr><td>>000074471|e1nv4A1|4018.1.1.2|A:8-199</td><td>192</td><td>TNIVTLTRFVMEEGRKARGTGEMTQLLNSLCTAVKAISTAVRKAGIAHLYGIAGSTNVTGDQVKKLDVLSNDLVINVLKSSFATCVLVSEEDKNAIIVEPEKRGKYVVCFDPLDGSSNIDCLVSIGTIFGIYRKNSTDEPSEKDALQPGRNLVAAGYALYGSATMLVLAMVNGVNCFMLDPAIGEFILVDRD</td></tr>
<tr><td>>000107503|e2j4eF1|2111.12.1.2|F:3-195</td><td>193</td><td>MAASLVGKKIVFVTGNAKKLEEVVQILGDKFPCTLVAQKIDLPEYQGEPDEISIQKCQEAVRQVQGPVLVEDTCLCFNALGGLPGPYIKWFLEKLKPEGLHQLLAGFEDKSAYALCTFALSTGDPSQPVRLFRGRTSGRIVAPRGCQDFGWDPCFQPDGYEQTYAEMPKAEKNAVSHRFRALLELQEYFGSLA</td></tr>
<tr><td>>001189411|e3zbiJ2|517.2.1.1|J:1-152,J:174-216</td><td>195</td><td>ETSEGSSALAKNLTPARLKASRAGVMANPSLTVPKGKMIPCGTGTELDTTVPGQVSCRVSQDVYSADGLVRLIDKGSWVDGQITGGIKDGQARVFVLWERIRNDQDGTIVNIDSAGTNSLGSAGIPGQVDAHMWERLRGAIMISLFSDTLTALASEALRSYMSIPPTLYDQQGDAVSIFVARDLDFSGVYTLADN</td></tr>
<tr><td>>001400620|e4oggC2|2007.1.1.1|C:194-388</td><td>195</td><td>ANKRILFLCGDYMEDYEVKVPFQSLQALGCQVDAVCPEKKAGDRCPTAIHDFEGDQTYSEKPGHTFALTTNFDDLVSSSYDALVIPGGRAPEYLALNEHVLNIVKEFMNSEKPVASICHGQQILAAAGVLKGRKCTAYPAVKLNVVLGGGTWLEPDPIDRCFTDGNLVTGAAWPGHPEFVSQLMALLGIQVSFHH</td></tr>
<tr><td>>000418926|e3q05C1|11.1.5.28|C:1-200</td><td>200</td><td>SSSVPSQKTYQGSYGFRLGFLHSGTAKSVTCTYSPALNKMFVQLAKTVPVQLYVDSTPPPGTRVRAMAIYKQSQHMTEVVRRCPHHERSSDSDGLAPPQHLIRVEGNLRAEYLDDPNTFRHSVVVPYEPPEVGSDYTTIYFKFMCNSSCMGGMNRRPILVIITLEDSSGNLLGRDSFEVRVCACPGRDRRTEEENLRKKT</td></tr>
<tr><td>>001242405|e4a2dA9|3156.1.1.6|A:297-496</td><td>200</td><td>NPLVESALTTLKGTAAPGSPTPGGVDLALNMAFGFAGGNFTINGASFTPPTVPVLLQILSGAQSAADLLPAGSVYSLPANADIEISLPATAAAPGFPHPFHLHGHVFAVVRSAGSSTYNYANPVYRDVVSTGAPGDNVTIRFRTDNPGPWFLHCHIDFHLEAGFAVVMAEDIPDVAATNPVPQAWSDLCPTYDALSPDDQ</td></tr>
<tr><td>>000098233|e1mv8B2|2003.1.1.65|B:1-202</td><td>202</td><td>MRISIFGLGYVGAVCAGCLSARGHEVIGVDVSSTKIDLINQGKSPIVEPGLEALLQQGRQTGRLSGTTDFKKAVLDSDVSFICVGTPSKKNGDLDLGYIETVCREIGFAIREKSERHTVVVRSTVLPGTVNNVVIPLIEDCSGKKAGVDFGVGTNPEFLRESTAIKDYDFPPMTVIGELDKQTGDLLEEIYRELDAPIIRKT</td></tr>
<tr><td>>001348172|e4w27O1|2490.2.1.1|O:3-204</td><td>202</td><td>EGQQVLVLDGRGHLLGRLAAIVAKQVLLGRKVVVVRCEGINISGNFYRNKLKYLAFLRKRMNTNPSRGPYHFRAPSRIFWRTVRGMLPHKTKRGQAALDRLKVFDGIPPPYDKKKRMVVPAALKVVRLKPTRKFAYLGRLAHEVGWKYQAVTATLEEKRKEKAKIHYRKKKQLMRLRKQAEKNVEKKIDRFTEVLKTHGFLV</td></tr>
<tr><td>>001180010|e1gqhA2|10.12.1.74|A:2-205</td><td>204</td><td>TSSLIVEDAPDHVRPYVIRHYSHARAVTVDTQLYRFYVTGPSSGYAFTLMGTNAPHSDALGVLPHIHQKHYENFYCNKGSFQLWAQSGNETQQTRVLSSGDYGSVPRNVTHTFQIQDPDTEMTGVIVPGGFEDLFYYLGTNATDTTHTPYIPSSSDSSSTTGPDSSTISTLQSFDVYAELSFTPRTDTVNGTAPANTVWHTGAN</td></tr>
<tr><td>>001414667|e1zxjD1|589.1.2.5|D:14-217</td><td>204</td><td>IPTTENLYFQGHMATNLKSTAKLVKPIQYDEVIEVERIFADPAFIEQHRQRILASFKDAKESALYHELTHIVIKDNLFSCAMNAIVGYFEFNIDEAELKNVMEGLKRDVIQGAEDNTVQAIAEKIIKKALVFNHLQKEWKVEITDEVVKNVISLYYEKTNQSVREYLDDKQKFEGVRTALLEERMVLETINHFKFHFNLTGQLP</td></tr>
</table>

>001200249|e4hujA2|2003.1.1.30|A:17-220	204	NLYFQSMTTYAIIGAGAIGSALAERFTAAQIPAIIANSRGPASLSSVTDRFGASVKAVELKDALQADVVILAVPYDSIADIVTQVSDWGGQIVVDASNAIDFPAFKPRDLGGRLSTEIVSELVPGAKVVKAFNTLPAAVLAADPDKGTGSRVLFLSGNHSDANRQVAELISSLGFAPVDLGTLAASGPIQQFGRPLVALNLLKD
>001063737|e4jn3B2|323.1.1.10|B:3-207	205	SENSSVRHGLTSAQHEVWLAQQLDPRGAHYRTGSCLEIDGPLDHAVLSRALRLTVAGTETLCSRFLTDEEGRPYRAYCPPAPEGSAAVEDPDGVPYTPVLLRHIDLSGHEDPEGEAQRWMDRDRATPLPLDRPGLSSHALFTLGGGRHLYYLGVHHIVIDGTSMALFYERLAEVYRALRDGRAVPAAAFGDTDRMVAGEEAYRAS
>001247165|e3hwwD4|2111.75.1.6|D:2-207	206	SVSAFNRRWAAVILEALTRHGVRHICIAPGSRSTLLTLAAAENSAFIHHTHFDERGLGHLALGLAKVSKQPVAVIVTSGTAVANLYPALIEAGLTGEKLILLTADRPPELIDCGANQAIRQPGMFASHPTHSISLPRPTQDIPARWLVSTIDHALGTLHAGGVHINCPFAEPLYGEMDDTGLSWQQRLGDWWQDDKPWLREAPRLE
>000371883|e2wnlC1|11.12.1.1|C:6-216	211	DKLHSQANLMRLKSDLFNRSPMYPGPTKDDPLTVTLGFTLQDIVKADSSTNEVDLVYYEQQRWKLNSLMWDPNEYGNITDFRTSAADIWTPDITAYSSTRPVQVLSPQIAVVTHDGSVMFIPAQRLSFMCDPTGVDSEEGATCAVKFGSWVYSGFEIDLKTDTDQVDLSSYYASSKYEILSATQTRQVQHYSCCPEPYIDVNLVVKFRERR
>000979181|e4g8wA1|150.1.2.6|A:1-213	213	MERPQLDSMSQDLSEALKEATKEVHIRAENSEFMRNFQKGQVSREGFKLVMASLYHIYTALEEEIERNKQNPVYAPLYFPEELHRRAALEQDMAFWYGPHWQEAIPYTPATQHYVKRLHEVGGTHPELLVAHAYTRYLGDLSGGQVLKKIAQKAMALPSSGEGLAFFTFPSIDNPTKFKQLYRARMNTLEMTPEVKHRVTEEAKTAFLLNIEL
>000969506|e3rluA1|2002.1.1.140|A:1-214	214	MRSRRVDVMDVMNRLILAMDLMNRDDALRVTGEVREYIDTVKIGYPLVLSEGMDIIAEFRKRFGCRIIADFKVADIPETNEAICRATFKAGADAIIVHGFPGADSVRACLNVAEEMGREVFLLTEMSHPGAEMFIQGAADEIARMGVDLGVKNYVGPSTRPERLSRLREIIGQDSFLISPGVGAQGGDPGETLRFADAIIVGRSIYLADNPAAA
>000140571|e3a7dA1|2003.1.5.35|A:4-217	214	GDTKEQRILRYVQQNAKPGDPQSVLEAIDTYCTQKEWAMNVGDAKGQIMDAVIREYSPSLVLELGAYCGYSAVRMARLLQPGARLLTMEMNPDYAAITQQMLNFAGLQDKVTILNGASQDLIPQLKKKYDVDTLDMVFLDHWKDRYLPDTLLLEKCGLLRKGTVLLADNVIVPGTPDFLAYVRGSSSFECTHYSSYLEYMKVVDGLEKAIYQGP
>000087811|e1dd6B1|247.1.1.6|B:4-220	217	LPDLKIEKLDEGVYVHTSFEEVNGWGVVPKHGLVVLVNAEAYLIDTPFTAKDTEKLVTWFVERGYKIKGSISSHFHSDSTGGIEWLNSRSIPTYASELTNELLKKDGKVQATNSFSGVNYWLVKNKIEVFYPGPGHTPDNVVVWLPERKILFGGCFIKPYGLGNLGDANIEAWPKSAKLLKSKYGKAKLVVPSHSEVGDASLLKLTLEQAVKGLNES
>001233584|e1kyiT3|2004.1.1.17|T:2-120,T:233-330	217	SEMTPREIVSELDQHIIGQADAKRAVAIALRNRWRRMQLQEPLRHEVTPKNILMIGPTGVGKTEIARRLAKLANAPFIKVEATKFTEVGYVGKEVDSIIRDLTDSAMKLVRQQEIAKNRKLINPEELKQKAIDAVEQNGIVFIDEIDKICKKGEYSGADVSREGVQRDLLPLVEGSTVSTKHGMVKTDHILFIASGAFQVARPSDLIPELQGRLPIR
>001279114|e2pssA2|2003.1.5.97|A:98-321	224	DEFAYHEMMTHVPMTVSKEPKNVLVVGGGDGGIIRELCKYKSVENIDICEIDETVIEVSKIYFKNISCGYEDKRVNVFIEDASKFLENVTNTYDVIIVDSSDPIGPAETLFNQNFYEKIYNALKPNGYCVAQCESLWIHVGTIKNMIGYAKKLFKKVEYANISIPTYPCGCIGILCCSKTDTGLTKPNKKLESKEFADLKYYNYENHSAAFKLPAFLLKEIENI
>000298019|e2g9zB1|2111.62.1.1|B:19-244	226	ELIEQVIEQPDSLIISPPSDSSSSSYNHIQPFVYLESTATTQTNHNVLLILNQKITIDLISLWKKCEIIVCADGGANSLYEYFNDNNHHHHHENLQRSDYIPDYIVGDFDSISPDVKTYYESHGSKIIRQSSQYYNDFTKSIHCIQLHYQLNHTKENWFESIDEVDGLAKLWNGLNNSSDVVVDIDITIYVLNAIGGRFDQTVQSINQLYIMNEDYPKVTVFFITT
>000144444|e3awjA1|2111.79.1.7|A:23-248	226	VIKPDGPATILAIGTSNPTNIFEQSTYPDFFFDVTNCNDKTELKKKFQRICDKSGIKKRHFHLTDEILRKNPSICKFKEASLDPRQDIAVLEVPKLAKEAAISAIKQWGQPKSKITHLVFATTSGVDMPGADFQLAKLLGLRPTVKRVMLYQQGCYAGATVLRVAKDLAENNKGARVLVACSEVTAVTFRAPSETHLDGLVGSALFGDGAAALIVGSDPVPQEEKP
>000359069|e3gemB1|2003.1.1.195|B:25-258	234	LSSAPILITGASQRVGLHCALRLLEHGHRVIISYRTEHASVTELRQAGAVALYGDFSCETGIMAFIDLLKTQTSSLRAVVHNASEWLAETPGEEADNFTRMFSVHMLAPYLINLHCEPLLTASEVADIVHISDDVTRKGSSKHIAYCATKAGLESLTLSFAARFAPLVKVNGIAPALLMFQPKDDAAYRANALAKSALGIEPGAEVIYQSLRYLLDSTYVTGTTLTVNGGRHVK
>001324185|e4u3jA1|2003.1.6.1|A:1-243	243	MREVISINVGQAGCQIGNACWELYSLEHGIKPDGHLEDGLSKPKGGEEGFSTFFHETGYGKFVPRAIYVDLEPNVIDEVRNGPYKDLFHPEQLISGKEDAANNYARGHYTVGREILGDVLDRIRKLADQCDGLQGFLFTHSLGGGTGSGLGSLLLEELSAEYGKKSKLEFAVYPAPQVSTSVVEPYNTVLTTHTTLEHADCTFMVDNEAIYDMCKRNLDIPRPSFANLNNLIAQVVSSVTASL
>000427537|e3qfoB1|246.2.1.7|B:11-260	250	MTKIALLSDIHGNTTALEAVLADARQLGVDEYWLLGDILMPGTGRRRILDLLDQLPITARVLGNWEDSLWHGVRKELDSTRPSQRYLLRQCQYVLEEISLEEIEVLHNQPLQIHRQFGDLTVGISHHLPDKNWGRELIHTGKQEEFDRLVTHPPCDIAVYGHIHQQLLRYGTGGQLIVNPGSIGQPFFLDAQLRKDLRAQYMILEFDDKGLVDMDFRRVDYDVAAELQLAKDLRLPYFEVYYESLVNGIH
>001201340|e4afnC2|2003.1.1.106|C:17-269	253	NLYFQSMSLQGKVALVTGASRGIGQAIALELGRLGAVVIGTATSASGAEKIAETLKANGVEGAGLVLDVSSDESVAATLEHIQQHLGQPLIVVNNAGITRDNLLVRMKDDEWFDVVNTNLNSLYRLSKAVLRGMTKARWGRIINIGSVVGAMGNAGQTNYAAAKAGLEGFTRALAREVGSRAITVNAVAPGFIDTDMTRELPEAQREALLGQIPLGRLGQAEEIAKVVGFLASDGAAYVTGATVPVNGGMYMS
>000044624|e1fqlA1|62.1.1.1|A:5-260	256	WGYGKHNGPEHWHKDFPIAKGERQSPVDIDTHTAKYDPSLKPLSVSYDQATSLRILNNGHAFNVEFDDSQDKAVLKGGPLDGTYRLIQFHMHVGSLDGQGSEHTVDKKKYAAELHLVHWNTKYGDFGKAVQQPDGLAVLGIFLKVGSAKPGLQKVVDVLDSIKTKGKSADFTNFDPRGLLPESLDYWTYPGSLTTPPLLECVTWIVLKEPISVSSEQVLKFRKLNFNGEGEPEELMVDNWRPAQPLKNRQIKASFK
>001288698|e4l5vA1|62.1.1.1|A:4-260	257	HWGYGKHNGPEHWHKDFPIAKGERQSPVDIDTHTAKYDPSLKPLSVSYDQATSLRILNNGHAFNVEFDDSQDKAVLKGGPLDGTYRLIQFHFHWGSLDGQGSEHTVDKKKYAAELHLVHWNTKYGDFGKAVQQPDGLAVLGIFLKVGSAKPGLQKVVDVLDSIKTKGKSADFTNFDPRGLLPESLDYWTYPGSLTTPPLLECVTWIVLKEPISVSSEQVLKLRKLNFNGEGEPEELMVDNWRPAQPLKNRQIKASFK
>000324072|e2e8rB1|2111.48.1.1|B:1-265	265	MVLYFIGLGLYDERDITVKGLEIAMKCDYVFAEFYTSLMAGTTLGRIQKLIGKEIRVLSREDVELNFENIVLPLAKENDVAFLTPGDPLVATTHAELRIRAKRAGVESYVIHAPSIYSAVGITGLHIYKFGKSATVAYPEGNWFPTSYYDVIKENAERGLHTLLFLDIKAEKRMYMTANEAMELLLKVEDMKKGGVFTDDTLVVVLARAGSLNPTIRAGYVKDLIREDFGDPPHILIVPGKLHIVEAEYLVEIAGAPREILRVNV
>000095977|e1dqxB1|2002.1.1.140|B:1-267	267	MHKATYKERAATHPSPVAAKLFNIMHEKQTNLCASLDVRTTKELLELVEALGPKICLLKTHVDILTDFSMEGTVKPLKALSAKYNFLLFEDRKFADIGNTVKLQYSAGVYRIAEWADITNAHGVVGPGIVSGLKQAAEEVTKEPRGLLMLAELSCKGSLSTGEYTKGTVDIAKSDKDFVIGFIAQRDMGGRDEGYDWLIMTPGVGLDDKGDALGQQYRTVDDVVSTGSDIIIVGRGLFAKGRDAKVEGERYRKAGWEAYLRRCGQQD
>000345920|e3dk3B1|206.1.1.24|B:4-276	273	DKWEMERTDITMKHKLGGGQYGEVYEGVWKKYSLTVAVKTLKEDTMEVEEFLKEAAVMKEIKHPNLVQLLGVCTREPPFYIITEFMTYGNLLDYLRECNRQEVSAVVLLYMATQISSAMEYLEKKNFIHRDLAARNCLVGENHLVKVADFGLSRLMTGDTFTAHAGAKFPIKWTAPESLAYNKFSIKSDVWAFGVLLWEIATYGMSPYPGIDPSQVYELLEKDYRMERPEGCPEKVYELMRACWQWNPSDRPSFAEIHQAFETMFQESSISDE
>000370490|e3eq8A2|2111.78.1.35|A:431-710	280	DASDYQTVQIFYPSKDGTKIPMFIVHKKGIKLDGSHPAFLYGYGGFNISITPNYSVSRLIFVRHMGGVLAVANIRGGGEYGETWHKGGILANKQNCFDDFQCAAEYLIKEGYTSPKRLTINGGSNGGLLVATCANQRPDLFGCVIAQVGVMDMLKFHKYTIGHAWTTDYGCSDSKQHFEWLIKYSPLHNVKLPEADDIQYPSMLLLTADHDDRVVPLHSLKFIATLQYIVGRSRKQNNPLLIHVDTKAGHGAGKPTAKVIEEVSDMFAFIARCLNIDWIP
>001031679|e4einA1|266.1.1.1|A:21-305	285	RHGELQYLRQVEHILRCGFKKEDRTGTGTLSVFGMQARYSLRDEFPLLTTKRVFWKGVLEELLWFIKGSTNAKELSSKGVRIWDANGSRDFLDSLGFSARQEGDLGPVYGFQWRHFGAEYKDMDSDYSGQGVDQLQKVIDTIKTNPDDRRIIMCAWNPKDLPLMALPPCHALCQFYVVNGELSCQLYQRSGDMGLGVPFNIASYALLTYMIAHITGLQPGDFVHTLGDAHIYLNHIEPLKIQLQREPRPFPKLKILRKVETIDDFKVEDFQIEGYNPHPTIKMEM
>000301605|e2gs7A1|206.1.1.25|A:11-291,A:317-320	285	LLRILKETEFKKIKVLGSGAFGTVYKGLWIPEGEKVKIPVAIKELREATSPKANKEILDEAYVMASVDNPHVCRLLGICLTSTVQLITQLMPFGCLLDYVREHKDNIGSQYLLNWCVQIAKGMNYLEDRRLVHRDLAARNVLVKTPQHVKITDFGLAKLLGAEEKEYHAEGGKVPIKWMALESILHRIYTHQSDVWSYGVTVWELMTFGSKPYDGIPASEISSILEKGERLPQPPICTIDVYMIMRKCWMIDADSRPKFRELIIEFSKMARDPQRYLVIQGDVVD
>001391908|e3g3qA1|868.1.1.7|A:8-293	286	NFVRQTTKYWVHPDNITELKLIILKHLPVLVFNTNKEFEREDSAITSIYFDNENLDLYYGRLRKDEGAEAHRLRWYGGMSTDTIFVERKTHREDWTGEKSVKARFALKERHVNDFLKGKYTVDQVFAKMRKEGKKPMNEIENLEALASEIQYVMLKKKLRPVVRSFYNRTAFQLPGDARVRISLDTELTMVREDNFDGVDRTHKNWRRTDIGVDWPFKQLDDKDICRFPYAVLEVKLQTQLGQEPPEWVRELVGSHLVEPVPKFSKFIHGVATLLNDKVDSIPFWL
>000136989|e3io7A1|206.1.1.24|A:23-311	289	TQFEERHLKFLQQLGKGNFGSVEMCRYDPLQDNTGEVVAVKKLQHSTEEHLRDFEREIEILKSLQHDNIVKYKGVCYSAGRRNLKLIMEYLPYGSLRDYLQKHKERIDHIKLLQYTSQICKGMEYLGTKRYIHRDLATRNILVENENRVKIGDFGLTKVLPQDKEYYKVKEPGESPIFWYAPESLTESKFSVASDVWSFGVVLYELFTYIEKSKSPPAEFMRMIGNDKQGQMIVFHLIELLKNNGRLPRPDGCPDEIYMIMTECWNNNVNQRPSFRDLALRVDQIRDNM
>000959507|e3qu0A1|206.1.1.18|A:1-294	294	GPLGSPEFMENFQKVEKIGEGTYGVVYKARNKLTGEVVALKKIRLDTETEGVPSTAIREISLLKELNHPNIVKLLDVIHTENKLYLVFEFLHQDLKKFMDASALTGIPLPLIKSYLFQLLQGLAFCHSHRVLHRDLKPQNLLINTEGAIKLADFGLARAFGVPVRTYTHEVVTLWYRAPEILLGCKYYSTAVDIWSLGCIFAEMVTRRALFPGDSEIDQLFRIFRTLGTPDEVVWPGVTSMPDYKPSFPKWARQDFSKVVPPLDEDGRSLLSQMLHYDPNKRISAKAALAHPFF
>001386286|e3f41A2|2009.1.2.7|A:36-329	294	PAVVKNPPKLALKIDRADVNQLPRNFRMGSDKYVGVTKTGIMPTRKGMDTMNVSASSCFSEKELEAILKKVPVKPSQFYDVDLRGESHGYLNGTAVSWFANHDWGNDGRTEDIIIPLEKEQLASLKGSTVKSIYRFDDKKNVILSPVYVNYNKVRTEEEMVKQHGANYFRLTLQDHFRPDDPDVDKFLEFYKSLPKDAWLHYHCYAGMGRTTIFMVMHDILKNAKDVSFDDIIQRQKLIGIVDLSEIPDKKKNYGRKAYIERYQFVQHFYDYVKENPDLKTPYSVWAKKNKVNS
>000433656|e3pb2E1|2002.1.1.37|E:5-300	296	FTMFRGVGTAIVTPFKNGELDLESYERLVRYQLENGVNALIVLGTTGESPTVNEDEREKLVSRTLEIVDGKIPVIVGAGTNSTEKTLKLVKQAEKLGANGVLVVTPYYNKPTQEGLYQHYKYISERTDLGIVVYNVPGRTGVNVLPETAARIAADLKNVVGIKEANPDIDQIDRTVSLTKQARSDFMVWSGNDDRTFYLLCAGGDGVISVVSNVAPKQMVELCAEYFSGNLEKSAEVHAKLRPLMKALFVETNPIPVKAALNLMGFIENELRLPLVPASEKTVELLRNVLKESGLL
>001193826|e4f5jA3|2111.77.1.27|A:8-306	299	HVGTFENITAAPADPILGLADLFRADERPGKVDLGVGVYKDETGKTPVLTSVKKAEQYLLENETTKTYLGLDGLPEFGRCTQELLFGKGSALINDKRARTAQTPGGSGALRVAADFLAKNTSVKRVWVSNPSWPNHKAIFNSAGLEVREYAYYDAENHTLDFDALINSLNEAQAGDVVLFHGCCHNPTGADPTLEQWQTLAQLSVEKGWLPLIDIAYQGFGRGLEEDAEGLRAFAAMHKELIVASSCSKNFGLYNERVGACTLVAADSETVDRAFSQMKAAIRANYSNPPAHGASVVAT
>000332250|e3b5wF1|5066.1.1.1|F:10-317	308	WQTFRRLWPTIAPFKAGLIVAGVALILNAASDTFMLSLLKPLLDDGFGKTDRSVLVWMPLVVIGLMILRGITSYVSSYCISWVSGKVVMTMRRRLFGHMMGMPVSFFDKQSTGTLLSRITYDSEQVASSSSGALITVVREGASIIGLFIMMFYYSWQLSIILIVLAPIVSIAIRVVSKRFRNISKNMQNTMGQVTTSAEQMLKGHKEVLIFGGQEVETKRFDKVSNRMRLQGMKMVSASSISDPIIQLIASLALAFVLYAASFPSVMDSLTAGTITVVFSSMIALMRPLKSLTNVNAQFQRGMAACQT
>000369252|e3ie7A1|2003.6.1.4|A:2-310	309	SLIYTITLNPAIDRLLFIRGELEKRKTNRVIKTEFDCGGKGLHVSGVLSKFGIKNEALGIAGSDNLDKLYAILKEKHINHDFLVEAGTSTRECFVVLSDDTNGSTMIPEAGFTVSQTNKDNLLKQIAKKVKKEDMVVIAGSPPPHYTLSDFKELLRTVKATGAFLGCDNSGEYLNLAVEMGVDFIKPNEDEVIAILDEKTNSLEENIRTLAEKIPYLVVSLGAKGSICAHNGKLYQVIPPKVQERNDTGAGDVFVGAFIAGLAMNMPITETLKVATGCSASKVMQQDSSSFDLEAAGKLKNQVSIIQLE
>000399872|e2wv7D1|2499.1.1.1|D:3-316	314	KFRLIPYKQVDKVSALSEVPMGVEIVEAPAVWRASAKGAGQIIGVIDTGCQVDHPDLAERIIGGVNLTTDYGGDETNFSDNNGHGTHVAGTVAAAETGSGVVGVAPKADLFIIKALSGDGSGEMGWIAKAIRYAVDWRGPKGEQMRIITMSLGGPTDSEELHDAVKYAVSNNVSVVCAAGNEGDGREDTNEFAYPAAYNEVIAVGAVDFDLRLSDFTNTNEEIDIVAPGVGIKSTYLDSGYAELSGTAMAAPHVAGALALIINLAEDAFKRSLSETEIYAQLVRRATPIGFTAQAEGNGFLTLDLVERITGQFT
>000356153|e3ei2A2|5.1.4.20|A:411-726	316	GIGIHEHASIDLPGIKGLWPLRSDPNRETDDTLVLSFVGQTRVLMLNGEEVEETELMGFVDDQQTFFCGNVAHQQLIQITSASVRLVSQEPKALVSEWKEPQAKNISVASCNSSQVVVAVGRALYYLQIHPQELRQISHTEMEHEVACLDITPLGDSNGLSPLCAIGLWTDISARILKLPSFELLHKEMLGGEIIPRSILMTTFESSHYLLCALGDGALFYFGLNIETGLLSDRKKVTLGTQPTVLRTFRSLSTTNVFACSDRPTVIYSSNHKLVFSNVNLKEVNYMCPLNSDGYPDSLALANNSTLTIGTIDEIQ
>001316465|e4unyA1|10.5.1.8|A:10-326	317	NTATLCLGHHAVANGTLVKTITDDQIEVTNATELVQSISIGKICNNSYRVLDGRNCTLIDAMLGDPHCDDFQYENWDLFIERSSAFSNCYPYDIPDYASLRSIVASSGTLEFTAEGFTWTGVTQNGGSGACKRGSADSFFSRLNWLTKSGNSYPILNVTMPNNKNFDKLYIWGIHHPSSNKEQTKLYIQESGRVTVSTERSQQTVIPNIGSRPWVRGQSGRISIYWTIVKPGDILMINSNGNLVAPRGYFKLRTGKSSVMRSDALIDTCVSECITPNGSIPNDKPFQNVNKITYGKCPKYIRQNTLKLATGMRNVPE
>000096775|e2vuuE1|2003.1.1.51|E:3-283,E:316-352	318	QQKKTIAVVNATGRQAASLIRVAAAVGHHVRAQVHSLKGLIAEELQAIPNVTLFQGPLLNNVPLMDTLFEGAHLAFINTTSQAGDEIAIGKDLADAAKRAGTIQHYIYSSMPDHSLYGPWPAVPMWAPKFTVENYVRQLGLPSTFVYAGIYNNNFTSLPYPLFQMELMPDGTFEWHAPFDPDIPLPWLDAEHDVGPALLQIFKDGPQKWNGHRIALTFETLSPVQVCAAFSRALNRRVTYVQVPKVEIKVNIPVGYREQLEAIEVVFGEHKAPYFPLPEFSRVTDEARKLWSGWRDMEEYAREVFPIEEEANGLDWML
>001436892|e4v6wAz2|2004.1.1.100|Az:4-327	324	FTVDEIRGLMDKKRNIRNMSVIAHVDHGKSTLTDSLVSKAGIIAGAKAGETRFTDTRKDEQERCITIKSTAISMYFEVEEKDLVFITHPDQREKECKGFLINLIDSPGHVDFSSEVTAALRVTDGALVVVDCVSGVCVQTETVLRQAIAERIKPILFMNKMDRALLELQLDAEELYQTFQRIVENVNVIIATYNDDGGPMGEVRVDPSKGSVGFGSGLHGWAFTLKQFSEMYSEKFKIDVVKLMNRLWGENFFNAKTKKWQKQKEADNKRSFCMYILDPIYKVFDAIMNYKKEEIGTLLEKIGVTLKHEDKDKDGKALLKTVMR

>001240342|e3bg3C8|2002.1.1.107|C:60-403	344	ASPSPTDPVVPAVPIGPPPAGFRDILLREGPEGFARAVRNHPGLLLMDTTFRDAHQSLLATRVRTHDLKKIAPYVAHNFSKLFSMENWGGATFDVAMRFLYECPWRRLQELRELIPNIPFQMLLRGANAVGYTNYPDNVVFKFCEVAKENGMDVFRVFDSLNYLPNMLLGMEAAGSAGGVVEAAISYTGDVADPSRTKYSLQYYMGLAEELVRAGTHILCIKDMAGLLKPTACTMLVSSLRDRFPDLPLHIHTHDTSGAGVAAMLACAQAGADVVDVAADSMSGMTSQPSMGALVACTRGTPLDTEVPMERVFDYSEYWEGARGLYAAFDCTATMKSGNSDVYE
>001111852|e4lniJ1|321.1.1.5|J:99-443	345	DGTPFEGDPRNNLKRILKEMEDLGFSDFNLGPEPEFFLFKLDEKGEPTLELNDKGGYFDLAPTDLGENCRRDIVLELEEMGFEIEASHHEVAPGQHEIDFKYAGAVRSCDDIQTFKLVVKTIARKHGLHATFMPKPLFGVNGSGMHCNLSLFKNGVNAFFDENADLQLSETAKHFIAGIVKHATSFTAVTNPTVNSYKRLVPGYEAPCYVAWSAQNRSPLIRIPASRGISTRVEVRSVDPAANPYLALSVLLAAGLDGIKNKLEAPAPIDRNIYVMSKEERMENGIVDLPATLAEALEEFKSNEVMVKALGEHLFEHFIEAKEIEWDMFRTQVHPWEREQYMSQY
>000067435|e1f52A1|321.1.1.5|A:101-468	368	DRDPRSIAKRAEDYLRATGIADTVLFGPEPEFFLFDDIRFGASISGSHVAIDDIEGAWNSSTKYEGGNKGHRPGVKGGYFPVPPVDSAQDIRSEMCLVMEQMGLVVEAHHHEVATAGQNEVATRFNTMTKKADEIQIYKYVVHNVAHRFGKTATFMPKPMFGDNGSGMHCHMSLAKNGTNLFSGDKYAGLSEQALYYIGGVIKHAKAINALANPTTNSYKRLVPGYEAPVMLAYSARNRSASIRIPVVASPKARRIEVRFPDPAANPYLCFAALLMAGLDGIKNKIHPGEPMDKNLYDLPPEEAKEIPQVAGSLEEALNALDLDREFLKAGGVFTDEAIDAYIALRREEDDRVRMTPHPVEFELYYSV
>001489228|e4rnvD1|2002.1.1.142|D:6-96,D:99-390	383	GGSNDFVYSIWKGPVIRAGNFALHPEVVREEVKDKRTLIGYGRFFISNPDLVDRLEKGLPLNKYDRDTFYQMSAHGYIDYPTYEEALKLGWSSFVKDFKPQALGDTNLFKPIKIGNNELLHRAVIPPLTRMRALHPGNIPNRDWAVEYYTQRAQRPGTMIITEGAFISPQAGGYDNAPGVWSEEQMVEWTKIFNAIHEKKSFVWVQLWVLGWAAFPDNLARDGLRYDSASDNVFMDAEQEAKAKKANNPQHSLTKDEIKQYIKEYVQAAKNSIAAGADGVEIHSANGYLLNQFLDPHSNTRTDEYGGSIENRARFTLEVVDALVEAIGHEKVGLRLSPYGVFNSMSGGAETGIVAQYAYVAGELEKRAKAGKRLAFVHLVEPR
>000044329|e3c0oB1|5088.1.1.1|B:85-468	384	IPTLSALDIPDGDEVDVQWRLVHDSANFIKPTSYLAHYLGYAWVGGNHSQYVGEDMDVTRDGDGWVIRGNNDGGCDGYRCGDKTAIKVSNFAYNLDPDSFKHGDVTQSDRQLVKTVVGWAVNDSDTPQSGYDVTLRGDTATNWSKTNTYGLSEKVTTKNKFKWPLVGETELSIEIAANQSWASQNGGSTTTSLSQSVRPTVPARSKIPVKIELYKADISYPYEFKADVSYDLTLSGFLRWGGNAWYTHPDNRPNWNHTFVIGPYKDKASSIRYQWDKRYIPGEVKWWDWNWTIQQNGLSTMQNNLARVLRPVRAGITGDFSAESQFAGNIEIGAPVPLAADSKVRRARSVDGAGQGLRLEIPLDAQELSGLGFNNVSLSVTPAA
>000315485|e2j9fA1|2111.75.1.2|A:6-400	395	KPQFPGASAEFIDKLEFIQPNVISGIPIYRVMDRQGQIINPSEDPHLPKEKVLKLYKSMTLLNTMDRILYESQRQGRISFYMTNYGEEGTHVGSAAALDNTDLVFGQYREAGVLMYRDYPLELFMAQCYGNISDLGKGRQMPVHYGCKERHFVTISSPLATQIPQAVGAAYAAKRANANRVVICYFGEGAASEGDAHAGFNFAATLECPIIFFCRNNGYAISTPTSEQYRGDGIAARGPGYGIMSIRVDGNDVFAVYNATKEARRRAVAENQPFLIEAMTYRIGHHSTSDDSSAYRPVDEVNYWDKQDHPISRLRHYLLSQGWWDEEQEKAWRKQSRRKVMEAFEQAERKPKPNPNLLFSDVYQEMPAQLRKQQESLARHLQTYGEHYPLDHFDK
>000101688|e1tq2B1|2004.1.1.109|B:14-413	400	NDLPSSFTGYFKKFNTGRKIISQEILNLIELRMRKGNIQLTNSAISDALKEIDSSVLNVAVTGETGSGKSSFINTLRGIGNEEEGAAKTGVVEVTMERHPYKHPNIPNVVFWDLPGIGSTNFPPDTYLEKMKFYEYDFFIIISATRFKKNDIDIAKAISMMKKEFYFVRTKVDSDITNEADGKPQTFDKEKVLQDIRLNCVNTFRENGIAEPPIFLLSNKNVCHYDFPVLMDKLISDLPIYKRHNFMVSLPNITDSVIEKKRQFLKQRIWLEGFAADLVNIIPSLTFLLDSDLETLKKSMKFYRTVFGVDETSLQRLARDWEIEVDQVEAMIKSPAVFKPTDEETIQERLSRYIQEFCLANGYLLPKNSFLKEIFYLKYYFLDMVTEDAKTLLKEICLKL

DisProt Identifier	L	Primary Sequence
>DisProt|DP00063|uniprot|P49799|unigene|Rn.11065|sp|RGS4_RAT	50	MCKGLAGLPASCLRSAKDMKHRLGFLLQKSDSCEHSSSHSKKDKVVTCQR
>DisProt|DP00246|uniprot|P21758-1|unigene|Bt.4482|sp|MSRE_BOVIN	50	MAQWDDFPDQQEDTDSCTESVKFDARSVTALLPPHPKNGPTLQERMKSYK
>DisProt|DP00449|uniprot|P53563-1|unigene|Rn.10323|sp|B2CL1_RAT	50	EENRTEAPEETEPERETPSAINGNPSWHLADSPAVNGATGHSSSLDAREV
>DisProt|DP00076|uniprot|P06786|sp|TOP2_YEAST	51	QGNDKDYIDLAFSKKKADDRKEWLRQYEPGTVLDPTLKEIPISDFINKELI
>DisProt|DP00088|uniprot|P0ABI8|sp|CYOB_ECOLI	51	MFGKLSLDAVPFHEPIVMVTIAGIILGGLALVGLITYFGKWTYLWKEWLTS
>DisProt|DP00114|uniprot|P04350|unigene|Hs.110837|sp|TBB4_HUMAN	51	FLHWYTGEGMDEMEFTEAESNMNDLVSEYQQYQDATAEQGEFEEEAEEEVA
>DisProt|DP00262|uniprot|Q16665-1|unigene|Hs.597216|sp|HIF1A_HUMAN	51	SDLACRLLGQSMDESGLPQLTSYDCEVNAPIQGSRNLLQGEELLRALDQVN
>DisProt|DP00314|uniprot|Q9UJY5-1|unigene|Hs.499158|sp|GGA1_HUMAN	51	KQGIVKSDPKLPDDTTFPLPPPRPKNVIFEDEEKSKMLARLLKSSHPEDLR
>DisProt|DP00356|uniprot|Q99967-1|unigene|Hs.82071|sp|CITE2_HUMAN	51	TDFIDEEVLMSLVIEMGLDRIKELPELWLGQNEFDFMTDFVCKQQPSRVSC
>DisProt|DP00470|trembl|Q39532|pir|S42493	51	ATSSSSRDPSPSDEDMDGEVEILGFKMPTEERVRKRKESNRESARRSRYRK
>DisProt|DP00488|sp|P49723	51	YNAVNPFEFMEDVATAGKTTFFEKKVSDYQKASDMSKSATPSKEINFDDDF
>DisProt|DP00226|uniprot|Q9CXW3|unigene|Mm.10702|sp|CYBP_MOUSE	52	EKPSYDTEADPSEGLMNVLKKIYEDGDDDMKRTINKAWVESREKQAREDTEF
>DisProt|DP00404_A001|uniprot|O00204-2|unigene|Hs.369331|sp|ST2B1_HUMAN	52	EEDGSPDPEPSPEPEPKPSLEPNTSLEREPRPNSSPSPSPGQASETPHPRPS
>DisProt|DP00061|uniprot|P27694|unigene|Hs.461925|sp|RFA1_HUMAN	54	PVPYNEGLGQPQVAPPAPAASPAASSRPQPQNGSSGMGSTVSKAYGASKTFGKA
>DisProt|DP00298|uniprot|Q07817|unigene|Hs.516966|sp|B2CL1_HUMAN	54	SDVEENRTEAPEGTESEMETPSAINGNPSWHLADSPAVNGATGHSSSLDAREVI
>DisProt|DP00377|uniprot|P45577|sp|PROQ_ECOLI	54	AATAGEKEDAPRRERKPRPTTPRRKEGAERKPRAQKPVEKAPKTVKAPREEQHT
>DisProt|DP00426|uniprot|P02730|unigene|Hs.443948|sp|B3AT_HUMAN	54	MEELQDDYEDMMEENLEQEEYEDPDIPESQMEEPAAHDTEATATDYHTTSHPGT
>DisProt|DP00558|sp|Q53XJ9	54	MAATEGVGEAAQGGEPGQPAQPPPQPHPPPPQQQHKEEMAAEAGEAVASPMDDG
>DisProt|DP00026|uniprot|P06179|sp|FLIC_SALTY	55	AQVINTNSLSLLTQNNLNKSQSALGTAIERLSSGLRINSAKDDAAGQAIANRFTA
>DisProt|DP00143|uniprot|P0A7N9|sp|RL33_ECOLI	55	MAKGIREKIKLVSSAGTGHFYTTTKNKRTKPEKLELKKFDPVVRQHVIYKEAKIK
>DisProt|DP00194|uniprot|P0AFZ3|sp|SSPB_ECOLI	55	DEDTSIMNDEEASADNETVMSVIDGDKPDHDDDTHPDDEPPQPPRGGRPALRVVK
>DisProt|DP00424|uniprot|P04325|sp|REV_HV112	55	TYLGRSAEPVPLQLPPLERLTLDCNEDCGTSGTQGVGSPQILVESPTILESGAKE
>DisProt|DP00506|uniprot|P07766|sp|P07766	55	KNRKAKAKPVTRGAGAGGRQRGQNKERPPPVPNPDYEPIRKGQRDLYSGLNQRRI
>DisProt|DP00175|uniprot|Q9NQB0|unigene|Hs.593995|sp|TF7L2_HUMAN	56	MPQLNGGGGDDLGANDELISFKDEGEQEEKSSENSSAERDLADVKSSLVNESETNQ
>DisProt|DP00249|uniprot|P63315|unigene|Bt.49083|sp|TNNC1_BOVIN	56	MFDKNADGYIDLEELKIMLQATGETITEDDIEELMKDGDKNNDGRIDYDEFLEFMK

>DisProt|DP00083|uniprot|P03069|sp|GCN4_YEAST	57	SDPAALKRARNTEAARRSRARKLQRMKQLEDKVEELLSKNYHLENEVARLKKLVGER
>DisProt|DP00084_A001|uniprot|P61244-2|unigene|Hs.285354|sp|MAX_HUMAN	57	KARSSAQLQTNYPSSDNSLYTNAKGSTISAFDGGSDSSSESEPEEPQSRKKLRMEAS
>DisProt|DP00111|uniprot|P27577|unigene|Mm.292415|sp|ETS1_MOUSE	58	GKLGGQDSFESVESYDSCDRLTQSWSSQSSFNSLQRVPSYDSFDYEDYPAALPNHKPK
>DisProt|DP00080|uniprot|P15337|unigene|Rn.90061|sp|CREB1_RAT	60	QISTIAESEDSQESVDSVTDSQKRREILSRRPSYRKILNDLSSDAPGVPRIEEEKSEEET
>DisProt|DP00500|uniprot|Q8MWS6|trembl|Q8MWS6	60	APPVLPSYIDPAKKSFAARMMEKMGWKHGEGLGVNKQGITAPLVAKKTAMSSGVIVQGAE
>DisProt|DP00501|uniprot|Q6IBA2|trembl|Q6IBA2	60	MPKSKELVSSSSSGSDSDSEVDKKLKRKKQVAPEKPVKKQKTGETSRALSSSKQSSSSRD
>DisProt|DP00121|uniprot|Q51912|sp|Q51912_PEPMA	61	VENKEETPETPETDSEEEVTIKANLIFANGSTQTAEFKGTFEKATSEAYAYADTLKKDNGE
>DisProt|DP00389|uniprot|P09983|sp|HLYAC_ECOLX	61	ALAYGSQGNLNPLINEISKIISAAGNFDVKEERAAASLLQLSGNASDFSYGRNSITLTASA
>DisProt|DP00448|uniprot|Q9Y613|unigene|Hs.95231|sp|FHOD1_HUMAN	61	SDEIMDLLVQSVTKSSPRALAARERKRSRGNRKSLRRTLKSGLGDDLVQALGLSKGPGLEV
>DisProt|DP00502|uniprot|P11912|sp|P11912	61	RKRWQNEKLGLDAGDEYEDENLYEGLNLDDCSMYEDISRGLQGTYQDVGSLNIGDVQLEKP
>DisProt|DP00014|uniprot|P05371|unigene|Rn.1780|sp|CLUS_RAT	62	TVTTHSSDSEVPSRVTEVVVKLFDSDPITVVLPEEVSKDNPKFMDTVAEKALQEYRRKSRME
>DisProt|DP00057|uniprot|P15340|sp|HSP1_CHICK	62	MARYRRSRTRSRSPRSRRRRRRSGRRRSPRRRRRYGSARRSRRSVGGRRRRYGSRRRRRRY
>DisProt|DP00090|uniprot|Q14722-2|unigene|Hs.654519|sp|KCAB1_HUMAN	62	MQVSIACTEHNLKSRNGEDRLLSKQSSTAPNVVNAARAKFRTVAIIARSLGTFTPQHHISLK
>DisProt|DP00234|uniprot|Q02020|unigene|Gga.41812|sp|FIBB_CHICK	62	ASVEYDNEEDSPQIDARAHRPLDKRQEAAPTLRPVAPPISGTGYQPRPPKQDKQAMKKGPII
>DisProt|DP00297|uniprot|P10415|unigene|Hs.150749|sp|BCL2_HUMAN	62	DAGDVGAAPPGAAPAPGIFSSQPGHTPHPAASRDPVARTSPLQTPAAPGAAAGPALSPVPPV
>DisProt|DP00433|uniprot|P03023|sp|LACI_ECOLI	62	MKPVTLYDVAEYAGVSYQTVSRVVNQASHVSAKTREKVEAAMAELNYIPNRVAQQLAGKQSL
>DisProt|DP00592|uniprot|P48539|unigene|Hs.80296|sp|P48539	62	MSERQGAGATNGKDKTSGENDGQKKVQEEFDIDMDAPETERAAVAIQSQFRKFQKKKAGSQS
>DisProt|DP00220|uniprot|P17810|unigene|Bt.12752|sp|PRPH2_BOVIN	63	RYLHTALEGMANPEDPECESEGWLLEKSVPETWKAFLESVKKLGKGNQVEAEGEDAGQAPAAG
>DisProt|DP00064|uniprot|P03607|sp|CAPSD_SBMV	64	MSGLFHHRTKPREIRAFVMATRLTKKQLAQAIQNTLPNPPRRKRRAKRRAAQVPKPTQAGVSMA
>DisProt|DP00267|uniprot|P08510-1|unigene|Dm.7088|sp|KCNAS_DROME	64	QQQQQQHQKEQLEQKEEQKKIAERKLQLREQQLQRNSLDGYGSLPKLSSQDEEGGAGHGFGGGP
>DisProt|DP00278|uniprot|P08050|unigene|Rn.10346|sp|CXA1_RAT	64	TGPLSPSKDCGSPKYAYFNGCSSPTAPLSPMSPPGYKLVTGDRNNSSCRNYNKQASEQNWANYS
>DisProt|DP00462|sp|P11157	64	MLSVRTPLATIADQQQLQLSPLKRLTLADKENTPPTLSSTRVLASKAARRIFQDSAELESKAPT
>DisProt|DP00158|uniprot|P73124|sp|P73124_SYNY3	65	MSTQQQARALMMRHHQFIKNRQQSMLSRAAAEIGVEAEKDFWTTVQGKPQSSFRTTYDRSNASLS
>DisProt|DP00216|uniprot|Q9FUM5|sp|Q9FUM5_BRANA	65	MADNKQSFQAGQAAGRAEEKGNVLMDKVKDAATAAGASAQTAGQKITEAAGGAVNLVKEKTGMNK
>DisProt|DP00468|uniprot|P25963|sp|P25963|pir|A39935	66	MFQAAERPQEWAMEGPRDGLKKERLLDDRHDSGLDSMKDEEYEQMVKELQEIRLEPQEVPRGSEPW
>DisProt|DP00557|uniprot|P32874|sp|Q00955|trembl|IGI01455632	66	IKDPQTGAPVPLRALINNVSGYVIKTEMYTEVKNAKGEWVFKSLGKPGSMHLRPIATPYPVKEWLQ
>DisProt|DP00081|uniprot|P51123|unigene|Dm.7380|sp|TAF1_DROME	67	EGSIGNGLDLTGILFGNIDSEGRLLQDDDGEGRGGTGFDAELRENIGSLSKLGLDSMLLEVIDLKEA
>DisProt|DP00463|sp|Q9Z0U4	67	CQIIHPPWEGGIRYRGLTRDQVKAINFLPVDYEIEYVCRGEREVVGPKVRKCLANGSWTDMDTPSRC
>DisProt|DP00511|uniprot|Q76LA1|unigene|Hs.695|sp|P04080	67	MMCGAPSATQPATAETQHIADQVRSQLEEKENKKFPVFKAVSFKSQVVAGTNYFIKVHVGDEDFVHL
>DisProt|DP00032|uniprot|P56206|sp|SYG_THET8	68	RITKKRYRLDHLLKEQPEEVLKRLYRAMEVEEENLHALVQAMMQAPERAGGAMTAAGVLDPASGEPGD
>DisProt|DP00586|uniprot|A6ZMN3|trembl|A6ZMN3	68	MNTDQQKVSEIFQSSKEKLQGDAKVVSDAFKKMASQDKDGKTTDADESEKHNYQEQYNKLKGAGHKKE
>DisProt|DP00042|uniprot|P02315|sp|H6_ONCMY	69	PKRKSATKGDEPARRSARLSARPVPKPAAKPKKAAAPKKAVKGKKAAENGDAKAEAKVQAAGDGAGNAK
>DisProt|DP00345|uniprot|P16535|sp|LKA1A_PASHA	70	GNGKITQDELSKVVDNYELLKHSKNVTNSLDKLISSVSAFTSSNDSRNVLVAPTSMLDQSLSSLQFARAA
>DisProt|DP00343|uniprot|Q9Y6Q9-3|unigene|Hs.592142|sp|NCOA3_HUMAN	71	GTQNRPLLRNSLDDLVGPPSNLEGQSDERALLDQLHTLLSNTDATGLEEIDRALGIPELVNQGQALEPKQD
>DisProt|DP00055|uniprot|P10688|unigene|Rn.12324|sp|PLCD1_RAT	72	MDQRQKLQHWIHSCLRKADKNKDNKMNFKELKDFLKELNIQVDDGYARKIFRECDHSQTDSLEDEEIETFYK
>DisProt|DP00048|uniprot|P03406|sp|NEF_HV1BR	73	MGGKWSKSSVVGWPTVRERMRRAEPAADGVGAASRDLEKHGAITSSNTAATNAACAWLEAQEEEEVGFPVTPQ

>DisProt|DP00086|uniprot|P04637|unigene|Hs.654481|sp|P53_HUMAN	73	MEEPQSDPSVEPPLSQETFSDLWKLLPENNVLSPLPSQAMDDLMLSPDDIEQWFTEDPGPDEAPRMPEAAPPV
>DisProt|DP00288|uniprot|Q06253|sp|PHD_BPP1	73	MQSINFRTARGNLSEVLNNVEAGEEVEITRRGREPAVIVSKATFEAYKKAALDAEFASLFDTLDSTNKELVNR
>DisProt|DP00015|uniprot|P61926|sp|IPKA_RABIT	75	MTDVETTYADFIASGRTGRRNAIHDILVSSASGNSNELALKLAGLDINKTEGEEDAQRSSTEQSGEAQGEAAKSE
>DisProt|DP00146|uniprot|P0A7T7|sp|RS18_ECOLI	75	MARYFRRRKFCRFTAEGVQEIDYKDIATLKNYITESGKIVPSRITGTRAKYQRQLARAIKRARYLSLLPYTDRHQ
>DisProt|DP00230|uniprot|Q14449|unigene|Hs.411881|sp|GRB14_HUMAN	75	SSQSISPMRSISENSLVAMDFSGQKSRVIENPTEALSVAVEEGLAWRKKGCLRLGTHGSPTASSQSSATNMAIHR
>DisProt|DP00490|uniprot|O88900|unigene|Rn.30028|sp|GRB14_RAT	75	QSVSPMRSVSENSLVAMDFSGQKTRVIDNPTEALSVAVEEGLAWRKKGCLRLGNHGSPTAPSQSSAVNMALHRSQ
>DisProt|DP00021|uniprot|P13551|sp|EFG_THETH	76	ESIEVPEPVIDVAIEPKTKADQEKLSQALARLAEEDPTFRVSTHPETGQTIISGMGELHLEIIVDRLKREFKVDAN
>DisProt|DP00201|uniprot|P02721|unigene|Bt.404|sp|ATP5J_BOVIN	76	NKELDPVQKLFVDKIREYRTKRQTSGGPVDAGPEYQQDLDRELFKLKQMYGKADMNTFPNFTFEDPKFEVVEKPQS
>DisProt|DP00572|sp|P16860	76	DPQTAPSRALLLLLFLHLAFLGGRSHPLGSPGSASDLETSGLQEQRNHLQGKLSELQVEQTSLEPLQESPRPTGVW
>DisProt|DP00180|uniprot|P19972|sp|TOXK_PICFA	77	GEATTIWGVGADEAIDKGTPSKNDLQNMSADLAKNGFKGHQGVACSTVKDGNKDVYMIKFSLAGGSNDPGGSPCSDD
>DisProt|DP00394|uniprot|P04189|sp|SUBT_BACSU	77	AGKSSTEKKYIVGFKQTMSAMSSAKKKDVISEKGGKVQKQFKYVNAAAATLDEKAVKELKKDPSVAYVEEDHIAHEY
>DisProt|DP00469|uniprot|O24646|sp|O24646|pir|T50922	77	MQEQATSSLAASSLPSSSERSSSSAPHLEIKEGIESDEEIRRVPEFGGEAVGKETSGRESGSATGQERTQATVGESQ
>DisProt|DP00223|uniprot|P14635|unigene|Hs.23960|sp|CCNB1_HUMAN	78	MALRVTRNSKINAENKAKINMAGAKRVPTAPAATSKPGLRPRTALGDIGNKVSEQLQAKMPMKKEAKPSATGKVIDKK
>DisProt|DP00400|uniprot|P60752|sp|MSBA_ECOLI	78	DVEFRNVTFTYPGRDVPALRNINLKIPAGKTVALVGRSGSGKSTIASLITRFYDIDEGEILMDGHDLREYTLASLRNQ
>DisProt|DP00359|uniprot|A6Q0K5|sp|CP12_CHLRE	80	SGQPAVDLNKKVQDAVKEAEDACAKGTSADCAVAWDTVEELSAAVSHKKDAVKADVTLTDPLEAFCKDAPDADECRVYED
>DisProt|DP00217|uniprot|P05221|unigene|Xl.1262|sp|NUPL_XENLA	81	MEEDYSWAEEEDEGEAEGEEEEEEEEDQESPPKAVKRPAATKKAGQAKKKKLDKEDESSEEDSPTKKGKGAGRGRKPAAKK
>DisProt|DP00156|uniprot|P54725|unigene|Hs.643267|sp|RD23A_HUMAN	82	TKAGQGTSAPPEASPTAAPESSTSFPPAPTSGMSHPPPAAREDKSPSEESAPTTSPESVSGSVPSSGSSGREEDAASTLVTG
>DisProt|DP00423|uniprot|P53551|sp|H1_YEAST	82	KASSPSSLTYKEMILKSMPQLNDGKGSSRIVLKKYVKDTFSSKLKTSSNFDYLFNSAIKKCVENGELVQPKGPSGIIKLNKK
>DisProt|DP00510|sp|O60356	82	MATFPPATSAPQQPPGPEDEDSSLDESDLYSLAHSYLGGGGRKGRTKREAAANTNRPSPGGHERKLVTKLQNSERKKRGARR
>DisProt|DP00588	82	MSTNPKPQRKTKRNTNRRPQDVKFPGGGQIVGGVYLLPRRGPRLGVRATRKTSERSQPRGRRQPIPKARQPEGRAWAQPGYP
>DisProt|DP00177|uniprot|Q9Y5B0|unigene|Hs.465490|sp|CTDP1_HUMAN	83	PEEQEEEPQPRKPGTRRGADARAPASSERSAAGGRGPRGHKRKLNEEDAASESSRESSNEDEGSSSEADEMAKALEAELNDLM
>DisProt|DP00229|uniprot|P05455|unigene|Hs.632535|sp|LA_HUMAN	83	LNKWKSKGRRFKGKGKGNKAAQPGSGKGKVQFQGKKTKFASDDEHDEHDENGATGPVKRAREETDKEEPASKQQKTENGAGDQ
>DisProt|DP00242|uniprot|P0AG63|sp|RS17_ECOLI	83	TDKIRTLQGRVVSDKMEKSIVVAIERFVKHPIYGKFIKRTTKLHVHDENNECGIGDVVEIRECRPLSKTKSWTLVRVVEKAVL
>DisProt|DP00342|uniprot|P09883|sp|CEA9_ECOLX	83	MSGGDGRGHNTGAHSTSGNINGGPTGIGVSGGASDGSGWSSENNPWGGGSGSGIHWGGGSGRGNGGGNGNSGGGSGTGGNLSA
>DisProt|DP00091|uniprot|P27088|unigene|Xl.902|sp|XPA_XENLA	84	MEPEPEPEQEANKEEEKILSAAVRAKIERNRQRALMLRQARLACRPYPTGEGISTVKAPPKVIDSGGGFFIEEEEAEEQHVENV
>DisProt|DP00306|uniprot|Q07108|unigene|Hs.208854|sp|CD69_HUMAN	84	MSSENCFVAENSSLHPESGQENDATSPHFSTRHEGSFQVPVLCAVMNVVFITILIIALIALSVGQYNCPGQYTFSMPSDSHVSS
>DisProt|DP00018|uniprot|P46527|unigene|Hs.238990|sp|CDN1B_HUMAN	85	EHPKPSACRNLFGPVDHEELTRDLEKHCRDMEEASQRKWNFDFQNHKPLEGKYEWQEVEKGSLPEFYYRPPRPPKGACKVPAQES
>DisProt|DP00044|uniprot|P02259|unigene|Gga.16744|sp|H5_CHICK	85	AKRSPGKKKKAVRRSTSPKKAARPRKARSPAKKPKATARKARKKSRASPKKAKKPKTVKAKSRKASKAKKVKRSKPRAKSGARKS
>DisProt|DP00053|uniprot|P27001|sp|SYFA_THETH	85	MLEEALAAIQNARDLEELKALKARYLGKKGLLTQEMKGLSALPLEERRKRGQELNAIKAALEAALEAREKALEEAALKEALERER
>DisProt|DP00091|uniprot|P27088|unigene|Xl.902|sp|XPA_XENLA	85	IKRSLEVWGSEEALEEAKEVRKDNRDKMKQKKFDKKVKELRRTVRSSLWKKEASGHQHEYGPEEHVEEDSYKKTCITCGYEMNYE
>DisProt|DP00140|uniprot|P0A7L8|sp|RL27_ECOLI	85	MAHKKAGGSTRNGRDSEAKRLGVKRFGGESVLAGSIIVRQRGTKFHAGANVGCGRDHTLFAKADGKVKFEVKGPKNRKFISIEAE
>DisProt|DP00499|uniprot|Q9Z2F5|unigene|Rn.3946|sp|Q9Z2F5	86	LTAATHWASMDPAVVHPELNGAAYSRYPPGVVSVAPTGIPAAVEGIVPSAMSLSHGLPPVAHPPHAPSPGQTVKPEADRDHTTDQL
>DisProt|DP00152|uniprot|Q13426|unigene|Hs.567359|sp|XRCC4_HUMAN	87	RFILVLNEKKTKIRSLHNKLLNAAQEREKDIKQEGETAICSEMTADRDPVYDESTDEESENQTDLSGLASAAVSKDDSIISSLDVTD
>DisProt|DP00347|uniprot|P04972|unigene|Bt.54|sp|CNRG_BOVIN	87	MNLEPPKAEIRSATRVMGGPVTPRKGPPKFKQRQTRQFKSKPPKKGVQGFGDDIPGMEGLGTDITVICPWEAFNHLELHELAQYGII
>DisProt|DP00087|uniprot|P68336|sp|VP16_HHV2H	88	AGQRPRRLSTTAPITDVSLGDELRLDGEEVDMTPADALDDFDLEMLGDVESPSPGMTHDPVSYGALDVDDFEFEQMFTDAMGIDDFGG
>DisProt|DP00157|uniprot|Q56027|sp|SIPA_SALTY	88	GETTSFDEVDGVTSKSIIGKPVQATVHGVDDNKQQSQTAEIVNVKPLASQLAGVENVKTDTLQSDTTVITGNKAGTTDNDNSQTDKTG

<table>
<tr><td>>DisProt|DP00039|uniprot|P05204|unigene|Hs.181163|sp|HMGN2_HUMAN</td><td>89</td><td>PKRKAEGDAKGDKAKVKDEPQRRSARLSAKPAPPKPEPKPKKAPAKKGEKVPKGKKGKADAGKEGNNPAENGDAKTDQAQKAEGAGDAK</td></tr>
<tr><td>>DisProt|DP00141|uniprot|O95405-1|unigene|Hs.532345|sp|ZFYV9_HUMAN</td><td>89</td><td>NMMSASSQSPNPNNPAEYCSTIPPLQQAQASGALSSPPPTVMVPVGVLKHPGAEVAQPREQRRVWFADGILPNGEVADAAKLTMNGTSS</td></tr>
<tr><td>>DisProt|DP00195|uniprot|P02313|unigene|Bt.1758|sp|HMGN2_BOVIN</td><td>89</td><td>PKRKAEGDAKGDKAKVKDEPQRRSARLSAKPAPPKPEPKPKKAPAKKGEKVPKGKKGKADAGKBGBBPAZBGBAKTBZAZKAEGAGDAK</td></tr>
<tr><td>>DisProt|DP00353|uniprot|P23202|sp|URE2_YEAST</td><td>90</td><td>MMNNNGNQVSNLSNALRQVNIGSRNSNTTTDQSNINFEFSTGVNNNNNNNSSSNNNNVQNNNSGRNGSQNNDNENNIKNTLEQHRQQQQA</td></tr>
<tr><td>>DisProt|DP00486|sp|Q9UBT2</td><td>90</td><td>PEKVGPKQAEDAAKSITNGSDDGAQPSTSTAQEQDDVLIVDSDEEDSSNNADVSEEERSRKRKLDEKENLSAKRSRIEQKEELDDVIALD</td></tr>
<tr><td>>DisProt|DP00527|sp|P02783|trembl|P02783</td><td>91</td><td>RKTKEKYSQSEEVVSESFASGPSSGSSDDELVRDKPYGPKVSGGSFGEEASEEISSRRSKHISRSSGGSNMEGESSYAKKKRSRFAQDVLN</td></tr>
<tr><td>>DisProt|DP00022|uniprot|P17639|sp|EMB1_DAUCA</td><td>92</td><td>MASQQEKKELDARARQGETVVPGGTGGKSLEAQQHLAEGRSKGGQTRKEQLGGEGYHEMGRKGGLSNNDMSGGERAEQEGIDIDESKFRTKK</td></tr>
<tr><td>>DisProt|DP00131|uniprot|Q91006|unigene|Gga.2614|sp|Q91006_CHICK</td><td>92</td><td>MSYRKELEKYRDLDEDKILGALTEEELRKLENELEELDPDNALLPAGLRQRDQTQKPPTGPFKREELMAHLEQQAKDIKDREDLVPFTGEKR</td></tr>
<tr><td>>DisProt|DP00147|uniprot|P0A7U3|sp|RS19_ECOLI</td><td>92</td><td>MPRSLKKGPFIDLHLLKKVEKAVESGDKKPLRTWSRRSTIFPNMIGLTIAVHNGRQHVPVFVTDEMVGHKLGEFAPTRTYRGHAADKKAKKK</td></tr>
<tr><td>>DisProt|DP00113|uniprot|P31109|sp|SNC1_YEAST</td><td>93</td><td>MSSSTPFDPYALSEHDEERPQNVQSKSRTAELQAEIDDTVGIMRDNINKVAERGERLTSIEDKADNLAVSAQGFKRGANRVRKAMWYKDLKMK</td></tr>
<tr><td>>DisProt|DP00348|uniprot|P45481|unigene|Mm.132238|sp|CBP_MOUSE</td><td>94</td><td>PNRSISPSALQDLLRTLKSPSSPQQQQQVLNILKSNPQLMAAFIKQRTAKYVANQPGMQPQPGLQSQPGMQPQPGMHQQPSLQNLNAMQAGVPR</td></tr>
<tr><td>>DisProt|DP00334|uniprot|Q00987-1|unigene|Hs.484551|sp|MDM2_HUMAN</td><td>95</td><td>SSSSESTGTPSNPDLDAGVSEHSGDWLDQDSVSDQFSVEFEVESLDSEDYSLSEEGQELSDEDDEVYQVTVYQAGESDTDSFEEDPEISLADYWK</td></tr>
<tr><td>>DisProt|DP00187|uniprot|P04273|sp|PRIO_MESAU</td><td>96</td><td>GGWNTGGSRYPGQGSPGGNRYPPQGGGTWGQPHGGGWGQPHGGGWGQPHGGGWGQPHGGGWGQGGGTHNQWNKPSKPKTNMKHMAGAAAAGAVVGG</td></tr>
<tr><td>>DisProt|DP00517</td><td>96</td><td>GGGVSPDVCPALSDEPGGLTASRGRVHEASPPTFQKDALLGSKPNKPSLPSSSQNLGQTEVSKVSETVQEELTPPPQKAAPQGKSKSDPLKKKTDR</td></tr>
<tr><td>>DisProt|DP00552|uniprot|P0AFX7|sp|P0AFX7</td><td>96</td><td>YNGQSETSQQPETPVFNTLPMMGKASPVSLGVPSEATANNGQQQQVQEQRRRINAMLQDYELQRRLHSEQLQFEQAQTQQAAVQVPGIQTLGTQSQ</td></tr>
<tr><td>>DisProt|DP00027|uniprot|P26477|sp|FLGM_SALTY</td><td>97</td><td>MSIDRTSPLKPVSTVQTRETSDTPVQKTRQEKTSAATSASVTLSDAQAKLMQPGVSDINMERVEALKTAIRNGELKMDTGKIADSLIREAQSYLQSK</td></tr>
<tr><td>>DisProt|DP00491|sp|Q62165</td><td>97</td><td>VVEWTNNTLPLEPCPKEQIIGLSRRIADENGKPRPAFSNALEPDFKALSIAVTGSGSCRHLQFIPVAPPSPGSSAAPATEVPDRDPEKSSEDDVYLH</td></tr>
<tr><td>>DisProt|DP00024|uniprot|P03129|sp|VE7_HPV16</td><td>98</td><td>MHGDTPTLHEYMLDLQPETTDLYCYEQLSDSSEEEDEIDGPAGQAEPDRAHYNIVTFCCKCDSTLRLCVQSTHVDIRTLEDLLMGTLGIVCPICSQKP</td></tr>
<tr><td>>DisProt|DP00082|uniprot|P39935|sp|IF4F1_YEAST</td><td>98</td><td>LEAEIETTTDETDDGTNTVSHILNVLKDATPIEDVFSFNYPEGIEGPDIKYKKEHVKYTYGPTFLLQFKDKLNVKADAEWVQSTASKIVIPPGMGRGN</td></tr>
<tr><td>>DisProt|DP00097|uniprot|P10922|unigene|Mm.24350|sp|H10_MOUSE</td><td>98</td><td>KGDEPKRSVAFKKTKKEVKKVATPKKAAKPKKAASKAPSKKPKATPVKKAKKKPAATPKKAKKPKVVKVKPVKASKPKKAKTVKPKAKSSAKRASKKK</td></tr>
<tr><td>>DisProt|DP00265|uniprot|P04925|unigene|Mm.648|sp|PRIO_MOUSE</td><td>98</td><td>KKRPKPGGWNTGGSRYPGQGSPGGNRYPPQGGTWGQPHGGGWGQPHGGSWGQPHGGSWGQPHGGGWGQGGGTHNQWNKPSKPKTNLKHVAGAAAAGAV</td></tr>
<tr><td>>DisProt|DP00038|uniprot|P02316|unigene|Bt.87121|sp|HMGN1_BOVIN</td><td>99</td><td>PKRKVSSAEGAAKEEPKRRSARLSAKPAPAKVETKPKKAAGKDKSSDKKVQTKGKRGAKGKQAEVANQETKEDLPAENGETKNEESPASDEAEEKEAKS</td></tr>
<tr><td>>DisProt|DP00095|uniprot|P00952|sp|SYY_BACST</td><td>99</td><td>ALFSGDIANLTAAEIEQGFKDVPSFVHEGGDVPLVELLVSAGISPSKRQAREDIQNGAIYVNGERLQDVGAILTAEHRLEGRFTVIRRGKKKYYLIRYA</td></tr>
<tr><td>>DisProt|DP00447|uniprot|P12579|sp|PHOSP_HRSVL</td><td>99</td><td>MEKFAPEFHGEDANNRATKFLESIKGKFTSPKDPKKKDSIISVNSIDIEVTKESPITSNSTIINPTNETDDTVGNKPNYQRKPLVSFKEDPTPSDNPFS</td></tr>
<tr><td>>DisProt|DP00465|sp|Q57696</td><td>99</td><td>MIEKLAEIRKKIDEIDNKILKLIAERNSLAKDVAEIKNQLGIPINDPEREKYIYDRIRKLCKEHNVDENIGIKIFQILIEHNKALQKQYLEETQNKNKK</td></tr>
<tr><td>>DisProt|DP00466|sp|P04156</td><td>99</td><td>KKRPKPGGWNTGGSRYPGQGSPGGNRYPPQGGGGWGQPHGGGWGQPHGGGWGQPHGGGWGQPHGGGWGQGGGTHSQWNKPSKPKTNMKHMAGAAAAGAV</td></tr>
<tr><td>>DisProt|DP00401|uniprot|P52477|sp|MEXA_PSEAE</td><td>101</td><td>QEGVKQKAILAPQQGVTRDLKGQATALVVNAQNKVELRVIKADRVIGDKWLVTEGLNAGDKIITEGLQFVQPGVEVKTVPAKNVASAQKADAAPAKTDSKG</td></tr>
<tr><td>>DisProt|DP00458|sp|P37727</td><td>101</td><td>LHKDVEEAGALQKNHASVTSAQSAEAAEAAETSCLPTAVEPLSMGSCEIPAEQSQCPGPESSPEVNDAEATGKKENSDAKSSTEEPSENVPKVQDNTETPK</td></tr>
<tr><td>>DisProt|DP00046|uniprot|Q9QXN1|unigene|Rn.21926|sp|LEF1_RAT</td><td>102</td><td>HIKKPLNAFMLYMKEMRANVVAECTLKESAAINQILGRRWHALSREEQAKYYELARKERQLHMQLYPGWSARDNYGKKKKRKREKLQESTSGTGPRMTAAYI</td></tr>
<tr><td>>DisProt|DP00551|uniprot|P16860|unigene|Hs.219140|sp|P16860</td><td>102</td><td>MDPQTAPSRALLLLLFLHLAFLGGRSHPLGSPGSASDLETSGLQEQRNHLQGKLSELQVEQTSLEPLQESPRPTGVWKSREVATEGIRGHRKMVLYTLRAPR</td></tr>
<tr><td>>DisProt|DP00079|uniprot|Q92731-1|unigene|Hs.525392|sp|ESR2_HUMAN</td><td>103</td><td>MDIKNSPSSLNSPSSYNCSQSILPLEHGSIYIPSSYVDSHHEYPAMTFYSPAVMNYSIPSNVTNLEGGPGRQTTSPNVLWPTPGHLSPLVVHRQLSHLYAEPQ</td></tr>
<tr><td>>DisProt|DP00473|uniprot|Q8IV71|trembl|Q8IV71</td><td>103</td><td>NHAETSRLNIERMKQIYQQLSRYRGLCLLASVPSCVEDLSHPVAEPSSSQAGSMSSAGPRPLPSGPASPKRKLEAAEEPPGEELSKRARVAELPTPELPSKDA</td></tr>
<tr><td>>DisProt|DP00532|uniprot|Q8GT36|sp|Q8GT36|trembl|Q8GT36</td><td>103</td><td>MSSLPFVFGAAASSRVVTAAAAKGTAETKQEKSFVDWLLGKITKEDQFYETDPILRGGDVKSSGSTSGKKGGTTSGKKGTVSIPSKKKNGNGGVFGGLFAKKD</td></tr>
<tr><td>>DisProt|DP00006|uniprot|P00004|unigene|Eca.1571|sp|CYC_HORSE</td><td>104</td><td>GDVEKGKKIFVQKCAQCHTVEKGGKHKTGPNLHGLFGRKTGQAPGFTYTDANKNKGITWKEETLMEYLENPKKYIPGTKMIFAGIKKKTEREDLIAYLKKATNE</td></tr>
<tr><td>>DisProt|DP00075|uniprot|P11387|unigene|Hs.472737|sp|TOP1_HUMAN</td><td>105</td><td>NRANRAVAILCNHQRAPPKTFEKSMMNLQTKIDAKKEQLADARRDLKSAKADAKVMKDAKTKKVVESKKKAVQRLEEQLMKLEVQATDREENKQIALGTSKLNYL</td></tr>
<tr><td>>DisProt|DP00185|uniprot|P93165|unigene|Gma.10|sp|P93165_SOYBN</td><td>105</td><td>MASRQNNKQELDERARQGETVVPGGTGGKSLEAQQHLAEGRSKGGQTRKEQLGTEGYQEMGRKGGLSTVDKSGEERAQEEGIGIDESKFRTGNNKNQNQNEDQDK</td></tr>
<tr><td>>DisProt|DP00066|uniprot|P27285|sp|POLS_SINDO</td><td>106</td><td>MNRGFFNMLGRRPFPAPTAMWRPRRRRQAAPMPARNGLASQIQQLTTAVSALVIGQATRPQNPRPRPPPRQKKQAPKQPPKPKKPKPQEKKKKQPAKTKPGKRQRM</td></tr>
</table>

>DisProt|DP00164|uniprot|P05318|sp|RLA1_YEAST	106	MSTESALSYAALILADSEIEISSEKLLTLTNAANVPDENIWADIFAKALDGQNLKDLLVNFSAGAAAPAGVAGGVAGGEAGEAEAEKEEEEAKEESDDDMGFGLFD
>DisProt|DP00372|uniprot|Q9NR00|unigene|Hs.591849|sp|CH004_HUMAN	106	MKAKRSHQAIIMSTSLRVSPSIHGYHFDTASRKKAVGNIFENTDQESLERLFRNSGDKKAEERAKIIFAIDQDVEEKTRALMALKKRTKDKLFQFLKLRKYSIKVH
>DisProt|DP00005|uniprot|P03045|sp|REGN_LAMBD	107	MDAQTRRRERRAEKQAQWKAANPLLVGVSAKPVNLPILSLNRKPKSRVESALNPIDLTVLAEYHKQIESNLQRIERKNQRTWYSKPGERGITCSGRQKIKGKSIPLI
>DisProt|DP00040|uniprot|P17096|unigene|Hs.518805|sp|HMGA1_HUMAN	107	MSESSSKSSQPLASKQEKDGTEKRGRGRPRKQPPVSPGTALVGSQKEPSEVPTPKRPRGRPKGSKNKGAAKTRKTTTTPGRKPRGRPKKLEKEEEEGISQESSEEEQ
>DisProt|DP00001|uniprot|Q9HFQ6|sp|RLA3_CANAL	108	MSTEASVSYAALILADAEQEITSEKLLAITKAAGANVDQVWADVFAKAVEGKNLKELLFSFAAAAPASGAAAGSASGAAAGGEAAAEEAAEEEAAEESDDDMGFGLFD
>DisProt|DP00550|uniprot|P02628|sp|P02628	108	AKDLLKADDIKKALDAVKAEGSFNHKKFFALVGLKAMSANDVKKVFKAIDADASGFIEEEELKFVLKSFAADGRDLTDAETKAFLKAADKDGDGKIGIDEFETLVHEA
>DisProt|DP00002|uniprot|P02400|sp|RLA4_YEAST	110	MKYLAAYLLLVQGGNAAPSAADIKAVVESVGAEVDEARINELLSSLEGKGSLEEIIAEGQKKFATVPTGGASSAAAGAAGAAAGGDAAEEEKEEEAKEESDDDMGFGLFD
>DisProt|DP00084|uniprot|P61244-1|unigene|Hs.285354|sp|MAX_HUMAN	110	MSDNDDIEVESDEEQPRFQSAADKRAHHNALERKRRDHIKDSFHSLRDSVPSLQGEKASRAQILDKATEYIQYMRRKNHTHQQDIDDLKRQNALLEQQVRALEKARSSAQ
>DisProt|DP00256|uniprot|P40316|sp|SECU_YEAST	110	MMPANEDKENNIVYTGNESSGINFPQTPAHLLKRSHSNILKPPVRLDQLKRDANSNNGNTLKYIQGGKEVSPTKRLHTHAQQQGRLPLAAKDNNRSKSFIFPETSNQSKD
>DisProt|DP00088|uniprot|P0ABI8|sp|CYOB_ECOLI	111	AVVPHVHERDAFWEMKEKGEAYKKPDHYEEIHMPKNSGAGIVIAAFSTIFGFAMIWHIWWLAIVGFAGMIITWIVKSFDEDVDYYVPVAEIEKLENQHFDEITKAGLKNGN
>DisProt|DP00514|uniprot|P34945|sp|P34945	111	MVDLKRLRQEPEVFHRAIREKGVALDLEALLALDREVQELKKRLQEVQTERNQVAKRVPKAPPEEKEALIARGKALGEEAKRLEEALREKEARLEALLLQVPLPPWPGAPV
>DisProt|DP00058|uniprot|P06302|unigene|Rn.817|sp|PTMA_RAT	112	MSDAAVDTSSEITTKDLKEKKEVVEEAENGRDAPANGNAQNEENGEQEADNEVDEEEEEGGEEEEEEEEGDGEEEDGDEDEEAEAPTGKRVAEDDEDDDVETKKQKKTDEDD
>DisProt|DP00200|uniprot|P20963-1|unigene|Hs.156445|sp|CD3Z_HUMAN	113	RVKFSRSADAPAYQQGQNQLYNELNLGRREEYDVLDKRRGRDPEMGGKPQRRKNPQEGLYNELQKDKMAEAYSEIGMKGERRRGKGHDGLYQGLSTATKDTYDALHMQALPPR
>DisProt|DP00518|uniprot|O54918|unigene|Mm.141083|sp|B2L11_MOUSE	113	MAKQPSDVSSECDREGGQLQPAERPPQLRPGAPTSLQTEPQDRSPAPMSCDKSTQTPSPPCQAFNHYLSAMASIRQSQEEPEDLRPEIRIAQELRRIGDEFNETYTRRVFAND
>DisProt|DP00531|uniprot|Q08655|unigene|Les.17636|sp|Q08655	115	MEEEKHHHHHLFHHKDKAEEGPVDYEKEIKHHKHLEQIGKLGTVAAGAYALHEKHEAKKDPEHAHKHKIEEEIAAAAAVGAGGFAFHEHHEKKDAKKEEKKKLRGDTTISSKLLF
>DisProt|DP00069|uniprot|P63027|unigene|Hs.25348|sp|VAMP2_HUMAN	116	MSATAATAPPAAPAGEGGPPAPPPNLTSNRRLQQTQAQVDEVVDIMRVNVDKVLERDQKLSELDDRADALQAGASQFETSAAKLKRKYWWKNLKMMIILGVICAIILIIIIVYFSS
>DisProt|DP00173|uniprot|O00273-1|unigene|Hs.484782|sp|DFFA_HUMAN	116	MEVTGDAGVPESGEIRTLKPCLLRRNYSREQHGVAASCLEDLRSKACDILAIDKSLTPVTLVLAEDGTIVDDDDYFLCLPSNTKFVALASNEKWAYNNSDGGTAWISQESFDVDET
>DisProt|DP00387|uniprot|P25814|sp|RNPA_BACSU	116	MKKRNRLKKNEDFQKVFKHGTSVANRQFVLYTLDQPENDELRVGLSVSKKIGNAVMRNRIKRLIRQAFLEEKERLKEKDYIIIARKPASQLTYEETKKSLQHLFRKSSLYKKSSSK
>DisProt|DP00134|uniprot|Q06787|unigene|Hs.103183|sp|FMR1_HUMAN	117	APTEEERESFLRRGDGRRRGGGGRGQGGRGRGGGFKGNDDHSRTDNRPRNPREAKGRTTDGSLQIRVDCNNERSVHTKTLQNTSSEGSRLRTGKDRNQKKEKPDSVDGQQPLVNGVP
>DisProt|DP00205|uniprot|Q82S91|sp|SMBP_NITEU	117	MKTTLIKVIAASVTALFLSMQVYASGHTAHVDEAVKHAEEAVAHGKEGHTDQLLEHAKESLTHAKAASEAGGNTHVGHGIKHLEDAIKHGEEGHVGVATKHAQEAIEHLRASEHKSH
>DisProt|DP00028|uniprot|Q13541|unigene|Hs.411641|sp|4EBP1_HUMAN	118	MSGGSSCSQTPSRAIPATRRVVLGDGVQLPPGDYSTTPGGTLFSTTPGGTRIIYDRKFLMECRNSPVTKTPPRDLPTIPGVTSPSSDEPPMEASQSHLRNSPEDKRAGGEESQFEMDI
>DisProt|DP00538|uniprot|A8CDV5|trembl|A8CDV5	118	MGSLEMVPMGAGPPSPGGDPDGDDGGNNSQYPSASGSSGNTPTPPNDEERESNEEPPPPYEDLDWGNGDRHSDYQPLGNQDPSLYLGLQHDGNDGLPPPPYSPRDDSSQHIYEEAGRG
>DisProt|DP00553|uniprot|Q9NZ94|unigene|Hs.438877|sp|Q9NZ94	118	YRKDKRRQEPLRQPSPQRGAGAPELGAAPEEELAALQLGPTHHECEAGPPHDTLRLTALPDYTLTLRRSPDDIPLMTPNTITMIPNSLVGLQTLHPYNTFAAGFNSTGLPHSHSTTRV
>DisProt|DP00537|uniprot|O43236|unigene|Hs.287518|sp|O43236|trembl|O43236	119	MDRSLGWQGNSVPEDRTEAGIKRFLEDTTDDGELSKFVKDFSGNASCHPPEAKTWASRPQVPEPRPQAPDLYDDDLEFRPPSRPQSSDNQQYFCAPAPLSPSARPRSPWGKLDPYDSSE
>DisProt|DP00049|uniprot|P70475|unigene|Rn.10559|sp|MYT1L_RAT	120	HVKKPYYDPSRTEKRESKCPTPGCDGTGHVNGLYPHHRSLSGCPHKDRVPPEILAMHENVLKCPTPGCTGRGHVNSNRNSHRSLSGCPIAAAEKLAKAQEKHQSCDVSKSNQASDRVLRP
>DisProt|DP00399|uniprot|P50097|sp|IMDH_TRIFO	120	GFVVSDSNVKPDQTFADVLAISQRTTHNTVAVTDDGTPHGVLLGLVTQRDYPIDLTQTETKVSDMMTPFSKLVTAHQDTKLSEANKIIWEKKLNALPIIDDDQHLRYIVFRKDYDRSQVC
>DisProt|DP00544	120	MAKSIRSKHRRQMRNVKREHFAKKDLDRLKRLASKAQELDLDNVVTMKSAEEIKNKPSTSASDADKGMEVDNTKKVFKKKTQQNEDGHYPQWMNQRAVKKQKVKVAKLKTKKKIGKKIKW
>DisProt|DP00085|uniprot|Q04206|unigene|Hs.502875|sp|TF65_HUMAN	123	TQAGEGTLSEALLQLQFDDEDLGALLGNSTDPAVFTDLASVDNSEFQQLLNQGIPVAPHTTEPMLMEYPEAITRLVTGAQRPPDPAPAPLGAPGLPNGLLSGDEDFSSIADMDFSALLSQISS
>DisProt|DP00012|uniprot|P13569|unigene|Hs.489786|sp|CFTR_HUMAN	124	IRKFSIVQKTPLQMNGIEEDSDEPLERRLSLVPDSEQGEAILPRISVISTGPTLQARRRQSVLNLMTHSVNQGQNIHRKTTASTRKVSLAPQANLTELDIYSRRLSQETGLEISEEINEEDLKE
>DisProt|DP00145|uniprot|P0A7S3|sp|RS12_ECOLI	124	MATVNQLVRKPRARKVAKSNVPALEACPQKRGVCTRVYTTTPKKPNSALRKVCRVRLTNGFEVTSYIGGEGHNLQEHSVILIRGGRVKDLPGVRYHTVRGALDCSGVKDRKQARSKYGVKRPKA
>DisProt|DP00418|uniprot|P78504|unigene|Hs.224012|sp|JAG1_HUMAN	125	RKRRKPGSHTHSASEDNTTNNVREQLNQIKNPIEKHGANTVPIKDYENKNSKMSKIRTHNSEVEEDDMDKHQQKARFAKQPAYTLVDREEKPPNGTPTKHPNWTNKQDNRDLESAQSLNRMEYIV
>DisProt|DP00160|uniprot|P04851|sp|NCAP_MEASE	126	HTTEDKISRAVGPRQAQVSFLHGDQSENELPRLGGKEDRRVKQSRGEARESYRETGPSRASDARAAHLPTGTPLDIDTASESSQDPQDSRRSADALLRLQAMAGISEEQGSDTDTPIVYNDRNLLD
>DisProt|DP00219|uniprot|O60927|unigene|Hs.82887|sp|PP1RB_HUMAN	126	MAEAGAGLSETVTETTVTVTTEPENRSLTIKLRKRKPEKKVEWTSDTVDNEHMGRRSSKCCCIYEKPRAFGESSTESDEEEEEGCGHTHCVRGHRKGRRRATLGPTPTTPPQPPDPSQPPPGPMQH
>DisProt|DP00025|uniprot|P14738|sp|FNBA_STAA8	129	GQNSGNQSFEEDTEEDKPKYEQGGNIVDIDFDSVPQIHGQNKGNQSFEEDTEKDKPKYEHGGNIIDIDFDSVPHIHGFNKHTEIIEEDTNKDKPSYQFGGHNSVDFEEDTLPKVSGQNEGQQTIEEDTT
>DisProt|DP00365|uniprot|P53041|sp|P53041|pir|S52570	129	PPADGALKRAEELKTQANDYFKAKDYENAIKFYSQAIELNPSNAIYYGNRSLAYLRTECYGYALGDATRAIELDKKYIKGYYRRAASNMALGKFRAALRDYETVVKVKPHDKDAKMKYQECNKIVKQKA

>DisProt|DP00224|uniprot|P15884|unigene|Hs.605153|sp|ITF2_HUMAN	130	MHHQQRMAALGTDKELSDLLDFSAMFSPPVSSGKNGPTSLASGHFTGSNVEDRSSSGSWGNGGHPSPSRNYGDGTPYDHMTSRDLGSHDNLSPPFVNSRIQSKTERGSYSSYGRESNLQGCHQQSLLGGD
>DisProt|DP00534|sp|Q8LC81|trembl|Q8LC81	131	MATIATGLNIATQRVFVTSENRPVCLAGPVHLNNSWNLGSRTTNRMMKLQPIKAAPEGGISDVVEKSIKEAQETCAGDPVSGECVAAWDEVEELSAAASHARDKKKADGSDPLEEYCKDNPETNECRTYDN
>DisProt|DP00555|uniprot|Q16143|unigene|Hs.90297|sp|Q16143	134	MDVFMKGLSMAKEGVVAAAEKTKQGVTEAAEKTKEGVLYVGSKTREGVVQGVASVAEKTKEQASHLGGAVFSGAGNIAAATGLVKREEFPTDLKPEEVAQEAAEEPLIEPLMEPEGESYEDPPQEEYQEYEPEA
>DisProt|DP00556|unigene|117177|sp|P12003	135	SIARRANRILLVAKREVENSEDPKFREAVKAASDELSKTISPMVMDAKAVAGNISDPGLQKSFLDSGYRILGAVAKVREAFQPQEPDFPPPPPDLEHLHLTDELAPPKPPLPEGEVPPPRPPPPEEKDEEFPEQK
>DisProt|DP00120|uniprot|P12957|unigene|Gga.4988|sp|CALD1_CHICK	136	RLEQYTSAVVGNKAAKPAKPAASDLPVPAEGVRNIKSMWEKGNVFSSPGGTGTPNKETAGLKVGVSSRINEWLTKTPEGNKSPAPKPSDLRPGDVSGKRNLWEKQSVEKPAASSSKVTATGKKSETNGLRQFEKEP
>DisProt|DP00070|uniprot|P37840-1|unigene|Hs.21374|sp|SYUA_HUMAN	140	MDVFMKGLSKAKEGVVAAAEKTKQGVAEAAGKTKEGVLYVGSKTKEGVVHGVATVAEKTKEQVTNVGGAVVTGVTAVAQKTVEGAGSIAAATGFVKKDQLGKNEEGAPQEGILEDMPVDPDNEAYEMPSEEGYQDYEPEA
>DisProt|DP00196|uniprot|P20810|unigene|Hs.436186|sp|ICAL_HUMAN	141	AVPVESKPDKPSGKSGMDAALDDLIDTLGGPEETEEENTTYTGPEVSDPMSSTYIEELGKREVTIPPKYRELLAKKEGITGPPADSSKPIGPDDAIDALSSDFTCGSPTAAGKKTEKEESTEVLKAQSAGTVRSAAPPQEK
>DisProt|DP00134|uniprot|Q06787|unigene|Hs.103183|sp|FMR1_HUMAN	142	FAEDVIQVPRNLVGKVIGKNGKLIQEIVDKSGVVRVRIEAENEKNVPQEEEIMPPNSLPSNNSRVGPNAPEEKKHLDIKENSTHFSQPNSTKVQRVLVASSVVAGESQKPELKAWQGMVPFVFVGTKDSIANATVLLDYHLN
>DisProt|DP00186|uniprot|Q95V77|sp|LEA1_APHAV	143	MSSQQNQNRQGEQQEQGYMEAAKEKVVNAWESTKETLSSTAQAAAEKTAEFRDSAGETIRDLTGQAQEKGQEFKERAGEKAEETKQRAGEKMDETKQRAGEMRENAGQKMEEYKQQGKGKAEELRDTAAEKLHQAGEKVKGRD
>DisProt|DP00161|uniprot|P77173|sp|ZIPA_ECOLI	147	RPLKRMKSKRDDDSYDEDVEDDEGVGEVRVHRVNHAPANAQEHEAARPSPQHQYQPPYASAQPRQPVQQPPEAQVPPQHAPHPAQPVQQPAYQPQPEQPLQQPVSPQVAPAPQPVHSAPQPAQQAFQPAEPVAAPQPEPVAEPAPVM
>DisProt|DP00092|uniprot|Q08209|unigene|Hs.435512|sp|PP2BA_HUMAN	148	DDELGSEEDGFDGATAAARKEVIRNKIRAIGKMARVFSVLREESESVLTLKGLTPTGMLPSGVLSGGKQTLQSATVEAIEADEAIKGFSPQHKITSFEEAKGLDRINERMPPRRDAMPSDANLNSINKALTSETNGTDSNGSNSSNIQ
>DisProt|DP00159|uniprot|P09803|unigene|Mm.35605|sp|CADH1_MOUSE	149	RTVVKEPLLPPDDDTRDNVYYYDEEGGGEEDQDFDLSQLHRGLDARPEVTRNDVAPTLMSVPQYRPRPANPDEIGNFIDENLKAADSDPTAPPYDSLLVFDYEGSGSEAASLSSLNSSESDQDQDYDYLNEWGNRFKKLADMYGGGEDD
>DisProt|DP00174|uniprot|P16949|unigene|Hs.209983|sp|STMN1_HUMAN	149	MASSDIQVKELEKRASGQAFELILSPRSKESVPEFPLSPPKKKDLSLEEIQKKLEAAEERRKSHEAEVLKQLAEKREHEKEVLQKAIEENNNFSKMAEEKLTHKMEANKENREAQMAAKLERLREKDKHIEEVRKNKESKDPADETEAD
>DisProt|DP00354|uniprot|O50835|sp|O50835_BORBU	150	KKGKGKIARKKGKSKVSRKEPYIHSLKRDSANKSNFLQKNVILEEESLKTELLKEQSETRKEKIQKQQDEYKGMTQGSLNSLSGESGELEEPIESNEIDLTIDSDLRPKSSLQGIAGSNSISYTDEIEEEDYDQYYLDEYDEEDEEEIRL
>DisProt|DP00303|uniprot|P02185|sp|MYG_PHYCA	153	VLSEGEWQLVLHVWAKVEADVAGHGQDILIRLFKSHPETLEKFDRFKHLKTEAEMKASEDLKKHGVTVLTALGAILKKKGHHEAELKPLAQSHATKHKIPIKYLEFISEAIIHVLHSRHPGDFGADAQGAMNKALELFRKDIAAKYKELGYQG
>DisProt|DP00094|uniprot|P04177|unigene|Rn.11082|sp|TY3H_RAT	155	MPTPSAPSPQPKGFRRAVSEQDAKQAEAVTSPRFIGRRQSLIEDARKEREAAAAAAAAAVASSEPGNPLEAVVFEERDGNAVLNLLFSLRGTKPSSLSRAVKVFETFEAKIHHLETRPAQRPLAGSPHLEYFVRFEVPSGDLAALLSSVRRVSDD
>DisProt|DP00112|uniprot|P22239|sp|DHB_CRAPL	155	MAQFGGEKYGGRHTDEYGNPIQQGAGAHRGGGIMGGGQQAGQHGTTGVLGHGTAGQHGTTGGGLGHGTAGTGGALGGQHRRSGSSSSSSSSESDGEGGRRKKGMKDKMKEKLPGGHGTTTDQQQYGTAATHGQAQQHEKKGIMDKIKEKLPGGQH
>DisProt|DP00269|uniprot|Q24298|unigene|Dm.4731|sp|CADE_DROME	158	QKKQKNGWHEKDIDDIRETIINYEDEGGGERDTDYDLNVLRTQPFYEEKLYKDPHALQGNMRDPNDIPDIADFLGDKKENCDRDVGATTVDDVRHYAYEGDGNSDGSLSSLASCTDDGDLNFDYLSNFGPRFRKLADMYGEEPSDTDSNVDDDQGWRI
>DisProt|DP00127|uniprot|Q06556|sp|Q06556_STRDY	160	EGPTEGENNLGGQSEEITITEDSQSGMSGQNPGSGNETVVEDTQTSQEDIVLGGPGQVIDFTEDSQPGMSGNNSHTITEDSKPSQEDEVIIGGQGQVIDFTEDTQSGMSGDNSHTDGTVLEEDSKPSQEDEVIIGGQGQVIDFTEDTQTGMSGAGQVESP
>DisProt|DP00016|uniprot|P38936|unigene|Hs.370771|sp|CDN1A_HUMAN	164	MSEPAGDVRQNPCGSKACRRLFGPVDSEQLSRDCDALMAGCIQEARERWNFDFVTETPLEGDFAWERVRGLGLPKLYLPTGPRRGRDELGGGRRPGTSPALLQGTAEEDHVDLSLSCTLVPRSGEQAEGSPGGPGDSQGRKRRQTSMTDFYHSKRRLIFSKRKP
>DisProt|DP00078|uniprot|P01100|unigene|Hs.707896|sp|FOS_HUMAN	165	MSVASLDLTGGLPEVATPESEEAFTLPLLNDPEPKPSVEPVKSISSMELKTEPFDDFLFPASSRPSGSETARSVPDMDLSGSFYAADWEPLHSGSLGMGPMATELEPLCTPVVTCTPSCTAYTSSFVFTYPEADSFPSCAAAHRKGSSSNEPSSDSLSSPTLLAL
>DisProt|DP00071|uniprot|P23441|unigene|Rn.34265|sp|NKX21_RAT	166	MSMSPKHTTPFSVSDILSPLEESYKKVGMEGGGLGAPLAAYRQGQAAPPAAAMQQHAVGHHGAVTAAYHMTAAGVPQLSHSAVGGYCNGNLGNMSELPPYQDTMRNSASGPGWYGANPDPRFPAISRFMGPASGMNMSGMGGLGSLGDVSKNMAPLPSAPRRKRRV
>DisProt|DP00325|uniprot|P01099|sp|PPR1A_RABIT	166	MEQDNSPRKIQFTVPLLEPHLDPEAAEQIRRRRPTPATLVLTSDQSSPEVDEDRIPNPLLKPSLAMSPRQRKKMTRTTPTMKELQMMVEHHLGQQEQGEEPEGAAEGTGAQESQPPGTPGTGAESRLGPSATAQKPAQPSPRAQERRGEEPSTAKTSQDSQGASAV
>DisProt|DP00539|uniprot|P51608|unigene|Hs.200716|sp|P51608|trembl|P51608	166	VGDTSLDPNDFDFTVTGRGSPSRREQKPPKKPKSPKAPGTGRGRGRPKGSGTTRPKAATSEGVQVKRVLEKSPGKLLVKMPFQTSPGGKAEGGGATTSTQVMVIKRPGRKRKAEADPQAIPKKRGRKPGSVVAAAAAEAKKKAVKESSIRSVQETVLPIKKRKTRE
>DisProt|DP00135|uniprot|P10961|sp|HSF_YEAST	167	MNNAANTGTTNESNVSDAPRIEPLPSLNDDDIEKILQPNDIFTTDRTDASTTSSTAIEDIINPSLDPQSAASPVPSSSFFHDSRKPSTSTHLVRRGTPLGIYQTNLYGHNSRENTNPNSTLLSSKLLAHPPVPYGQNPDLLQHAVYRAQPSSGTTNAQPRQTTRRYQ
>DisProt|DP00260|uniprot|P01106-1|unigene|Hs.202453|sp|MYC_HUMAN	167	MPLNVSFTNRNYDLDYDSVQPYFYCDEEENFYQQQQQSELQPPAPSEDIWKKFELLPTPPLSPSRRSGLCSPSYVAVTPFSLRGDNDGGGGSFSTADQLEMVTELLGGDMVNQSFICDPDDETFIKNIIIQDCMWSGFSAAAKLVSEKLASYQAARKDSGSPNPARG
>DisProt|DP00530|uniprot|P12950|unigene|Zm.72794|sp|P12950|trembl|P12950	167	MEYGQQGQHGHGATGHVDQYGNPVGGVEHGTGGMRHGTGTGGMGQLGEHGGAGMGGGQFQPAREEHKTGGILHRSGSSSSSSSEDDGMGGRRKKGIKEKIKEKLPGGHKDDQHATATTGGAYGQQGHTGSAYGQQGHTGGAYATGTEGTGEKKGIMDKIKEKLPGQH
>DisProt|DP00047|uniprot|P02687|unigene|Bt.64741|sp|MBP_BOVIN	169	AAQKRPSQRSKYLASASTMDHARHGFLPRHRDTGILDSLGRFFGSDRGAPKRGSGKDGHHAARTTHYGSLPQKAQGHRPQDENPVVHFFKNIVTPRTPPPSQGKGRGLSLSRFSWGAEGQKPGFGYGGRASDYKSAHKGLKGHDAQGTLSKIFKLGGRDSRSGSPMARR
>DisProt|DP00237|uniprot|P04370|unigene|Mm.252063|sp|MBP_MOUSE	169	MASQKRPSQRSKYLATASTMDHARHGFLPRHRDTGILDSIGRFFSGDRGAPKRGSGKDSHTRTTHYGSLPQKSQHGRTQDENPVVHFFKNIVTPRTPPPSQGKGRGLSLSRFSWGAEGQKPGFGYGGRASDYKSAHKGFKGAYDAQGTLSKIFKLGGRDSRSGSPMARR
>DisProt|DP00381|uniprot|P35869|unigene|Hs.171189|sp|AHR_HUMAN	169	NLGIDFEDIRHMQNEKFFRNDFSGEVDFRDIDLTDEILTYVQDSLSKSPFIPSDYQQQQSLALNSSCMVQEHLHLEQQQQHHQKQVVVEPQQQLCQKMKHMQVNGMFENWNSNQFVPFNCPQQDPQQYNVFTDLHGISQEFPYKSEMDSMPYTQNFISCNQPVLPQHSK
>DisProt|DP00564|sp|Q6P8Z1	170	MPLHVSLANGNRDLDYDSVQPYFMCDDEEEDVHHQQPPQPPAPSEDIWKKFELLPTPRPSPGHAGLYSPPCEAVAVSFAPRDHDGDSFSIADLPELPGGDAVKQSFVCDPDDETFVKNIILQDCMWNGFSASAKLVSKLDPYQAVRKEGTGVSLAADVEPATPPDCTCNT
>DisProt|DP00236|uniprot|P02686|unigene|Hs.551713|sp|MBP_HUMAN	171	MASQKRPSQRHGSKYLATASTMDHARHGFLPRHRDTGILDSIGRFFGGDRGAPKRGSGKDSHHPARTAHYGSLPQKSHGRTQDENPVVHFFKNIVTPRTPPPSQGKGRGLSLSRFSWGAEGQRPGFGYGGRASDYKSAHKGFKGVDAQGTLSKIFKLGGRDSRSGSPMARR
>DisProt|DP00144|uniprot|Q9VAN6|unigene|Dm.4801|sp|SLBP_DROME	175	MLCEDQHMSVENTPQKGSGSLNSSASSISIDVKPTMQSWAQEVRAEFGHSDEASSSLNSSAASCGSLAKKETADGNLESKDGEGREMAFEFLDGVNEVKFERLVKEEKLKTPYKRRHSFTPPSNENSRSNSPNSSNSSANGDAAAPKGGNNPHSRNSKKSGNFRAHKEEKRVRHN
>DisProt|DP00546|uniprot|Q9NX55|unigene|Hs.631692|sp|Q9NX55	175	MTGFLLPPASRASRTGAERRCGPGPAPPPPRAEAVSVAGSRREVGLMRRRGEIDMATEGDVELELETETSGPERPPEKPRKHDSGAADLERVTDYAEEKEIQSSNLETAMSVIGDRRSREQKAKQEREKELAKVTIKKEDLELIMTEMEISRAAAERSLREHMGNVVEALIALTN
>DisProt|DP00474|uniprot|Q43125|sp|Q43125|pir|S39058	176	SEEGLGDSAEVEEAPIEFPRDITMEETEPTRLNPNRRYEDQMVPSITSSLIRPEEDEESSLNLRNSVGDSRAEVPRNMVNTNQAQQRRAEPASNQVTAMIPEFNIRIVAESTEDSTAESSSSGRRERSGGIVPEWSPGYSEQFPSEENRIGGGSTTSSYLQNHHEILNWRRLSQTG

>DisProt|DP00193|uniprot|P02754|unigene|Bt.385|sp|LACB_BOVIN	178	MKCLLLALALTCGAQALIVTQTMKGLDIQKVAGTWYSLAMAASDISLLDAQSAPLRVYVEELKPTPEGDLEILLQKWENGECAQKKIIAEKTKIPAVFKIDALNENKVLVLDTDYKKYLLFCMENSAEPEQSLACQCLVRTPEVDDEALEKFDKALKALPMHIRLSFNPTQLEEQCHI
>DisProt|DP00192|uniprot|P02668|unigene|Bt.49421|sp|CASK_BOVIN	182	MMKSFFLVVTILALTLPFLGAQEQNQEQPIRCEKDERFFSDKIAKYIPIQYVLSRYPSYGLNYYQQKPVALINNQFLPYPYYAKPAAVRSPAQILQWQVLSNTVPAKSCQAQPTTMARHPHPHLSFMAIPPKKNQDKTEIPTINTIASGEPTSTPTTEAVESTVATLEDSPEVIESPPEINT
>DisProt|DP00074|uniprot|P03372-1|unigene|Hs.208124|sp|ESR1_HUMAN	184	MTMTLHTKASGMALLHQIQGNELEPLNRPQLKIPLERPLGEVYLDSSKPAVYNYPEGAAYEFNAAAAANAQVYGQTGLPYGPGSEAAAFGSNGLGGFPPLNSVSPSPLMLLHPPPQLSPFLQPHGQQVPYYLENEPSGYTVREAGPPAFYRPNSDNRRQGGRERLASTNDKGSMAMESAKETRY
>DisProt|DP00542	185	GKNKSKRIQALKFRHARDKRAGFEIDNNDDTIEEFFGSAYRKKGKGKGTTVGMGKSSRRFINMYGFDPTEYSFIQFVDPLTGAQIEENVYADIRDIQERFSEVRKKMVEDDEIEMQALGSNTSIHAYFRKDWSDKALKIDLMPHNPLKICDKTNGIAKFPEREFELRQTGPAVEVDVKDIPKQEV
>DisProt|DP00291|uniprot|Q9QXT8-1|unigene|Mm.315292|sp|CSEN_MOUSE	192	SELELSTVRHQPEGLDQLQAQTKFTKKELQSLYRGFKNECPTGLVDEDTFKLIYSQFFPQGDATTYAHFLFNAFDADGNGAIHFEDFVVGLSILLRGTVHEKLKWAFNLYDINKDGCITKEEMLAIMKSIYDMMGRHTYPILREDAPLEHVERFFQKMDRNQDGVVTIDEFLETCQKDENIMNSMQLFENVI
>DisProt|DP00560|sp|Q02597	193	AKGKKQRQKLKFRNARDNKMGREVYGDDDTIEHFFGDAYTKKGKSKGRTRGIGHKNRKFINMYGFDPEDFSAVRFVDPLTGATLDDNPFTDITLVQKHFGDIRMDLLGEDELDSNEIRMNKTIQAYYMNNKTGKALKVDLTPHIPLKVCDLHATIAGFPERENELRQTGKAQPINIDEVPRANNELVPVDHES
>DisProt|DP00036|uniprot|P22121|sp|HSF_KLULA	195	MGHNDSVETMDEISNPNNILLPHDGTGLDATGISGSQEPYGMVDVLNPDSLKDDSNVDEPLIEDIVNPSLDPEGVVSAEPSNEVGTPLLQQPISLDHVITRPASAGGVYSIGNSSTSSAAKLSDGDLTNATDPLLNNAHGHGQPSSESQSHSNGYHKQGQSQQPLLSLNKRKLLAKAHVDKHHSKKKLSTTRARP
>DisProt|DP00275|uniprot|Q7CJN0|sp|Q7CJN0_YERPE	195	YANEPKVCFYMDDDYNGESLCAAQGNSVASIPDKWNDRISSISIPHGLVVTVYEDVDFLGASRSFEADVDLVSDKDLIYLNDNISAFKIKKAVCFYGESNFTGDSLCLSSGEQFDLYRGNYPERKQSHLVNPLNDEVYSIKIPPGMQTTVYEDDDYNGKYFVLTEDYTPDDLLIIRMNNKISSMRVSQDEDFICD
>DisProt|DP00231|uniprot|P14859|unigene|Hs.493649|sp|PO2F1_HUMAN	200	MNNPSETSKPSMESGDGNTGTQTNGLDFQKQPVPVGGAISTAQAQAFLGHLHQVQLAGTSLQAAAQSLNVQSKSNEESGDSQQPSQPSQQPSVQAAIPQTQLMLAGGQITGLTLTPAQQQLLLQQAQAQAQLLAAAVQQHSASQQHSAAGATISASAATPMTQIPLSQPIQIAQDLQQLQQLQQQNLNLQQFVLVHPTTN
>DisProt|DP00524|uniprot|Q8K3G7|sp|Q8K3G7|trembl|Q8K3G7	200	MEDLDQSPLVSSSDSPPRPQPAFKYQFVREPEDEEEEEEEEEEDEDEDLEELEVLERKPAAGLSAAPVPTAPAAGAPLMDFGNDFVPPAPRGPLPAAPPVAPERQPSWDPSPVSSTVPAPSPLSAAAVSPSKLPEDDEPPARPPPPPPASVSPQAEPVWTPPAPAPAAPPSTPAAPKRRGSSGSVVVDLLYWRDIKKTGV
>DisProt|DP00421|uniprot|P07516|unigene|Bt.4706|sp|PPR1B_BOVIN	202	MDPKDRKKIQFSVPAPPSQLDPRQVEMIRRRRPTPAMLFRLSEHSSPEEEASPHQRASGEGHHLKSKRSNPCAYTPPSLKAVQRIAESHLQSISNLGENQASEEEDELGELRELGYPREEEEEEEEEDEEEEEDSQAEVLKGSRGSAGQKTTYGQGLEGPWERPPPLDGPQRDGSSEDQVEDPALNEPGEEPQRPAHPEPGT
>DisProt|DP00521|uniprot|O95997|unigene|Hs.350966|sp|O95997	202	MATLIYVDKENGEPGTRVVAKDGLKLGSGPSIKALDGRSQVSTPRFGKTFDAPPALPKATRKALGTVNRATEKSVKTKGPLKQKQPSFSAKKMTEKTVKAKSSVPASDDAYPEIEKFFPFNPLDFESFDLPEEHQIAHLPLSGVPLMILDEERELEKLFQLGPPSPVKMPSPPWESNLLQSPSSILSTLDVELPPVCCDIDI
>DisProt|DP00041|uniprot|P07746|unigene|Omy.32291|sp|HMGT_ONCMY	204	MGKDPRKPRGKMSSYAYFVQTRREEHKKKHPEASVNFSEFSKKCSERWKTMSAKEKGKFEDLAKLDKVRYEREMRSYIPPKGEKKKRFKDPNAPKRPSSAFFIFCADFRPQVKGETPGLSIGDVAKKLGEKWNNLTAEDKVPYEKKASRLKEKYEKDITAYRNKGKVPVSMPAKAAAPAKDDDDDDDDDDDDEDDDDDDDEDDE
>DisProt|DP00190|uniprot|P38038|sp|CYSJ_ECOLI	204	ATPAPAAEMPGITIISASQTGNARRVAEALRDDLLAAKLNVKLVNAGDYKFKQIASEKLLIVVTSTQGEGEPPEEAVALHKFLFSKKAPKLENTAFAVFSLGDTSYEFFCQSGKDFDSKLAELGGERLLDRVDADVEYQAAASEWRARVVDALKSRAPVAAPSQSVATGAVNEIHTSPYSKDAPLVASLSVNQKITGRNSEKDV
>DisProt|DP00563|uniprot|Q61337|unigene|Mm.4387|sp|BAD_MOUSE	204	MGTPKQPSLAPAHALGLRKSDPGIRSLGSDAGGRRWRPAAQSMFQIPEFEPSEQEDASATDRGLGPSLTEDQPGPYLAPGLLGSNIHQQGRAATNSHHGGAGAMETRSRHSSYPAGTEEDEGMEEELSPFRGRSRSAPPNLWAAQRYGRELRRMSDEFEGSFKGLPRPKSAGTATQMRQSAGWTRIIQSWWDRNLGKGGSTPSQ
>DisProt|DP00232|uniprot|P11845|unigene|Ocu.1811|sp|IPP2_RABIT	205	MAASTASHRPIKGILKNKTSSTSSRVASAEQPRGSVDEELSKKSQKWDEMNILATYHPADKDYGLMKIDEPSTPYHSMIGDDDDAYSDTETTEAMTPDTLAKKLAAAEGSEPKYRIREQESSGEEDSDLSPEEREKKRQFEMKRKLHYNEGLNIKLARQLISKDLHDDEEDEEMSETADGESMNTEESNQGSTPSDQRQNKSQSS
>DisProt|DP00068|uniprot|P60881|unigene|Rn.107689|sp|SNP25_RAT	206	MAEDADMRNELEEMQRRADQLADESLESTRRMLQLVEESKDAGIRTLVMLDEQGEQLDRVEEGMNHINQDMKEAEKNLKDLGKCCGLYICPCNKLKSSDAYKKAWGNNQDQVVASQPARVVDEREQMAISGGFIRRVTNDARENEMDEDLEQVSGIIGNLRHMALDMGNEIDTQNRQIDRIMEKADSNKTRIDEANQRATKMLGSG
>DisProt|DP00367|uniprot|Q9RP19|sp|UREG_BACPA	211	MKTIHLGIGGPVGSGKTTLVKTLSEALKEEYSIAVITNDIYTREDANFLINENILEKDRIIGVETGGCPHTAIREDASMNFEAIEELKNRFDDLEIILLESGGDNLSATFSPELVDAFIYVIDVSEGGDIPRKGGPGVTRSDFLMVNKTELAPYVGVDLDTMKNDTIKARNGRPFTFANIKTKKGLDEIIAWIKSDLLLEGKTNESASESK
>DisProt|DP00287|uniprot|P40337-1|unigene|Hs.517792|sp|VHL_HUMAN	213	MPRRAENWDEAEVGAEEAGVEEYGPEEDGGEESGAEESGPEESGPEELGAEEEMEAGRPRPVLRSVNSREPSQVIFCNRSPRVVLPVWLNFDGEPQPYPTLPPGTGRRIHSYRGHLWLFRDAGTHDGLLVNQTELFVPSLNVDGQPIFANITLPVYTLKERCLQVVRSLVKPENYRRLDIVRSLYEDLEDHPNVQKDLERLTQERIAHQRMGD
>DisProt|DP00075|uniprot|P11387|unigene|Hs.472737|sp|TOP1_HUMAN	214	MSGDHLHNDSQIEADFRLNDSHKHKDKHKDREHRHKEHKKEKDREKSKHSNSEHKDSEKKHKEKEKTKHKDGSSEKHKDKHKDRDKEKRKEEKVRASGDAKIKKEKENGFSSPPQIKDEPEDDGYFVPPKEDIKPLKRPRDEDDADYKPKKIKTEDTKKEKKRKLEEEEDGKLKKPKNKDKDKKVPEPDNKKKKPKKEEEQKWKWWEEERYPEG
>DisProt|DP00330|uniprot|P02662|unigene|Bt.59369|sp|CASA1_BOVIN	214	MKLLILTCLVAVALARPKHPIKHQGLPQEVLNENLLRFFVAPFPEVFGKEKVNELSKDIGSESTEDQAMEDIKQMEAESISSSEEIVPNSVEQKHIQKEDVPSERYLGYLEQLLRLKKYKVPQLEIVPNSAEERLHSMKEGIHAQQKEPMIGVNQELAYFYPELFRQFYQLDAYPSGAWYYVPLGTQYTDAPSFSDIPNPIGSENSEKTTMPLW
>DisProt|DP00136|uniprot|P15865|unigene|Rn.196361|sp|H12_RAT	217	MSETAPAAPAAPAPAEKTPIKKKARKAAGGAKRKASGPPVSELITKAVAASKERSGVSLAALKKALAAAGYDVEKNNSRIKLGLKSLVSKGTLVQTKGTGASGSFKLNKKAASGEAKPKAKKAGAAKAKKPAGAAKKPKKATGTATPKKSTKKTPKKAKKPAAGAKKAKSPKKAKATKAKKAPKSPAKARAVKPKAAKPKTSKPKAAKPKKTAAKKK
>DisProt|DP00520|uniprot|Q969F2|unigene|Hs.240951|trembl|Q969F2	217	MGKLQSKHAAAARKRRESPEGDSFVASAYASGRKGAEEAERRARDKQELPNGDPKEGPFREDQCPLQVALPAEKAEGREHPGQLLSADDGERAANREGPRGPGGQRLNIDALQCDVSVEEDDRQEWTFTLYDFDNCGKVTREDMSSLMHTIYEVVDASVNHSSGSSKTLRVKLTVSPEPSSKRKEGPPAGQDREPTRCRMEGELAEEPRVADRRLSA
>DisProt|DP00329|uniprot|P02666|unigene|Bt.53272|sp|CASB_BOVIN	224	MKVLILACLVALALARELEELNVPGEIVESLSSSEESITRINKKIEKFQSEEQQQTEDELQDKIHPFAQTQSLVYPFPGPIPNSLPQNIPPLTQTPVVVPPFLQPEVMGVSKVKEAMAPKHKEMPFPKYPVEPFTESQSLTLTDVENLHLPLPLLQSWMHQPHQPLPPTVMFPPQSVLSLSQSKVLPVPQKAVPYPQRDMPIQAFLLYQEPVLGPVRGPFPIIV
>DisProt|DP00170|uniprot|Q39805|unigene|Gma.33428|sp|Q39805_SOYBN	226	MASYQKHYDDQGRKVDEYGNVEKQTDEYGNPVHAASVTYVATRTAAGGYSDDINKQHDTTNAYGVDTGRQHSSGGYDGDTNKHHGTTGGYNDDTNRHHGTTGVYGIDTDRQQHGTTGGYAGDTGRQHGNIGGPYYGTNTADTGTGPRSGTTGGTGYGGTGGTDYGTTGGTGYGSGTGYGVNTGGAHTEAGYRKEHRQHDQSHGDQNEKKGIMDKIKEKLPGGHSDK
>DisProt|DP00199|uniprot|P05814|unigene|Hs.2242|sp|CASB_HUMAN	226	MKVLILACLVALALARETIESLSSSEESITEYKQKVEKVKHEDQQQGEDEHQDKIYPSFQPQPLIYPFVEPIPYGFLPQNILPLAQPAVVLPVPQPEIMEVPKAKDTVYTKGRVMPVLKSPTIPFFDPQIPKLTDLENLHLPLPLLQPLMQQVPQPIPQTLALPPQPLWSVPQPKVLPIPQQVVPYPQRAVPVQALLLNQELLLNPTHQIYPVTQPLAPVHNPISV
>DisProt|DP00198|uniprot|P33905|sp|TRAR_RHIRD	234	MQHWLDKLTDLAAIEGDECILKTGLADIADHFGFTGYAYLHIQHRHITAVTNYHRQWQSTYFDKKFEALDPVVKRARSRKHIFTWSGEHERPTLSKDERAFYDHASDFGIRSGITIPIKTANGFMSMFTMASDKPVIDLDREIDAVAAAATIGQIHARISFLRTTPTAEDAAWLDPKEATYLRWIAVGKTMEEIADVEGVKYNSVRVKLREAMKRFDVRSKAHLTALAIRRKLI
>DisProt|DP00587|uniprot|Q9CQK7|unigene|Mm.28528|sp|Q9CQK7	243	MTDYGEEQRNELEALESIYPDSFTVLSESPPSFTITVTSEAGENDETVQTTLKFTYSEKYPDETPLYEIFSQENLEDNDVSDILKLLALQAEENLGMVMIFTLVTAVQEKLNEIVDQIKTRREEEKKQKEKEAEEAEKKLFHGTPVTIENFLSWKAKFDAELLEIKKKRMKEEEQAGKNKLSGKQLFETDHNLDTSDIQFLEDAGNNVEVDESLFQEMDDLELEDGEDDPDYNPVAPGSDSSD
>DisProt|DP00076|uniprot|P06786|sp|TOP2_YEAST	250	GNVPNKGSKTKGKGKRKLVDDEDYDPSKKNKKSTARKGKKIKLEDKNFERILLEQKLVTKSKAPTKIKKEKTPSVSETKTEEEENAPSSTSSSSIFDIKKEDKDEGELSKISNKFKKISTIFDKMGSTSATSKENTPEQDDVATKKNQTTAKKTAVKPKLAKKPVRKQQKVVELSGESDLEILDSYTDREDSNKDEDDAIPQRSRRQRSSRAASVPKKSYVETLELSDDSFIEDDEEENQGSDVSFNEED
>DisProt|DP00483|sp|Q8TFB8|pir|EFBYS2	253	MSDSNQGNNQQNYQQYSQNGNQQQGNNRYQGYQAYNAQAQPAGGYYQNYQGYSGYQQGGYQQYNPDAGYQQQYNPQGGYQQYNPQGGYQQQFNPQGGRGNYKNFNYNNNLQGYQAGFQPQSQGMSLNDFQKQQKQAAPKPKKTLKLVSSSGIKLANATKKVDTKPAESDKKEEEKSAETKEPTKEPTKVEEPVKKEEKPVQTEEKTEEKSELPKVEDLKISESTHNTNNANVTSADALIKEQEEEVDDEVVND
>DisProt|DP00008|uniprot|Q64693|unigene|Mm.897|sp|OBF1_MOUSE	256	MLWQKSTAPEQAPAPPRPYQGVRVKEPVKELLRRKRGHTSVGAAGPPTAGVLPHQPLATYSTVGPSCLDMEVSASTVTEEGTLCAGWLSQPAPATLHALAPWTPYTEYVSHEAVSCPYSTDMYVQPVCPSYTVVGPSSVLTYASPPLITNVTPRSTATPAVGPQLEGPEHQAPLTYFPWPQPLSTLPTSSLQYQPPAPTLSGPQFVQLPISIPEPVLQDMDDPRRAISSLTIDKLLLEEEESNTYELNHTLSVEGF
>DisProt|DP00541|uniprot|Q91V27|unigene|Mm.105208|sp|Q91V27|trembl|Q91V27	257	GGGGSEPSLEEGNGDSEQTDEDGDLDTEARDQPLNSKKKKRLLSFRDVDFEEDSDHLVQPCSQTLGLSSVPESAHSLQSLSGEPYSEDTTSLEPEGLEETGARALGCRPSPEVQPCSPLPSGEDAHAELDSPAASCKSAFGTTAMPGTDDVRGKHLPSQYLADVDTSDEDSIQGPRAASQHSKRRARTVPETQILELNKRMSAVEHLLVHLENTVLPPSAQEPTVETHPSADTEEETLRRRLEELTSNISGSSTSSE
>DisProt|DP00163|uniprot|Q03768|sp|GIR2_YEAST	265	MDYKEEQKQELEVLESIYPDELRIINDEYPKIKFEVAIKLELDTGDSTSVLTKEHTIIAEFKLPENYPDEPCLISLEAQEVALNDNEEDNEEDEDEVEYDDHGNKVLKKFENLPDLISFKGYLPELTVQLESQIETDMLLGMQMCFALISSIKERCEQWYSEQLNKLEKQYELEAQEREKKEQAKFHGTKVTRESYLEWRSKFRQELKLDERDQVRRMKAHHGKLTGKQMFEQGVVGTGDEYMEEDDASVDDVAKGLAKTEIANQ
>DisProt|DP00351|uniprot|Q27974|unigene|Bt.65042|sp|AUXI_BOVIN	267	PSGPTSTQSTPRRSATSTSASPTLRVGEGATFDPFGAPSKPSGQDLLGSFLNTASASSDPFLQPTRSPSPTVHASSTPAVNIQPDVSGAWDWHTKPGGFGMGSKSAATSPTGSSHGTPTHQNKPQTLDPFADLGTLGGSSFASKPSTPTGLGGGFPPLSSPQKASPQPMGGGWQQGGGYNWQQTQSKPQSSMPHSSPQNRPNYNVSFSSMPGGQNERGKAAANLEGKQKAADFEDLLSGQGFNAHKDKKGPRTIAEMRKEEMAKEMD
>DisProt|DP00233|uniprot|P14448-1|unigene|Gga.34280|sp|FIBA_CHICK	273	HFKLKPSPEMQAMSAFNNIKQMQVVLERPETDHVAEARGDSSPSHTGKLITSSHRRESPSLVDKTSSASSVHRCTRTVTKKVISGPDGPREEIVEKMVSSDGSDCSHLQGGREGSTYHFSGTGDFHKLDRLLPDLESFFTHDSVSTSSRHSIGSSTSSHVTGAGSSHLGTGGKDKFTDLGEEEEDDFGGLQPSGFAAGSASHSKTVLTSSSSSFNKGGSTFETKSLKTRETSEQLGGVQHDQSAEDTPDFKARSFRPAAMSTRRSYNGKDCDD

>DisProt|DP00227|uniprot|Q9FUW7|sp|Q9FUW7_WHEAT	280	MKTFLIFVLLAMAMKIATAARELNPSNKELQSPQQSFSYQQQPFPQQPYPQQPYPSQQPYPSQQPFPTPQQQFPEQSQQPFTQPQQPTPIQPQQPFPQQPQQPQQPFPQPQQPFPWQP<br>QQPFPQTQQSFPLQPQQPFPQQPQQPFPQPQLPFPQQSEQIIPQQLQQPFPLQPQQPFPQQPQQPFPQPQQPIPVQPQQSFPQQSQQSQQPFAQPQQLFPELQQPIPQQPQQPFPLQP<br>QQPFPQQPQQPFPQQPQQSFPQQPQQPYPQQQPYGSSLTSIGGQ
>DisProt|DP00052|uniprot|P07214|unigene|Mm.291442|sp|SPRC_MOUSE	285	APQQTEVAEEIVEEETVVEETGVPVGANPVQVEMGEFEDGAEETVEEVVADNPCQNHHCKHGKVCELDESNTPMCVCQDPTSCPAPIGEFEKVCSNDNKTFDSSCHFFATKCTLEGTK<br>KGHKLHLDYIGPCKYIAPCLDSELTEFPLRMRDWLKNVLVTLYERDEGNNLLTEKQKLRVKKIHENEKRLEAGDHPVELLARDFEKNYNMYIFPVHWQFGQLDQHPIDGYLSHTELAP<br>LRAPLIPMEHCTTRFFETCDLDNDKYIALEEWAGCFGIKEQDINKDLVI
>DisProt|DP00467|uniprot|Q01484|sp|Q01484|pir|S37431	285	LDHSEGFSVLQEELCTAQHKQKEEQAVSKESETCDHPPIVSEEDISVGYSTFQDGVPKTEGDSSSTALFPQTHKEQVQQDFSGKMQDLPEESSLEYQQEYFVTTPGTETSETQKAMIV<br>PSSPSKTPEEVSTPAEEEKLYLQTPTSSERGGSPIIQEPEEPSEHREESSPRKTSLVIVESADNQPETCERLDEDAAFEKGDDMPEIPPETVTEEEYIDEHGHTVVKKVTRKIIRRYV<br>SSEGTEKEEIMVQGMPQEPVNIEEGDGYSKVIKRVVLKSDTEQSEDNNE
>DisProt|DP00129|uniprot|Q04207|unigene|Mm.249966|sp|TF65_MOUSE	286	PYVEIIEQPKQRGMRFRYKCEGRSAGSIPGERSTDTTKTHPTIKINGYTGPGTVRISLVTKDPPHRPHPHELVGKDCRDGYYEADLCPDRSIHSFQNLGIQCVKKRDLEQAISQRIQT<br>NNNPFHVPIEEQRGDYDLNAVRLCFQVTVRDPAGRPLLLTPVLSHPIFDNRAPNTAELKICRVNRNSGSCLGGDEIFLLCDKVQKEDIEVYFTGPGWEARGSFSQADVHRQVAIVFRT<br>PPYADPSLQAPVRVSMQLRRPSDRELSEPMEFQYLPDTDDRHRIEEKRKR
>DisProt|DP00360|uniprot|O14576-1|unigene|Hs.440364|sp|DC1I1_HUMAN	289	MSDKSDLKAELERKKQRLAQIREEKKRKEEERKKKEADMQQKKEPVQDDSDLDRKRRETEALLQSIGISPEPPLVQPLHFLTWDTCYFHYLVPTPMSPSSKSVSTPSEAGSQDSGDLG<br>PLTRTLQWDTDPSVLQLQSDSELGRRLHKLGVSKVTQVDFLPREVVSYSKETQTPLATHQSEEDEEDEEMVESKVGQDSELENQDKKQEVKEAPPRELTEEEKQQILHSEEFLIFFDR<br>TIRVIERALAEDSDIFFDYSGRELEEKDGDVQAGANLSFNRQFYDEHWSKHRV
>DisProt|DP00119|uniprot|P10163|unigene|Hs.528651|sp|PRB4_HUMAN	294	ESSSEDVSQEESLFLISGKPEGRRPQGGNQPQRPPPPPGKPQGPPPQGGNQSQGPPPPPGKPEGRPPQGGNQSQGPPPHPGKPERPPPQGGNQSQGPPPHPGKPESRPPQGGHQSQGP<br>PPTPGKPEGPPPQGGNQSQGTPPPPGKPEGRPPQGGNQSQGPPPHPGKPERPPPQGGNQSHRPPPPPGKPERPPPQGGNQSQGPPPHPGKPEGPPPQEGNKSRSARSPPGKPQGPPQQ<br>EGNKPQGPPPPGKPQGPPPPGGNPQQPQAPPAGKPQGPPPPPQGGRPPRPAQGQQPPQ
>DisProt|DP00285|uniprot|P10388|sp|GLT5_WHEAT	294	YYPTSPQQPGQWQQPEQGQPGYYPTSPQQPGQLQQPAQGQQPGQGQQGQQPGQGQPGYYPTSSQLQPGQLQQPAQGQQGQQPGQAQQGQQPGQGQQPGQGQQGQQPGQGQQPGQGQQG<br>QQLGQGQQGYYPTSLQQSGQGQPGYYPTSLQQLGQGQSGYYPTSPQQPGQGQQPGQLQQPAQGQQPGQGQQGQQPGQGQQGQQPGQGQQPGQGQPGYYPTSPQQSGQGQPGYYPTSSQ<br>QPTQSQQPGQGQQGQQVGQGQQAQQPGQGQQPGQGQPGYYPTSPQQSGQGQPGYYLTS
>DisProt|DP00262|uniprot|Q16665-1|unigene|Hs.597216|sp|HIF1A_HUMAN	296	AAGDTIISLDFGSNDTETDDQQLEEVPLYNDVMLPSPNEKLQNINLAMSPLPTAETPKPLRSSADPALNQEVALKLEPNPESLELSFTMPQIQDQTPSPSDGSTRQSSPEPNSPSEYC<br>FYVDSDMVNEFKLELVEKLFAEDTEAKNPFSTQDTDLDLEMLAPYIPMDDDFQLRSFDQLSPLESSSASPESASPQSTVTVFQQTQIQEPTANATTTTATTDELKTVTKDRMEDIKIL<br>IASPSPTHIHKETTSATSSPYRDTQSRTASPNRAGKGVIEQTEKSHPRSPNVLSVALSQR
>DisProt|DP00218|uniprot|Q90623-2|unigene|Gga.3159|sp|MYPT1_CHICK	299	MKMADAKQKRNEQLKRWIGSETDLEPPVVKRKKTKVKFDDGAVFLAACSSGDTEEVLRLLERGADINYANVDGLTALHQACIDDNVDMVKFLVENGANINQPDNEGWIPLHAAASCGY<br>LDIAEYLISQGAHVGAVNSEGDTPLDIAEEEAMEELLQNEVNRQGVDIEAARKEEERIMLRDARQWLNSGHINDVRHAKSGGTALHVAAAKGYTEVLKLLIQARYDVNIKDYDGWTPL<br>HAAAHWGKEEACRILVENLCDMEAVNKVGQTAFDVADEDILGYLEELQKKQNLLHSEKREKKS
>DisProt|DP00240|uniprot|P35611-1|unigene|Hs.183706|sp|ADDA_HUMAN	308	CSPLRHSFQKQQREKTRWLNSGRGDEASEEGQNGSSPKSKTKWTKEDGHRTSTSAVPNLFVPLNTNPKEVQEMRNKIREQNLQDIKTAGPQSQVLCGVVMDRSLVQGELVTASKAIIE<br>KEYQPHVIVSTTGPNPFTTLTDRELEEYRREVERKQKGSEENLDEAREQKEKSPPDQPAVPHPPPSTPIKLEEDLVPEPTTGDDSDAATFKPTLPDLSPDEPSEALGFPMLEKEEEAH<br>RPPSPTEAPTEASPEPAPDPAPVAEEAAPSAVEEGAAADPGSDGSPGKSPSKKKKKFRTPSFLKKSKKKSDS
>DisProt|DP00253|uniprot|P26645|unigene|Mm.477725|sp|MARCS_MOUSE	309	MGAQFSKTAAKGEATAERPGEAAVASSPSKANGQENGHVKVNGDASPAAAEPGAKEELQANGSAPAADKEEPASGSAATPAAAEKDEAAAATEPGAGAADKEAAEAEPAEPSSPAAEA<br>EGASASSTSSPKAEDGAAPSPSSETPKKKKKRFSFKKSFKLSGFSFKKSKKESGEGAEAEGATAEGAKDEAAAAAGGEGAAAPGEQAGGAGAEGAAGGEPREAEAAEPEQPEQPEQPA<br>AEEPQAEEQSEAAGEKAEEPAPGATAGDASSAAGPEQEAPAATDEAAASAAPAASPEPQPECSPEAPPAPTAE
>DisProt|DP00214|uniprot|P10451|unigene|Hs.313|sp|OSTP_HUMAN	314	MRIAVICFCLLGITCAIPVKQADSGSSEEKQLYNKYPDAVATWLNPDPSQKQNLLAPQNAVSSEETNDFKQETLPSKSNESHDHMDDMDDEDDDDHVDSQDSIDSNDSDDVDDTDDSH<br>QSDESHHSDESDELVTDFPTDLPATEVFTPVVPTVDTYDGRGDSVVYGLRSKSKKFRRPDIQYPDATDEDITSHMESEELNGAYKAIPVAQDLNAPSDWDSRGKDSYETSQLDDQSAE<br>THSHKQSRLYKRKANDESNEHSDVIDSQELSKVSREFHSHEFHSHEDMLVVDPKSKEEDKHLKFRISHELDSASSEVN
>DisProt|DP00017|uniprot|P49918|unigene|Hs.106070|sp|CDN1C_HUMAN	316	MSDASLRSTSTMERLVARGTFPVLVRTSACRSLFGPVDHEELSRELQARLAELNAEDQNRWDYDFQQDMPLRGPGRLQWTEVDSDSVPAFYRETVQVGRCRLLLAPRPVAVAVAVSPP<br>LEPAAESLDGLEEAPEQLPSVPVPAPASTPPPVPVLAPAPAPAPAPVAAPVAAPVAVAVLAPAPAPAPAPAPAPAPVAAPAPAPAPAPAPAPAPAPAPDAAPQESAEQGANQGQRGQE<br>PLADQLHSGISGRPAAGTAAASANGAAIKKLSGPLISDFFAKRKRSAPEKSSGDVPAPCPSPSAAPGVGSVEQTPRKRLR
>DisProt|DP00332|uniprot|P21815|unigene|Hs.518726|sp|SIAL_HUMAN	317	MKTALILLSILGMACAFSMKNLHRRVKIEDSEENGVFKYRPRYYLYKHAYFYPHLKRFPVQGSSDSSEENGDDSSEEEEEEEETSNEGENNEESNEDEDSEAENTTLSATTLGYGEDA<br>TPGTGYTGLAAIQLPKKAGDITNKATKEKESDEEEEEEEEGNENEESEAEVDENEQGINGTSTNSTEAENGNGSSGVDNGEEGEEESVTGANAEGTTETGGQGKGTSKTTTSPNGGFE<br>PTTPPQVYRTTSPPFGKTTTVEYEGEYEYTGANDYDNGYEIYESENGEPRGDNYRAYEDEYSYFKGQGYDGYDGQNYYHHQ
>DisProt|DP00241|uniprot|P35612-1|unigene|Hs.188528|sp|ADDB_HUMAN	318	FEEDGAPVPALRQHAQKQQKEKTRWLNTPNTYLRVNVADEVQRSMGSPRPKTTWMKADEVEKSSSGMPIRIENPNQFVPLYTDPQEVLEMRNKIREQNRQDVKSAGPQSQLLASVIAE<br>KSRSPSTESQLMSKGDEDTKDDSEETVPNPFSQLTDQELEEYKKEVERKKLELDGEKETAPEEPGSPAKSAPASPVQSPAKEAETKSPLVSPSKSLEEGTKKTETSKAATTEPETTQP<br>EGVVVNGREEEQTAEEILSKGLSQMTTSADTDVDTSKDKTESVTSGPMSPEGSPSKSPSKKKKKFRTPSFLKKSKKKEKVES
>DisProt|DP00472|uniprot|Q96FN7|unigene|Hs.567327|trembl|Q96FN7	324	MAMRELVEAECGGANPLMKLAGHFTQDKALRQEGLRPGPWPPGAPASEAASKPLGVASEDELVAEFLQDQNAPLVSRAPQTFKMDDLLAEMQQIEQSNFRQAPQRAPGVADLALSENW<br>AQEFLAAGDAVDVTQDYNETDWSQEFISEVTDPLSVSPARWAEEYLEQSEEKLWLGEPEGTATDRWYDEYHPEEDLQHTASDFVAKVDDPKLANSEFLKFVRQIGEGQVSLESGAGSG<br>RAQAEQWAAEFIQQQGTSDAWVDQFTRPVNTSALDMEFERAKSAIELQAELEEMAKRDAEAHPWLSDYDDLTSATYDKGYQFEEENPL
>DisProt|DP00492|uniprot|P10275|sp|P10275	344	GLPQQLPAPPDEDDSAAPSTLSLLGPTFPGLSSCSADLKDILSEASTMQLLQQQQQEAVSEGSSSGRAREASGAPTSSKDNYLGGTSTISDNAKELCKAVSVSMGLGVEALEHLSPGE<br>QLRGDCMYAPLLGVPPAVRPTPCAPLAECKGSLLDDSAGKSTEDTAEYSPFKGGYTKGLEGESLGCSGSAAAGSSGTLELPSTLSLYKSGALDEAAAYQSRDYYNFPLALAGPPPPPP<br>PPHPHARIKLENPLDYGSAWAAAAAQCRYGDLASLHGAGAAGPGSGSPSAAASSSWHTLFTAEEGQLYGPCGGGGGGGGGGGGGGGGGGGGGGGGEAGAVAPYGYTRP
>DisProt|DP00540|uniprot|Q9VPU8|unigene|Dm.2087|sp|Q9VPU8_DROME	345	MSESEAEETKISTEPVDNAWSMKIPAFRQEDNPHGMVEESSFATLFPKYRERYLKEVWPLVEQCLAEHHLKAELDLMEGSMVVKTSRKTWDPYIIIKARDMIKLMARSVPFEQAKRVL<br>QDDIGCDIIKIGNLVHKKEKFVKRRQRLIGPNGATLKSIELLTDCYVLVQGNTVSALGPYKGLQQVRDIVLETMNNVHPIYNIKALMIKRELMKDPRLANEDWSRFLPKFKNKNISKR<br>KQPKVKKQKKEYTPFPPSQPESKVDKQLASGEYFLNQEQKQAKRNQERTEKQKEAAKRQDERRNKDFVPPTEESAASSRKKEDGSSSSKVDVKALKAKLIKANKKARSS
>DisProt|DP00181|uniprot|P08775|unigene|Mm.16533|sp|RPB1_MOUSE	368	SYSPTSPAYEPRSPGGYTPQSPSYSPTSPSYSPTSPSYSPTSPNYSPTSPSYSPTSPSYSPTSPSYSPTSPSYSPTSPSYSPTSPSYSPTSPSYSPTSPSYSPTSPSYSPTSPSYSPT<br>SPSYSPTSPSYSPTSPSYSPTSPSYSPTSPSYSPTSPNYSPTSPNYTPTSPSYSPTSPSYSPTSPNYTPTSPNYSPTSPSYSPTSPSYSPTSPSYSPSSPRYTPQSPTYTPSSPSYSP<br>SSPSYSPTSPKYTPTSPSYSPSSPEYTPASPKYSPTSPKYSPTSPKYSPTSPTYSPTTPKYSPTSPTYSPTSPVYTPTSPKYSPTSPTYSPTSPKYSPTSPTYSPTSPKGSTYSPTSP<br>GYSPTSPTYSLTSP
>DisProt|DP00328|uniprot|Q08605-2|unigene|Dm.7610|sp|GAGA_DROME	383	MIPQHHDQDQLIATIATAPQQTVHAQVVEDIHHQGQILQATTQTNAAGQQQTIVTTDAAKHDQAVIQAFLPARKRKPRVKKMSPTAPKISKVEGMDTIMGTPTSSHGSGSVQQVLGEN<br>GAEGQLLSSTPIIKSEGQKVETILTMDPNNMIPVTSANAATGEITPAQGATGSSGGNTSGVLSTPKAKRAKHPPGTEKPRSRSQSEQPATCPICYAVIRQSRNLRRHLELRHFAKPGV<br>KKEKKSKSGNDTTLDSSMEMNTTAEGDNTVGSDGAGGAGSAGGQSSGTTPTRVISNAPQAAGAPAILAQGVLPQQQQQQQLQQQHQQHLTATLAGGGQAYIKHEGGGGGGTGQQQQQQ<br>AAQQQGMQNVIHIVGDQVFIPQQQQPQPQ
>DisProt|DP00519|uniprot|P25054|unigene|Hs.158932|sp|P25054	384	SKSGAQTPKSPPEHYVQETPLMFSRCTSVSSLDSFESRSIASSVQSEPCSGMVSGIISPSDLPDSPGQTMPPSRSKTPPPPPQTAQTKREVPKNKAPTAEKRESGPKQAAVNAAVQRV<br>QVLPDADTLLHFATESTPDGFSCSSSLSALSLDEPFIQKDVELRIMPPVQENDNGNETESEQPKESNENQEKEAEKTIDSEKDLLDDSDDDDIEILEECIISAMPTKSSRKAKKPAQT<br>ASKLPPPVARKPSQLPVYKLLPSQNRLQPQKHVSFTPGDDMPRVYCVEGTPINFSTATSLSDLTIESPPNELAAGEGVRGGAQSGEFEKRDTIPTEGRSTDEAQGGKTSSVTIPELDD<br>NKAEEGDILAECINSAMPKGKSHKPFRVKK
>DisProt|DP00132|uniprot|P07221|unigene|Ocu.6275|sp|CASQ1_RABIT	395	MNAADRMGARVALLLLLLVLGSPQSGVHGEEGLDFPEYDGVDRVINVNAKNYKNVFKKYEVLALLYHEPPEDDKASQRQFEMEELILELAAQVLEDKGVGFGLVDSEKDAAVAKKLGLT<br>EEDSIYVFKEDEVIEYDGEFSADTLVEFLLDVLEDPVELIEGERELQAFENIEDEIKLIGYFKNKDSEHYKAFKEAAEEFHPYIPFFATFDSKVAKKLTLKLNEIDFYEAFMEEPVTI<br>PDKPNSEEEIVNFVEEHRRSTLRKLKPESMYETWEDDMDGIHIVAFAEEADPDGYEFLEILKSVAQDNTDNPDLSIIWIDPDDFPLLVPYWEKTFDIDLSAPQIGVVNVTDADSVWME<br>MDDEEDLPSAEELEDWLEDVLEGEINTEDDDDEDDDDDDDD
>DisProt|DP00333|uniprot|P27797|unigene|Hs.515162|sp|CALR_HUMAN	400	EPAVYFKEQFLDGDGWTSRWIESKHKSDFGKFVLSSGKFYGDEEKDKGLQTSQDARFYALSASFEPFSNKGQTLVVQFTVKHEQNIDCGGGYVKLFPNSLDQTDMHGDSEYNIMFGPD<br>ICGPGTKKVHVIFNYKGKNVLINKDIRCKDDEFTHLYTLIVRPDNTYEVKIDNSQVESGSLEDDWDFLPPKKIKDPDASKPEDWDERAKIDDPTDSKPEDWDKPEHIPDPDAKKPEDW<br>DEEMDGEWEPPVIQNPEYKGEWKPRQIDNPDYKGTWIHPEIDNPEYSPDPSIYAYDNFGVLGLDLWQVKSGTIFDNFLITNDEAYAEEFGNETWGVTKAAEKQMKDKQDEEQRLKEEE<br>EDKKRKEEEEAEDKEDDEDKDEDEEDEEDKEEDEEEDVPGQAKDEL

Table S7. PCA decomposition of native, denatured, and combined TEs. The matrices represent decomposition of the combined data from archaea, bacteria, and eukaryotes, as described in Main Text Methods. Center represents the average frequencies of each TE over the combined data. Information content is the fractional amount of the sum of the squared eigenvalues for each eigenvector (*i.e.* Principal Component, PC).

Native TE	NTE1	NTE2	NTE3	NTE4	NTE5	NTE6	NTE7	NTE8	Information Content
Center	0.014810973	0.106598365	0.136965865	0.217443187	0.256477227	0.091647998	0.141424361	0.034629457	
PC1	-0.144077902	-0.567834772	0.261605342	-0.502495954	0.022203224	0.374059332	0.420835843	0.135460391	0.7256
PC2	0.113304783	0.314717688	0.391929965	-0.534500101	0.131176948	0.224500692	-0.61692516	-0.024713201	0.219506
PC3	-0.048470244	-0.223378741	-0.54392268	0.012396849	0.728931658	0.239169423	-0.240106356	0.07451559	0.02378
PC4	0.297075122	0.282360781	-0.582680124	-0.329101408	-0.4129687	0.278629254	0.113083482	0.351040759	0.02149
PC5	-0.087781204	-0.282817697	0.055474415	0.476337761	-0.39297224	0.604085694	-0.402717823	0.028375786	0.007052
PC6	0.676227464	-0.167221261	-0.055551619	-0.020358732	0.008874432	0.149518032	0.105222352	-0.69119235	0.001682
PC7	-0.533559231	0.466238414	-0.106567819	-0.030947302	0.036536187	0.404513385	0.273791467	-0.495675412	0.00084
PC8	0.355142247	0.351516313	0.353645933	0.353661084	0.352950826	0.352046212	0.35163397	0.357786791	5.07E-05

Denatured TE	DTE1	DTE2	DTE3	DTE4	DTE5	DTE6	DTE7	DTE8	Information Content
Center	0.127648949	0.080502139	0.096571266	0.062792621	0.351477132	0.130692118	0.054914234	0.095409337	
PC1	-0.10059753	-0.041970005	-0.47382059	-0.416659963	0.019506212	0.574398065	-0.065967475	0.505313608	0.759167491
PC2	0.517735572	0.397193324	-0.219503448	-0.099647597	0.169571056	0.183537537	-0.514792596	-0.434308815	0.154332003
PC3	0.427288287	-0.065539695	0.425492319	-0.215308932	-0.711538267	0.075415668	-0.155762689	0.222466571	0.035410359
PC4	-0.382363641	0.396207206	-0.158316873	0.491253692	-0.523802345	0.355709612	-0.007323551	-0.17167299	0.028496418
PC5	0.114589884	0.566370196	-0.284366927	-0.358315233	-0.189943141	-0.393976752	0.514430033	0.030087667	0.012899358
PC6	-0.4749721	0.339210415	0.561688951	-0.495628694	0.154013283	0.185420168	-0.093149992	-0.177250859	0.00672619
PC7	0.171717305	-0.344016846	-0.01035494	-0.172866773	-0.071310017	0.435095032	0.554367453	-0.566091293	0.0029201
PC8	-0.353322247	-0.353573028	-0.353182796	-0.353349936	-0.354014062	-0.353918038	-0.354517662	-0.352545788	4.80814E-05

Native And Denatured TE	NTE1	NTE2	NTE3	NTE4	NTE5	NTE6	NTE7	NTE8	DTE1	DTE2	DTE3	DTE4	DTE5	DTE6	DTE7	DTE8	Information Content
Center	0.015	0.107	0.137	0.217	0.256	0.092	0.141	0.035	0.128	0.081	0.097	0.063	0.351	0.131	0.055	0.095	
PC1	0.108	0.399	-0.189	0.327	-0.008	-0.246	-0.304	-0.087	-0.030	-0.013	-0.351	-0.317	0.029	0.421	-0.081	0.342	0.699
PC2	-0.073	-0.107	-0.272	0.513	-0.118	-0.261	0.351	-0.033	0.272	0.270	-0.077	0.088	0.157	-0.008	-0.289	-0.413	0.167
PC3	-0.044	-0.382	-0.319	0.002	0.003	0.111	0.485	0.144	-0.465	-0.048	-0.295	0.065	0.036	0.263	0.183	0.260	0.074
PC4	0.093	0.036	0.112	-0.199	-0.418	0.142	0.130	0.104	0.152	0.274	-0.012	0.061	-0.634	0.397	-0.231	-0.007	0.024
PC5	0.176	0.396	-0.083	0.050	-0.478	-0.308	0.218	0.029	-0.161	-0.452	0.218	0.354	-0.007	-0.118	0.116	0.050	0.011
PC6	0.021	0.046	0.067	0.276	-0.330	0.066	-0.132	-0.016	-0.166	0.434	-0.186	-0.107	-0.164	-0.349	0.608	-0.071	0.007
PC7	-0.068	-0.249	0.446	0.318	0.085	-0.332	0.131	-0.329	-0.313	0.073	0.397	-0.136	-0.199	0.049	-0.108	0.239	0.006
PC8	0.044	-0.093	-0.372	0.363	0.025	0.396	-0.371	0.006	-0.234	-0.141	0.448	0.089	-0.159	0.229	0.019	-0.250	0.005
PC9	-0.120	-0.070	-0.211	0.191	0.379	-0.144	-0.065	0.040	0.294	-0.120	-0.139	0.384	-0.545	-0.259	0.085	0.300	0.003
PC10	0.195	0.124	0.085	-0.142	0.227	-0.184	-0.227	-0.082	-0.455	0.350	-0.203	0.572	0.051	0.061	-0.179	-0.200	0.002
PC11	0.242	0.231	-0.490	-0.221	0.127	0.010	0.186	-0.077	-0.122	0.382	0.333	-0.246	-0.060	-0.334	-0.192	0.241	0.001
PC12	-0.162	-0.145	0.089	0.187	-0.314	0.134	-0.258	0.465	-0.100	0.097	-0.002	0.129	0.198	-0.279	-0.440	0.404	0.001
PC13	0.433	-0.182	0.036	0.108	-0.176	0.392	0.016	-0.617	0.151	-0.107	-0.213	0.164	0.106	-0.141	-0.152	0.199	0.000
PC14	0.696	-0.449	-0.021	-0.038	-0.004	-0.352	-0.178	0.333	0.117	-0.054	0.040	-0.132	-0.011	0.005	0.089	-0.055	0.000
PC15	0.065	0.064	0.064	0.064	0.066	0.062	0.064	0.065	0.347	0.348	0.348	0.347	0.347	0.349	0.350	0.345	0.000
PC16	0.349	0.346	0.348	0.348	0.347	0.346	0.345	0.352	-0.064	-0.065	-0.065	-0.064	-0.064	-0.063	-0.064	-0.066	0.000

Table S8. Primary sequence data for 35 proteins used for bootstrapping stability prediction.

1aps	STARPLKSVDYEVFGRVQGVCFRMYAEDEARKIGVVGWVKNTSKGTVTGQVQGPEEKVNSMKSWLSKVGSPSSRIDRTNFSNEKTISKLEYSNFSVRY
1avz	TLFVALYDYEARTEDDLSFHKGEKFQILNSSEGDWWEARSLTTGETGYIPSNYVAPV
1ayi	MELKNSISDYTEAEFVQLLKEIEKENVAATDDVLDVLLEHFVKITEHPDGTDLIYYPSDNRDDSPEGIVKEIKEWRAANGKPGFKQ
1divC	AAEELANAKKLKEQLEKLTVTIPAKAGEGGRLFGSITSKQIAESLQAQHGLKLDKRKIELADAIRALGYTNVPVKLHPEVTATLKVHVTEQK
1divN	MKVIFLKDVKGKGKKGEIKNVADGYANNFLFKQGLAIEATPANLKALEAQKQKEQR
1e65	AECSVDIQGNDQMQFNTNAITVDKSCKQFTVNLSHPGNLPKNVMGHNWVLSTAADMQGVVTDGMASGLDKDYLKPDDSRVIAHTKLIGSGEKDSVTFDVSKLKEGEQYMFFCTFPGHSALMKGTLTLK
1fkf	GVQVETISPGDGRTFPKRGQTCVVHYTGMLEDGKKFDSSRDRNKPFKFMLGKQEVIRGWEEGVAQMSVGQRAKLTISPDYAYGATGHPGIIPPHATLVFDVELLKLE
1imq	MELKHSISDYTEAEFLQLVTTICNADTSSEEELVKLVTHFEEMTEHPSGSDLIYYPKEGDDDSPSGIVNTVKQWRAANGKSGFKQG
1jo8	PWATAEYDYDAAEDNELTFVENDKIINIEFVDDDWWLGELEKDGSKGLFPSNYVSLGN
1k0s	MKTLADALKEFEVLSFEIDEQALAFDVDNIEMVIEKSDITPVPKSRHFVEGVINLRGRIIPVVNLAKILGISFDEQKMKSIIVARTKDVEVGFLVDRVLGVLRITENQLDLTNVSDKFGKKSKGLVKTDGRLIIYLDIDKIIEEITVKEGV
1l8w	PTNKFYQSVIQLGNGFLDVFTSFGGLVAEAFGFKSDPKKSDVKTYFTTVAAKLEKTKTDLNSLPTAVEGAIKEVSELLDKLVKAVKTAEGASSGTAAIGEVVADADAAKVADKASVKGIAKGIKEIVEAAGGSEKLKAVAAAKGENNKGAGKLFGKAGAAAHGDSEAASKAAGAVSAVSGEQILSAIVTAADAAEQDGKKPEEAKNPIAAAIGDKDGGAEFGQDEMKKDDQIAAAIALRGMAKDGKFAVKDGEKEKAEGAIKGAAESAVRKVLGAITGLIGDAVSSGLRKVGDSVK
1lmb	PLTQEQLEDARRLKAIYEKKKNELGLSQESVADKMGMGQSGVGALFNGINALNAYNAALLAKILKVSVEEFSPSIAREIYEMYEAVS
1m9s	ETTEKTVNLTRYVKYIRGNAGIYKLPREDNSLKQGTLASHRCKALTVDREARNGGKLWYRLKNIGWTKAENLSLDR
1n88	MKTAYDVILAPVLSEKAYAGFAEGKYTFWVHPKATKTEIKNAVETAFKVKVVKVNTLHVRGKKKRLGRYLGKRPDRKKAIVQVAPGQKIEALEGLI
1nti	SQAEFDKAAEEVKHLKTKPADEEMLFIYSHYKQATVGDINTERPGMLDFKGKAKWDAWNELKGTSKEDAMKAYIDKVEELKKKYGI
1o6x	MRSLETFVGDQVLEIVPSNEEQIKNLLQLEAQEHLQLDFWKSPTTPGETAHVRVPFVNVQAVKVFLESQGIAYSIMIEDVQ
1rfa	SNTIRVFLPNKQRTVVNVRNGMSLHDCLMKALKVRGLQPECCAVFRLLHEHKGKKARLDWNTDAASLIGEELQVDFLD
1ris	MRRYEVNIVLNPNLDQSQLALEKEIIQRALENYGARVEKVEELGLRRLAYPIAKDPQGYFLWYQVEMPEDRVNDLARELRIRDNVRRVMVVKSQEPF
1rlq	TFVALYDYESRTETDLSFKKGERLQIVNNTEGDWWLAHSLTTGQTGYIPSNYVAPS
1ryk	MNKDEAGGNWKQFKGKVKEQWGKLTDDDMTIIEGKRDQLVGKIQERYGYQKDQAEKEVVDWETRNEYRW
1shg	KELVLALYDYQEKSPREVTMKKGDILTLLNSTNKDWWKVEVNDRQGFVPAAYVKKLD
1spr	AEEWYFGKITRRESERLLLNPENPRGTFLVRESETTKGAYCLSVSDFDNAKGLNVKHYKIRKLDSGGFYITSRTQFSSLQQLVAYYSKHADGLCHRLTNVCPT
1ubq	MQIFVKTLTGKTITLEVEPSDTIENVKAKIQDKEGIPPDQQRLIFAGKQLEDGRTLSDYNIQKESTLHLVLRLRGG
1urn	AVPETRPNHTIYINNLNEKIKKDELKKSLHAIFSRFGQILDILVSRSLKMRGQAFVIFKEVSSATNALRSMQGFPFYDKPMRIQYAKTDSDIIAKM
2ci2	NLKTEWPELVGKSVEEAKKVILQDKPEAQIIVLPVGTIVTMEYRIDRVRLFVDKLDNIAEVPRVG
2ptl	ENKEETPETPETDSEEEVTIKANLIFANGSTQTAEFKGTFEKATSEAYAYADTLKKDNGEYTVDVADKGYTLNIKFAG
3gb1	MTYKLILNGKTLKGETTTEAVDAATAEKVFKQYANDNGVDGEWTYDDATKTFTVTE
1ey0	ATSTKKLHKEPATLIKAIDGDTVKLMYKGQPMTFRLLLVDTPETKHPKKGVEKYGPEASAFTKKMVENAKKIEVEFDKGQRTDKYGRGLAYIYADGKMVNEALVRQGLAKVAYVYKPNNTHEQHLRKSEAQAKKEKLNIWSEDNADSGQ
EXG:CBM	ASSGPAGCQVLWGVNQWNTGFTANVTVKNTSSAPVDGWTLTFSFPSGQQVTQAWSSTVTQSGSAVTVRNAPWNGSIPAGGTAQFGFNGSHTGTNAAPTAFSLNGTPCTVG
hGRA-NTD	MDSKESLTPGREENPSSVLAQERGDVMDFYKTLRGGATVKVSASSPSLAVASQSDSKQRRLLVDFPKGSVSNAQQPDLSKAVSLSMGLYMGETETKVMGNDLGFPQQGQISLSSGETDLKLLEESIANLNRSTSVPENPKSSASTAVSAAPTEKEFPKTHSDVSSEQQHLKGQTGTNGGNVKLYTTDQSTFDILQDLEFSSGSPGKETNESPWRSDLLIDENCLLSPLAGEDDSFLLEGNSNEDCKPLILPDTKPKIKDNGDLVLSSPSNVTLPQVKTEKEDFIELCTPGVIKQEKLGTVYCQASFPGANIIGNKMSAISVHGVSTSGGQMYHYDMNTASLSQQQDQKPIFNVIPPIPVGSENWNRCQGSGDDNLTSLGTLNFPGRTVFSNGYSSPSMRPDVSSPPSSSSTATTGPPPKL
hGRC2-NTD	MGETETKVMGNDLGFPQQGQISLSSGETDLKLLEESIANLNRSTSVPENPKSSASTAVSAAPTEKEFPKTHSDVSSEQQHLKGQTGTNGGNVKLYTTDQSTFDILQDLEFSSGSPGKETNESPWRSDLLIDENCLLSPLAGEDDSFLLEGNSNEDCKPLILPDTKPKIKDNGDLVLSSPSNVTLPQVKTEKEDFIELCTPGVIKQEKLGTVYCQASFPGANIIGNKMSAISVHGVSTSGGQMYHYDMNTASLSQQQDQKPIFNVIPPIPVGSENWNRCQGSGDDNLTSLGTLNFPGRTVFSNGYSSPSMRPDVSSPPSSSSTATTGPPPKL
hGRC3-NTD	MGNDLGFPQQGQISLSSGETDLKLLEESIANLNRSTSVPENPKSSASTAVSAAPTEKEFPKTHSDVSSEQQHLKGQTGTNGGNVKLYTTDQSTFDILQDLEFSSGSPGKETNESPWRSDLLIDENCLLSPLAGEDDSFLLEGNSNEDCKPLILPDTKPKIKDNGDLVLSSPSNVTLPQVKTEKEDFIELCTPGVIKQEKLGTVYCQASFPGANIIGNKMSAISVHGVSTSGGQMYHYDMNTASLSQQQDQKPIFNVIPPIPVGSENWNRCQGSGDDNLTSLGTLNFPGRTVFSNGYSSPSMRPDVSSPPSSSSTATTGPPPKL
P-protein	MAHLKKRNRLKKNEDFQKVFKHGTSVANRQFVLYTLDQPENDELRVGLSVSKKIGNAVMRNRIKRLIRQAFLEEKERLKEKDYIIIARKPASQLTYEETKKSLQHLFRKSSLYKKSSSK
a-synuclein	MDVFMKGLSKAKEGVVAAAEKTKQGVAEAAGKTKEGVLYVGSKTKEGVVHGVATVAEKTKEQVTNVGGAVVTGVTAVAQKTVEGAGSIAAATGFVKKDQLGKNEEGAPQEGILEDMPVDPDNEAYEMPSEEGYQDYEPEA
RCAM-T1	ACDYTCGSNCYSSSDVSTAQAAGYQLHEDGETVGSNSYPHKYNNYEGFDFSVSSPYYEWPILSSGDVYSGGSPGADRVVFNENNQLAGVITHTGASGNNFVECT

Figure S1. Protein Stability Prediction Using Thermodynamic Environments. Plots A) and B) indicate values taken from Table S5. Purple columns are Table S5 Full Set row, and blue columns are Table S5 Averages with error bars as Standard Deviations. The native state weights suggest greater importance of the more stable TEs for experimental protein stability. The denatured state weights suggest that denatured state environments of moderate stability have greater weight than do extremes of denatured state stability, with the exception of low stability denatured state environment 2. C) Distributions of predicted stabilities for the 262 structured and 262 intrinsically disordered proteins given in Table S6, using Main Text Equation (4). In general, structured proteins are predicted to be more stable than are intrinsically disordered proteins, with a median stability of ~ 20 kJ/mol for structured proteins and a median stability of ~ 0 kJ/mol for intrinsically disordered proteins.

A.

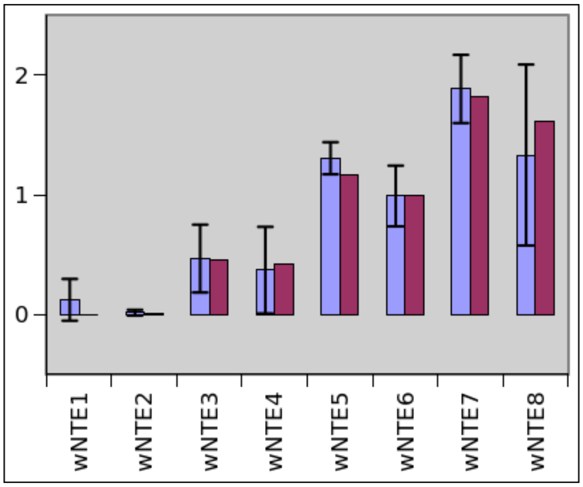

B.

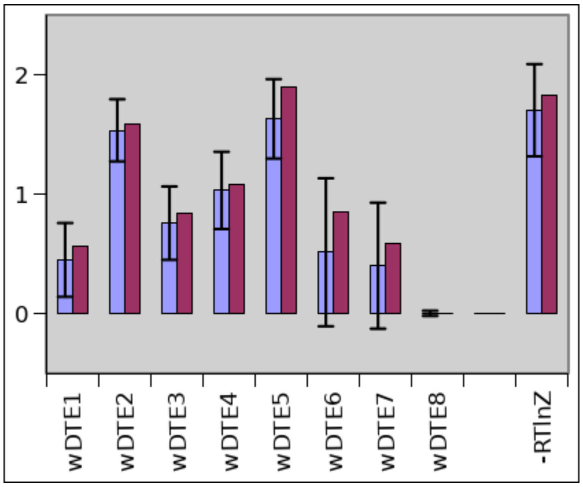

C.

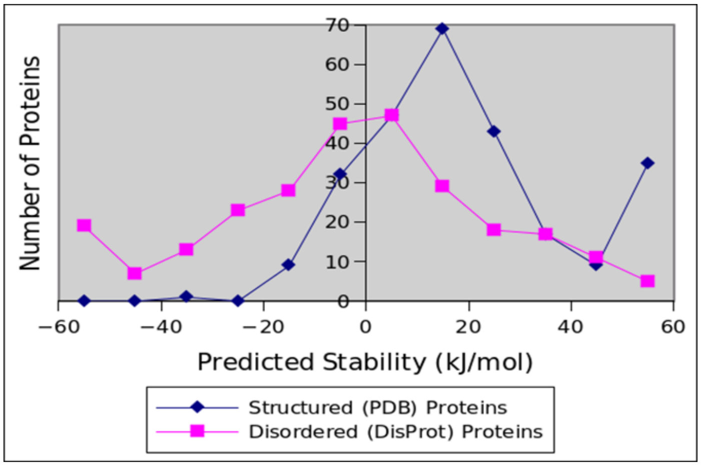

Figure S2. Thermodynamic Interpretations of PC1 and PC2 are ΔG and ΔH_{apolar}, respectively. Average Native State ΔG and ΔH_{apolar} values are taken from Figure 5 of (Hoffmann, et al. 2016) and values for Native State PC1 and PC2 are given in Table S7.

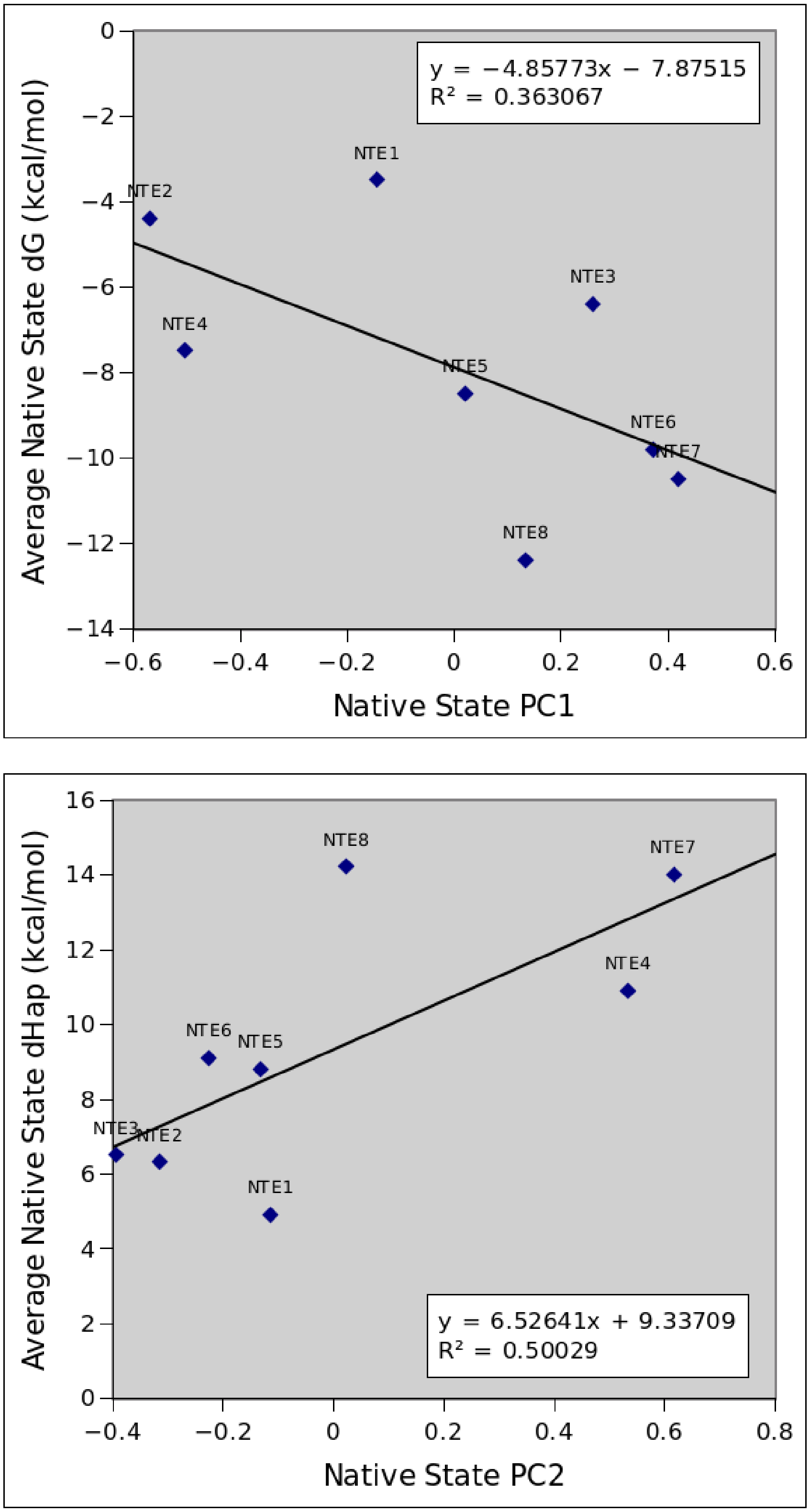

Figure S3. Physical Interpretation of PC2 is Proteome Hydrophobicity. For idealized poly-amino acid proteins composed of a single amino acid type, the *eScape* algorithm predicts expected ΔH_{apolar} values for each residue type (x-axis). Since Native State PC2 correlates with Native State Average ΔH_{apolar} (Figure S3), we postulate that Native State PC2 reflects a hydrophobicity component of a proteome, exemplified by a well-known octanol-water transfer free energy scale (Radzicka and Wolfenden 1988) (y-axis). For unknown reasons, Arginine is an outlier in these data.

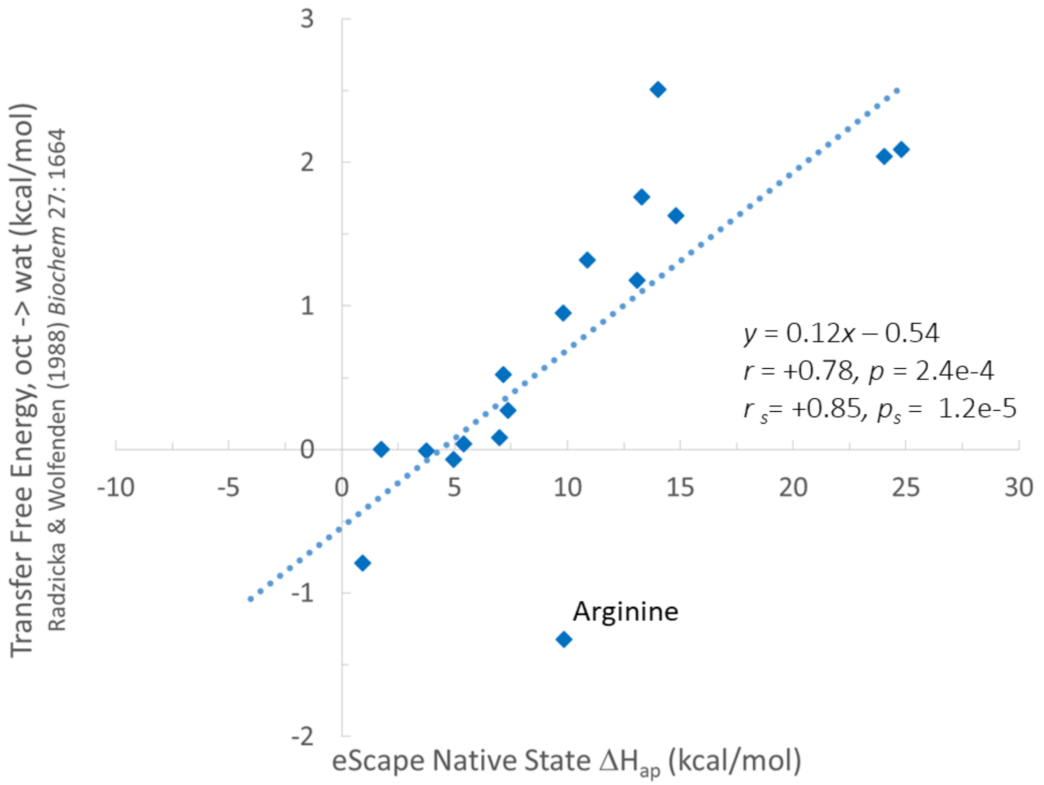

Figure S4. Principal components analysis of TE occurrence frequency, by proteome, including both native and denatured states. The combined native and denatured PCA matrix given in Table S7 was used to compute these data. Although the three kingdoms locate in space similarly to Main Text Figure 4, the thermodynamic properties of eukaryotes are even more separated from those of bacteria and archaea. Several Asgard archaea (large points) (Spang, et al. 2015), unexpectedly do not occupy a boundary between eukaryotes and bacteria, suggesting that the thermodynamic properties of archaea are not transitional.

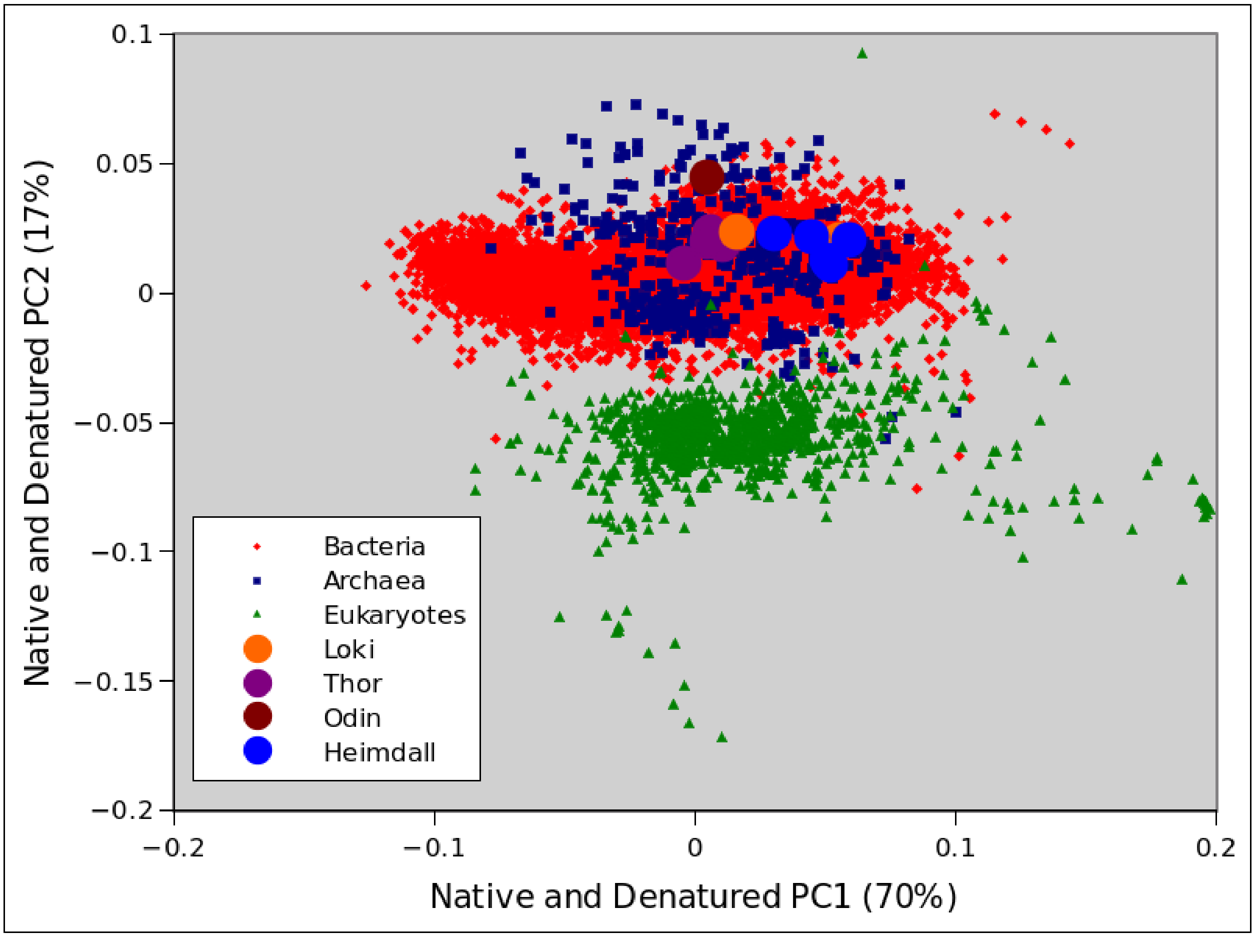

Supplementary Material References

Berlow RB, Dyson HJ, Wright PE. 2018. Expanding the Paradigm: Intrinsically Disordered Proteins and Allosteric Regulation. J Mol Biol 430:2309-2320.

Cheng H, Schaeffer RD, Liao Y, Kinch LN, Pei J, Shi S, Kim BH, Grishin NV. 2014. ECOD: an evolutionary classification of protein domains. PLoS Comput Biol 10:e1003926.

Engqvist MKM. 2018. Correlating enzyme annotations with a large set of microbial growth temperatures reveals metabolic adaptations to growth at diverse temperatures. BMC Microbiol 18:177.

Gu J, Hilser VJ. 2008. Predicting the energetics of conformational fluctuations in proteins from sequence: a strategy for profiling the proteome. Structure 16:1627-1637.

Hatos A, Hajdu-Soltesz B, Monzon AM, Palopoli N, Alvarez L, Aykac-Fas B, Bassot C, Benitez GI, Bevilacqua M, Chasapi A, et al. 2020. DisProt: intrinsic protein disorder annotation in 2020. Nucleic Acids Res 48:D269-D276.

Hilser VJ, Freire E. 1996. Structure-based calculation of the equilibrium folding pathway of proteins. Correlation with hydrogen exchange protection factors. J Mol Biol 262:756-772.

Hoffmann J, Wrabl JO, Hilser VJ. 2016. The role of negative selection in protein evolution revealed through the energetics of the native state ensemble. Proteins 84:435-447.

Larson SA, Hilser VJ. 2004. Analysis of the "thermodynamic information content" of a Homo sapiens structural database reveals hierarchical thermodynamic organization. Protein Sci 13:1787-1801.

Liu T, Pantazatos D, Li S, Hamuro Y, Hilser VJ, Woods VL, Jr. 2012. Quantitative assessment of protein structural models by comparison of H/D exchange MS data with exchange behavior accurately predicted by DXCOREX. J Am Soc Mass Spectrom 23:43-56.

Miralles F. 2010. Compositional properties and thermal adaptation of SRP-RNA in bacteria and archaea. J Mol Evol 70:181-189.

Radzicka A, Wolfenden R. 1988. Comparing the polarities of the amino acids: side-chain distribution coefficients between the vapor phase, cyclohexane, 1-octanol, and neutral aqueous solution. Biochemistry 27:1664-1670.

Sauer DB, Karpowich NK, Song JM, Wang DN. 2015. Rapid Bioinformatic Identification of Thermostabilizing Mutations. Biophys J 109:1420-1428.

Spang A, Saw JH, Jorgensen SL, Zaremba-Niedzwiedzka K, Martijn J, Lind AE, van Eijk R, Schleper C, Guy L, Ettema TJG. 2015. Complex archaea that bridge the gap between prokaryotes and eukaryotes. Nature 521:173-179.

Wang S, Gu J, Larson SA, Whitten ST, Hilser VJ. 2008. Denatured-state energy landscapes of a protein structural database reveal the energetic determinants of a framework model for folding. J Mol Biol 381:1184-1201.

Ward JJ, Sodhi JS, McGuffin LJ, Buxton BF, Jones DT. 2004. Prediction and functional analysis of native disorder in proteins from the three kingdoms of life. J Mol Biol 337:635-645.

Wrabl JO, Larson SA, Hilser VJ. 2002. Thermodynamic environments in proteins: fundamental determinants of fold specificity. Protein Sci 11:1945-1957.

Wrabl JO, Larson SA, Hilser VJ. 2001. Thermodynamic propensities of amino acids in the native state ensemble: implications for fold recognition. Protein Sci 10:1032-1045.
